# The CINSARC signature as a prognostic marker for clinical outcome in multiple neoplasms

**DOI:** 10.1038/s41598-017-05726-x

**Published:** 2017-07-14

**Authors:** Tom Lesluyes, Lucile Delespaul, Jean-Michel Coindre, Frédéric Chibon

**Affiliations:** 1 INSERM U1218, Institut Bergonié, F-33000 Bordeaux, France; 2 University of Bordeaux, F-33000 Bordeaux, France; 3 Department of Pathology, Institut Bergonié, F-33000 Bordeaux, France

## Abstract

We previously reported the CINSARC signature as a prognostic marker for metastatic events in soft tissue sarcomas, breast carcinomas and lymphomas through genomic instability, acting as a major factor for tumor aggressiveness. In this study, we used a published resource to investigate CINSARC enrichment in poor outcome-associated genes at pan-cancer level and in 39 cancer types. CINSARC outperformed more than 15,000 defined signatures (including cancer-related), being enriched in top-ranked poor outcome-associated genes of 21 cancer types, widest coverage reached among all tested signatures. Independently, this signature demonstrated significant survival differences between risk-groups in 33 published studies, representing 17 tumor types. As a consequence, we propose the CINSARC prognostication as a general marker for tumor aggressiveness to optimize the clinical managements of patients.

## Introduction

From the first report of gene expression quantification method by Schena *et al*. in 1995^1^, to RNA sequencing (RNA-seq), extensively used nowadays by international consortia to decipher transcriptomic abnormalities^2, 3^, gene expression has become an essential tool in cancer research. For two decades, microarrays provided much information, both at gene and transcript levels, on various oncogenic factors^4–7^. Following on, the next-generation sequencing (NGS) permitted sequencing of RNA fragments, to a single base-pair resolution, where RNA abundance is directly related to the proportion of sequenced reads mapped to a given gene^8^. Moreover, dedicated RNA-seq algorithms allow obtaining genomic information such as point mutations, insertions/deletions, translocations and genomic integrations from foreign organisms: well-known oncogenic and tumor progression mechanisms^9–11^. However, standard measurements are yet to be defined since numerous software exist for RNA-seq processing, including many different gene expression normalization methods^12^.

Such transcriptomic investigations generated a large amount of data. Consequently, databases were set up to standardize all information associated with expression matrices, notably: organism, platform identifier, normalization, unit measurement and study-specific information (*i.e*. cell types, treatments, time series, sampling and culture conditions, etc.). Among the many available gene expression databases, the two most used are Gene Expression Omnibus from the National Center for Biotechnology Information (http://www.ncbi.nlm.nih.gov/geo)^13^ and ArrayExpress from the European Bioinformatics Institute (http://www.ebi.ac.uk/arrayexpress)^14^. These resources, by gathering results from microarrays and RNA-seq experiments, provide an easy access to millions of cancer-related transcriptomic profiles (cell lines, primary tumors and metastases/relapses).

Since Golub *et al*. identified a specific gene set capable of distinguishing acute myeloid leukemia from acute lymphoblastic leukemia in the late 90s^15^, establishment of gene expression signature remains a key part of cancer research. This first study demonstrated the possibility to use gene expression as a new *in silico* classifier, whereas previous options were limited to clinical observations and immunohistochemistry experiments. Subsequently, multiple gene sets were defined, not only to differentiate entities, but also to try predicting disease evolution. Two publications in early 2000s demonstrated the usefulness of transcriptomic profile as a survival indicator in breast cancer by focusing on specific genes^16, 17^. Few years later, a two-gene expression ratio was found to be a good predictor of tamoxifen response for breast cancer^18^. Then, inferring chromosomal instability from gene expression has become a promising predictor of clinical outcome in various cancers^19^. To date, the prognostic values of specific gene expression signatures have been demonstrated for breast cancer, lymphoma, leukemia, hepatocellular carcinoma, sarcoma, etc.^20–24^.

In 2010, we defined a set of 67 genes as a predictor for metastatic events in sarcomas with complex genetics^24^, with a better prognosis compared to the standard FNCLCC (*Fédération Nationale des Centres de Lutte Contre le Cancer*) grading system based on tumor differentiation, mitotic index and necrosis^25^. These genes were identified based on differential expression analyses with three different classifiers: FNCLCC grade, genomic alteration number and chromosomal instability signature^19^. Gene ontologies associated with these genes are mitosis and chromosome integrity pathways. This signature, called CINSARC (Complexity INdex in SARComas), was also informative in diffuse large B-cell lymphomas and breast carcinomas. We extended its application scope to sarcomas with simple genetics (harboring recurrent and specific genomic alteration): gastrointestinal stromal tumors and synovial sarcomas^26, 27^. Venet *et al*. demonstrated that randomly generated gene sets could be better predictors compared to published signatures in breast cancers^28^. Based on this observation, we compared the prognostic value of CINSARC to 1000 equal-sized randomly generated gene sets on four independent sarcoma datasets. We reported that no randomly generated signature was a better predictor than CINSARC^29^.

Recently, Gentles *et al*. reported an extensive data-mining analysis and compiled their results into a new resource named PRECOG (PREdiction of Clinical Outcomes from Genomic profiles; https://precog.stanford.edu)^30^. They evaluated the prognostic value of 23,287 genes across 39 cancer types from 166 published expression datasets, approximately representing 18,000 tumors. Experiments were performed on genes associated with good outcome, particularly genes involved in the immune system with a particular interest for the CIBERSORT (Cell type Identification By Estimating Relative Subsets Of known RNA Transcripts) approach^31^. Gentles *et al*. also observed that prognostic genes are significantly more shared by different tumor types than expected by chance, either associated with good or poor outcome. This highlights the possibility of using a non-cancer-specific signature as an outcome predictor, consisting of genes sharing same expression patterns across several tumor types.

Since we previously demonstrated that CINSARC is a significant prognostic factor in multiple malignancies, we analyzed the PRECOG resource and evaluated its prognostic ability in the available 39 cancer types. Furthermore, this signature has been compared to other gene sets defined by various methods (transcription factor regulated genes, cancer expression patterns, chromosomal positions, co-expression networks, specific immune processes, etc.). We address the following questions: is CINSARC a signature enriched in genes associated with poor outcome in a pan-cancer overview? Could CINSARC be applied to multiple cancer-specific studies such that, with additional validations, this signature could be tested in prospective studies to optimize the clinical management of patients?

## Results

### Signature enrichments at pan-cancer level

To obtain an exhaustive set of gene expression signatures, we used the MSigDB (Molecular Signatures DataBase; v5.2), including 18,026 gene sets, and the GeneSigDB (Gene Signature DataBase; v4.0), including 2,951 human gene sets^32, 33^. CINSARC has already been included in GeneSigDB (named *SARCOMA CHIBON10 67GENES GENOMECOMPLEXITYPREDICTOR*), but lacks several genes (62 instead of 67). Consequently, we included the full signature named *SARCOMA CHIBON10 67GENES GENOMECOMPLEXITYPREDICTOR CURATED*. We performed the popular Gene Set Enrichment Analysis (GSEA) method to determine signature enrichments in genes significantly associated to poor outcome (see methods)^34^. As recommended by GSEA documentation, we removed signatures with less than 25 known genes in PRECOG database to avoid inflated scorings for very small gene sets. This filtered out 4,394 (24%) and 1,085 (37%) signatures from MSigDB and GeneSigDB, respectively. Of note, *BORA* (formerly known as *C13orf34*) was not evaluated in PRECOG and is therefore missing in both full (curated) and reduced (62 genes) CINSARC.

**Figure 1:**
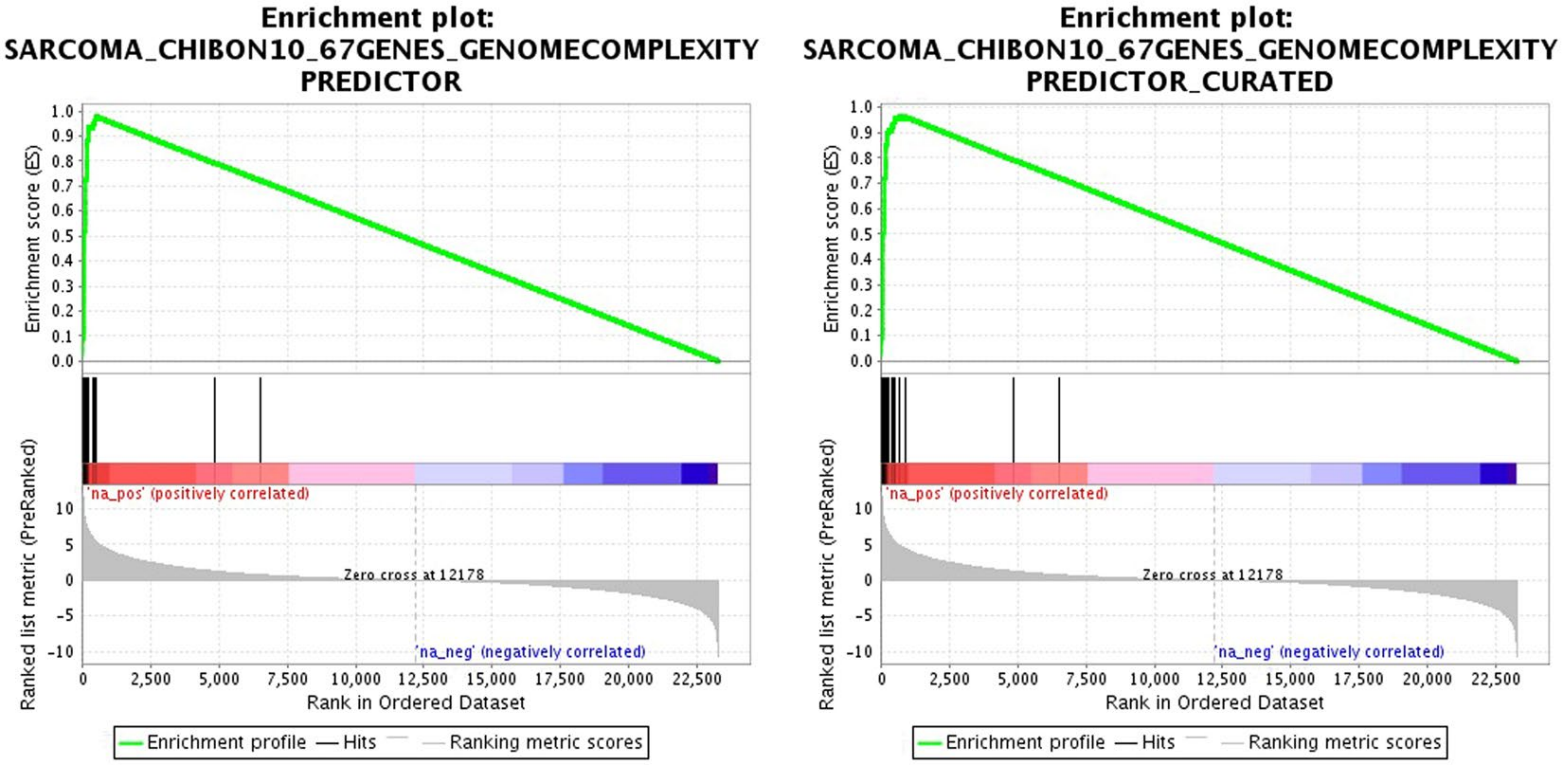
Enrichment plots of the reduced (left) and full (right) CINSARC signatures. Genes are ranked according to their individual prognosis given in PRECOG (poor and good prognoses in the left and right sides, respectively), represented by red and blue segments. Across these segments, CINSARC genes are marked by vertical black lines. The enrichment score (green plot) corresponds to a running-sum statistics: for each gene, if part of the signature, a positive value is added and a negative one otherwise.

**Figure 2:**
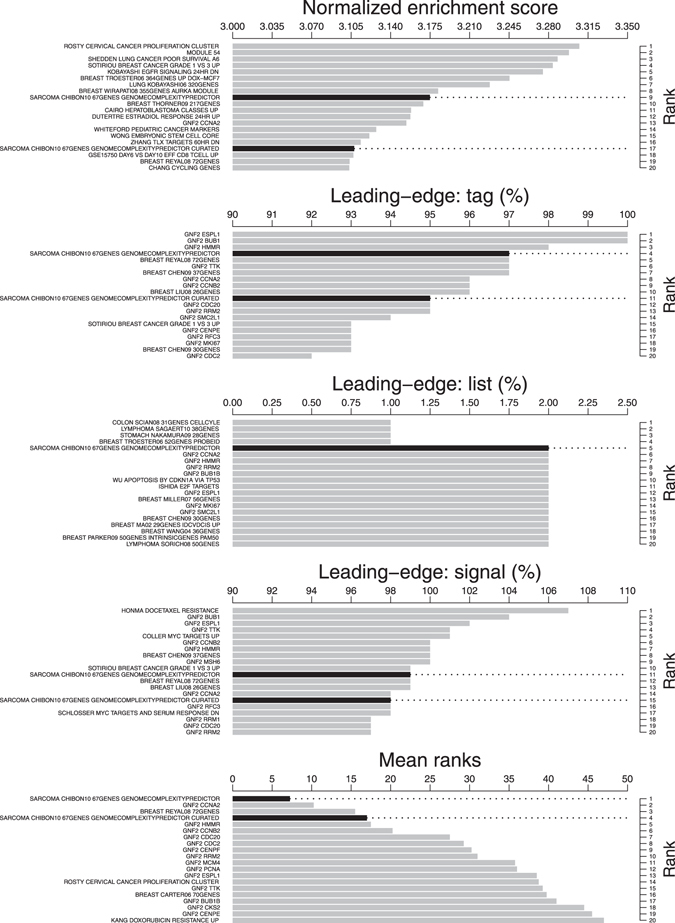
Pan-cancer overview of the top 20 signatures by several metrics (from top to bottom): normalized enrichment score, tag (sensitivity), list (false-negative rate), signal (enrichment strength) and the mean ranks of the previous metrics. Histograms are sorted to display best to worst ranks from top to bottom, respectively. CINSARC signatures are highlighted in black.

In the database of 15,499 gene sets, 1,273 (8%) were significantly enriched in genes associated with poor prognosis (family-wise error rate *P* < 0.05; Supplementary Table 1: sheet 1). Sorted by decreasing normalized enrichment scores (NES; primary statistic for examining gene set enrichment results; see methods), reduced and full CINSARC ranked 9 and 17, respectively (Figs 1 and 2). In addition to a high NES, leading-edges metrics (extension of the NES results; see methods) showed high sensitivity (tags are 97% and 95%, ranked 4 and 11 for reduced and full CINSARC, respectively), low false-negative rate (lists are 2% and 3%, ranked 5 and 25 for reduced and full CINSARC, respectively) and high enrichment signal strength (signals are 99% and 98%, ranked 11 and 15 for reduced and full CINSARC, respectively). All these four metrics combined (NES and the three leading-edge metrics), other signatures in the top 20 performed worst compared to CINSARC with a mean sensitivity of 76% (range: 65–97%), a mean false-negative rate of 7% (range: 2–13%) and a mean enrichment signal strength of 81% (range: 69–99%). Scored by the mean ranks of the four metrics, CINSARC obtained scores of 7.25 and 17, corresponding to ranks 1 and 4 for reduced and full CINSARC, respectively (Fig. 2). Of note, the top 10 consisted of another breast cancer signature defined by Reyal *et al*. (ranked 3 with a score of 15.5)^35^. Other signatures in the top 10 were co-expression networks of the following genes: *CCNA2*, *HMMR*, *CCNB2*, *CDC20*, *CDC2*, *CENPF* and *RRM2*. Taken together, the top 10 enriched signatures are composed of a limited number of 92 genes strongly involved in mitosis and chromosome segregation pathways (*P* < 10^−30^; see methods). This result demonstrates that CINSARC is strongly enriched in genes associated with poor prognosis at pan-cancer level, with a high sensitivity and low false-negative rate compared to other signatures.

### Signature enrichments at cancer type level

In PRECOG, many cancer types have few outcome-associated genes (*e.g*. at *Q* < 0.05). Fifteen cancer types have no such genes and seven cancer types have from 1 to 9 such genes, for a total of 22 cancer types with less than 10 outcome-associated genes. We wondered how signature enrichments perform in cancer types with low versus high outcome-associated gene contents. We split the 39 cancer types into two subgroups using a threshold of 10 outcome-associated genes: 17 types have at least 10 such genes and 22 have less.

In 10 out of the latter 22 types (45%), CINSARC (both full and reduced) was significantly enriched in top-ranked genes (though these genes are not significantly associated with poor prognosis), the widest type coverage reached by all tested signatures (Supplementary Table 1: sheets 2 to 23). Two other cancer signatures performed similar to CINSARC: *BREAST REYAL08 72GENES* and *ROSTY CERVICAL CANCER PROLIFERATION CLUSTER*. Furthermore, eight co-expression networks in MSigDB performed similar to CINSARC: *CCNA2*, *CCNB2*, *CDC20*, *CENPF*, *MCM4*, *PCNA*, *RRM1* and *RRM2*. These 12 signatures are predominantly enriched in the same tumor types: adrenocortical cancer, medulloblastoma, gastric cancer, Burkitt’s lymphoma, liver cancer, primary liver cancer, melanoma, mesothelioma, pancreatic cancer and Ewing’s sarcoma. The lack of prognosis-associated genes in these 22 types is also measured by different indicators. They present less enriched signatures, less gene ontology enrichments and less protein-protein interactions (Wilcoxon rank sum tests are 1.71 × 10^−4^, 1.03 × 10^−2^ and 7.28 × 10^−5^, respectively; Supplementary Figure 1; see methods) compared to the 17 others (those with at least 10 outcome-associated genes).

In these 17 cancer types (with at least 10 outcome-associated genes), CINSARC (both full and reduced) was significantly enriched in top-ranked genes (significantly associated with poor prognosis) of 11 types (65%), the widest type coverage reached by all tested signatures (Supplementary Table 1: sheets 24 to 40). Several other signatures performed equally: BREAST THORNER09 217GENES, *BREAST TROESTER06 134GENES UP DOXTREATED-HME-CC*, *BREAST TROESTER06 81GENES UP SHAM HME-CC*, *CAIRO HEPATOBLASTOMA CLASSES UP*, *GSE18893 TCONV VS TREG 24H TNF STIM UP*, *NAKAYAMA SOFT TISSUE TUMORS PCA2 UP*, *RODRIGUES THYROID CARCINOMA POORLY DIFFERENTIATED UP*, STEMCELL SHATS10 100GENES CONSENSUS STEMNESS RANKING, *WHITEFORD PEDIATRIC CANCER MARKERS* and *ZHOU CELL CYCLE GENES IN IR RESPONSE 24HR*. These 12 signatures are predominantly enriched in the same tumor types: bladder cancer, astrocytoma, glioma, neuroblastoma, breast cancer, germ cell tumor, Mantle cell lymphoma, multiple myeloma, lung adenocarcinoma, ovarian cancer and prostate cancer.

Considering the full set of 39 tumor types, CINSARC covers the largest possible spectrum of tested cancer types, being enriched in top-ranked genes of 21 types: adrenocortical cancer, medulloblastoma, gastric cancer, Burkitt’s lymphoma, liver cancer, primary liver cancer, melanoma, mesothelioma, pancreatic cancer, Ewing’s sarcoma, bladder cancer, astrocytoma, glioma, neuroblastoma, breast cancer, germ cell tumor, Mantle cell lymphoma, multiple myeloma, lung adenocarcinoma, ovarian cancer and prostate cancer. Aggregated into groups (brain, blood, head and neck and solid), no significant association was observed between CINSARC enrichments and cancer families. Other 18 types, without CINSARC enrichments, are: glioblastoma, colon cancer, acute myeloid leukemia, chronic lymphoid leukemia, diffuse large B-cell lymphoma, melanoma metastasis, meningioma, head and neck cancer, hypopharyngeal cancer, oesophageal cancer, oral squamous cell carcinoma, B-cell acute lymphoblastic leukemia, follicular lymphoma, kidney cancer, large cell carcinoma of the lung, squamous cell carcinoma of the lung, small cell lung cancer and osteosarcoma. As a consequence of these results, we pursued experiments by focusing on the CINSARC signature.

### CINSARC prognostic value with survival analysis

Being enriched in genes associated with poor outcome does not essentially make a signature a prognostic factor. This can be explained by the fact that collective information of each individual gene prognostic value may be different from the prognostic value given by a whole set of genes. Accordingly, we investigated whether CINSARC was associated with survival differences in studies used to build the PRECOG resource. To obtain a robust evaluation, we filtered out datasets with less than 50 annotated cases, less than 25 CINSARC genes and <10% or >90% death rates. Accordingly, 83 datasets (out of 166; 50%) were investigated using the Kaplan-Meier estimator, covering 27 different cancer types. Since the investigated datasets did not permit generation of training-validation cohorts (median number of samples was 111), we performed leave-one-out cross-validation as the classifier method.

A total of 33 datasets (40%) produced significant survival differences according to the CINSARC classification (Supplementary Table 2, Supplementary Figures 2 and 3). The 17 cancer types associated with these 33 datasets are: neuroblastoma, breast cancer, Mantle cell lymphoma, diffuse large B-cell lymphoma, ovarian cancer, prostate cancer, acute myeloid leukemia, lung adenocarcinoma, bladder cancer, liver cancer, multiple myeloma, follicular lymphoma, colon cancer, squamous cell carcinoma of the lung, oral squamous cell carcinoma, chronic lymphoid leukemia and B-cell acute lymphoblastic leukemia. Importantly, cancer types where CINSARC was significantly enriched in top-ranked genes demonstrated higher proportion of significant survival differences (22 among 42 datasets; 52%) compared to the others (11 among 41; 27%). Also, cancer types with at least 10 genes associated with poor prognosis demonstrated higher proportion of significant survival differences (25 among 56 datasets; 45%) compared to the others (8 among 27; 30%).

Though no correlation has been measured between survival difference and microarray platform or the number of evaluated CINSARC genes, clear evidence of cohort size effect has been observed: the larger cohort, the higher survival difference (Wilcoxon rank sum test *P* = 1.7e-5). Among the 83 tested datasets, 46 contain at least 100 tumors and CINSARC classification is significantly associated with the outcome in 26 of them (57%). There are 30 datasets with at least 150 tumors and CINSARC classification is significantly associated with the outcome in 19 of them (63%). At 200 tumors threshold, CINSARC classification is significantly associated with the outcome in 11 out of 16 (69%) datasets.

In these 11 datasets, we wondered how would perform CINSARC if less tumors were taken into account. In fact, we previously reported CINSARC robustness as it was a clinical marker in independent sarcoma datasets^29^ but we did not evaluate its robustness in term of cohort subsampling^36^. To firmly evaluate CINSARC robustness regarding cohort size, we investigated the 11 datasets with a least 200 tumors where CINSARC is a prognostic factor. For each dataset, we randomly generated 10,000 sub-cohorts at 75% and 50% of the total cohort size with similar death rates (±10%; Supplementary Figure 4). All of the 11 datasets demonstrate that better prognoses are obtained with 75% compared to 50% subsampling. In eight datasets, CINSARC still classify tumors according to their aggressiveness in most of random trials (>50% at *P* < 0.05). However, due to the decrease in statistical power, CINSARC mostly produces non-significant prognoses in three datasets (PMID: 17410195, 21720365 and 17023574, cancer types are: B-cell acute lymphoblastic leukemia, multiple myeloma and ovarian tumors, respectively). These results confirm that cohort sizes affect prognosis values and that, nonetheless, CINSARC is a robust signature as it remains a prognostic marker for tumor aggressiveness in subsampling analyses.

## Discussion

Cancer gene expression signatures have been widely used as tools to classify tumors into specific subtypes, as a prognostic factor for clinical outcomes or as a predictive factor for treatment responses^37^. Using the GSEA algorithm, we demonstrated that CINSARC, among 15,499 signatures established by various methods, is strongly enriched in prognostic genes at pan-cancer level, with high sensitivity (>95%) and low false-negative rate (<3%). At tumor-specific level, CINSARC covers the largest possible spectrum of tumor types among all tested cancer gene expression signatures (enriched in 21 cancers types among 39 tested). As it was not evaluated in PRECOG, we hypothesize that *BORA* gene would increase CINSARC prognostic value since it has been described as an important Aurora kinase A (*AURKA*) activator, required for centrosome maturation, spindle assembly and asymmetric protein localization during mitosis^38^. Moreover, *AURKA* alone has been identified as a prognostic marker in gastrointestinal stromal tumors^26^. Originally, CINSARC is a metastatic marker to predict which sarcomas with complex genetics have high relapse risk (on average 50% at 5-year)^24^. The results we present demonstrate its usefulness as a generic tumor aggressiveness predictor since overall/disease-specific survivals were considered in PRECOG.

Importantly, several PRECOG factors could restrict signature prognostic values, CINSARC included, in the different cancer types. First: it is questionable that a single tumor dataset represents the classical cancer-specific transcriptomic profile and therefore another dataset could produce different results. Second: some studies comprise very high or very low (typically > 90% or < 10%, respectively) death rates which could be a limiting factor in PRECOG survival evaluation since cohorts are split by median gene expression. Third: several cancer types, due to microarray models used in the different studies, may lack important genes so prognostic values were limited to a restricted number of genes (*i.e*. GPL80: Affymetrix Hu6800, GPL91: Affymetrix HG-U95A, non-commercial: GPL257 and GPL3906, etc.). Fourth: it has been described that cohort size is a parameter affecting survival estimation^39^. To bypass these limitations and confirm GSEA results, we performed survival analyses using CINSARC classification. Most datasets (>57%) with at least 100 samples presented a significantly survival difference whereas smaller datasets performed worst. This can be explained as statistical power depends on sample size so the larger dataset, the more statistical power. Moreover, the classification method (leave-one-out cross-validation) computes more robust gene expression patterns with large datasets. Finally, we assessed CINSARC robustness in terms of cohort subsampling and, previously reported, dataset independence^29^.

Of note, 483 signatures (out of 15,499; 3%) were investigated at pan-cancer level with unexpected small intersections (<90%) with known genes in PRECOG, corresponding to 10,004 unique genes not reported in PRECOG. A large proportion is punctual: 92% of them only appear in maximum two signatures. As for the recurring genes (806), majority are uncharacterized and/or non-coding genes: 414 are genes predicted by open reading frame analyses, 92 are genes with unknown functions and unidentified orthologs, 60 are L and R ribosomal protein pseudogenes and 28 are long intergenic non-protein coding RNA. Considering the recurrent and curated genes, only 212 of them were not evaluated in PRECOG resource, suggesting high coverage of annotated genes.

We thus demonstrated that CINSARC is enriched in genes having a significant impact on tumor aggressiveness and that this signature can be used as a prognostic marker in multiple malignancies. This highlights the point that mitosis and chromosome segregations are two key pathways leading tumor aggressiveness through genomic instability, a well-known hallmark mechanism^40^. Part of the CINSARC signature, it has been observed that overexpression of several individual genes disrupts chromosomal segregation, generating genomic alterations and tumors in mice (*MAD2L2*, *BUB1*, *CCNB1*, *CCNB2* and *ESPL1*)^41–44^. A recent study highlighted an aberrant persistence of several CINSARC proteins beyond mitosis in tetraploid versus diploid cells from sarcoma cell lines^45^. This was correlated to high motility (migration and invasion) capabilities measured in tetraploid versus diploid cells, whereas proliferation levels were similar. Though clear evidence of anaphase-promoting complex (APC) deficiency has to be established, a question arises about the impact of this protein persistence and the clinical prognostic interest of this signature.

Recently, we granted CINSARC a better clinical applicability with two major improvements^46^. First: the ability to perform RNA-seq rather than microarray technique, overcoming probe selection as probes can be transcript-specific and could not reflect full gene expression. Second: the ability to analyze FFPE (Formalin-Fixed, Paraffin-Embedded) tumor, daily material used by pathologists as opposed to frozen tissues previously required. As a consequence, two ongoing French and Europe-wide clinical trials will prospectively test the CINSARC signature in various sarcomas. Our results increase its application scope, demonstrating that this signature can be considered as a poor outcome predictor in multiple human malignancies. Finally, though it remains a very informative resource, we highlighted some limitations of PRECOG. Consequently, this study incites additional validations on other independent cancer-specific datasets.

## Methods

### Gene Set Enrichment Analysis (GSEA) usage

To evaluate the prognostic value of different gene sets as the collective information of individual genes, we used the *GSEAPreranked* tool from GSEA (Gene Set Enrichment Analysis; v2.2.3) algorithm^34^. The algorithm ranks genes based on the available PRECOG *z*-scores, which represents the significance of the association of a given gene to the prognosis of a given cancer type. All combined cancer types were summarized into meta-*z*-scores also available in PRECOG. Based on gene ranks, the algorithm computes a running-sum statistics to determine the enrichment score of each gene set. The enrichment scores are then normalized to take into account different gene set sizes, giving the normalized enrichment score (NES), and significance levels were determined with the family-wise error rate, a more conservative method than the false-discovery rate. Signatures with *P* < 0.05 (family-wise error rate) were considered significantly enriched.

Three leading-edges statistics are also computed. First: tag corresponds to the percentage of genes in a given signature hit before the maximum enrichment score is attained. By analogy, it represents the sensitivity since a high tag value indicates a higher proportion of the signature participating in the core enrichment. Second: list corresponds to the percentage of total genes before the maximum enrichment score. By analogy, it represents the false-negative rate (*e.g*. 1-specificity) since a low list value indicates a high gene set enrichment purity. Third: signal corresponds to the enrichment signal strength combining the two previous metrics. A high signal value indicates a top-ranked narrow enrichment.

### Other statistical methods

Biological pathways were determined using GOseq (v1.26.0) package from Bioconductor on the top 200 genes ranked by decreasing meta-*z*-scores or *z*-scores^47^. *P* are given by the Wallenius noncentral hypergeometric distribution method and adjusted with Benjamini-Hochberg correction for multiple comparisons.

Protein-protein interactions were determined using expected/existing interaction ratios from STRINGdb (1.14.0) package from Bioconductor with database version 10 and score threshold set to 400 (removes low-confidence interactions) on the top 200 genes ranked by decreasing meta-*z*-scores or *z*-scores. The lower expected/existing interaction ratios, the higher are the number of protein-protein interactions^48^.

The leave-one-out cross-validation classification was performed as follows: each sample was categorized using the nearest centroid method according to outcomes and transcriptomic profiles of all other samples.

Survival analyses were measured using survival (v2.40-1) package with the Kaplan-Meier estimator, where significance levels are given by the log-rank test (*P*) and survival differences given by hazard ratios (HR).

Miscellaneous computations, filters, statistics and plots were performed with R (3.3.2).

## Electronic supplementary material

Supplementary information

Supplementary table 1
