# Supplementary material for: The CINSARC signature as a prognostic marker for clinical outcome in multiple neoplasms
Source: Sci Rep. 2017 Jul 14;7:5480. doi: 10.1038/s41598-017-05726-x (PMC5511191; doi:10.1038/s41598-017-05726-x)
Supplement: Supplementary file 1 — Supplementary information [file 41598_2017_5726_MOESM1_ESM.pdf]

The CINSARC signature as a prognostic marker for clinical outcome in multiple neoplasms

Tom Lesluyes
Lucile Delespaul
Jean−Michel Coindre
Frédéric Chibon

Supplementary information

Supplementary legends

Supplementary table 1: Significantly enriched signatures (family-wise error rate $P<0.05$) at pan-cancer level (sheet 1) and for each tumor type (sheets 2 to 40), sorted by mean ranks order. NES refers to Normalized Enrichment Score.

Supplementary table 2: Survival analyses on 83 published studies, ranked by significance levels. PMID refers to PubMed Identifier. *P* refers to nominal log-rank test significance. HR refers to Hazard Ratio. HR CI refers to Hazard Ratio Confidence Interval.

Supplementary figure 1: Differences between low and high (dark grey and light grey, respectively) outcome-associated gene contents in the different cancer types. Tested metrics are (from left to right): the number of significantly enriched signatures, the number of enriched gene ontologies and the number of protein-protein interactions (measured by expected/existing ratios; the lower ratio, the higher interactions).

Supplementary figure 2: Overview of survival information in the 33 published studies with significant survival differences according to the CINSARC signature. Low and high risk-groups are represented in blue and red colors, respectively. Complete details of these survival analyses can be found in supplementary figure 3. *P* refers to nominal log-rank test significance. HR refers to Hazard Ratio.

Supplementary figure 3: Complete survival information in the 33 published studies with significant survival differences according to the CINSARC signature. Survival table, below every Kaplan-Meier, describes survival information at several times: from 0 to 5 years. *P* refers to nominal log-rank test significance. HR refers to Hazard Ratio. C1 and C2 refer to the low and high-risk CINSARC classifications, respectively. N risk refers to the remaining population size.

Supplementary figure 4: Evaluation of cohort size effect on prognosis value. Compared to the significance level of CINSARC in the full cohort (P_{Ref}) and to the standard significance threshold (P=0.05; vertical dashed lines), significance levels for 75% and 50% subsampling are given by the red and blue densities, respectively. Distributions of prognoses compared to 0.05 and P_{Ref} are indicated in the table below each density plot. $P>0.05$ represent non-significant subsampling, $P_{Ref}<P<0.05$ represent significant subsampling with lower prognosis than the complete cohort and $P<P_{Ref}$ represent better prognosis than the complete cohort. The sum of the two latter categories represents significant subsampling.

Supplementary figure 1

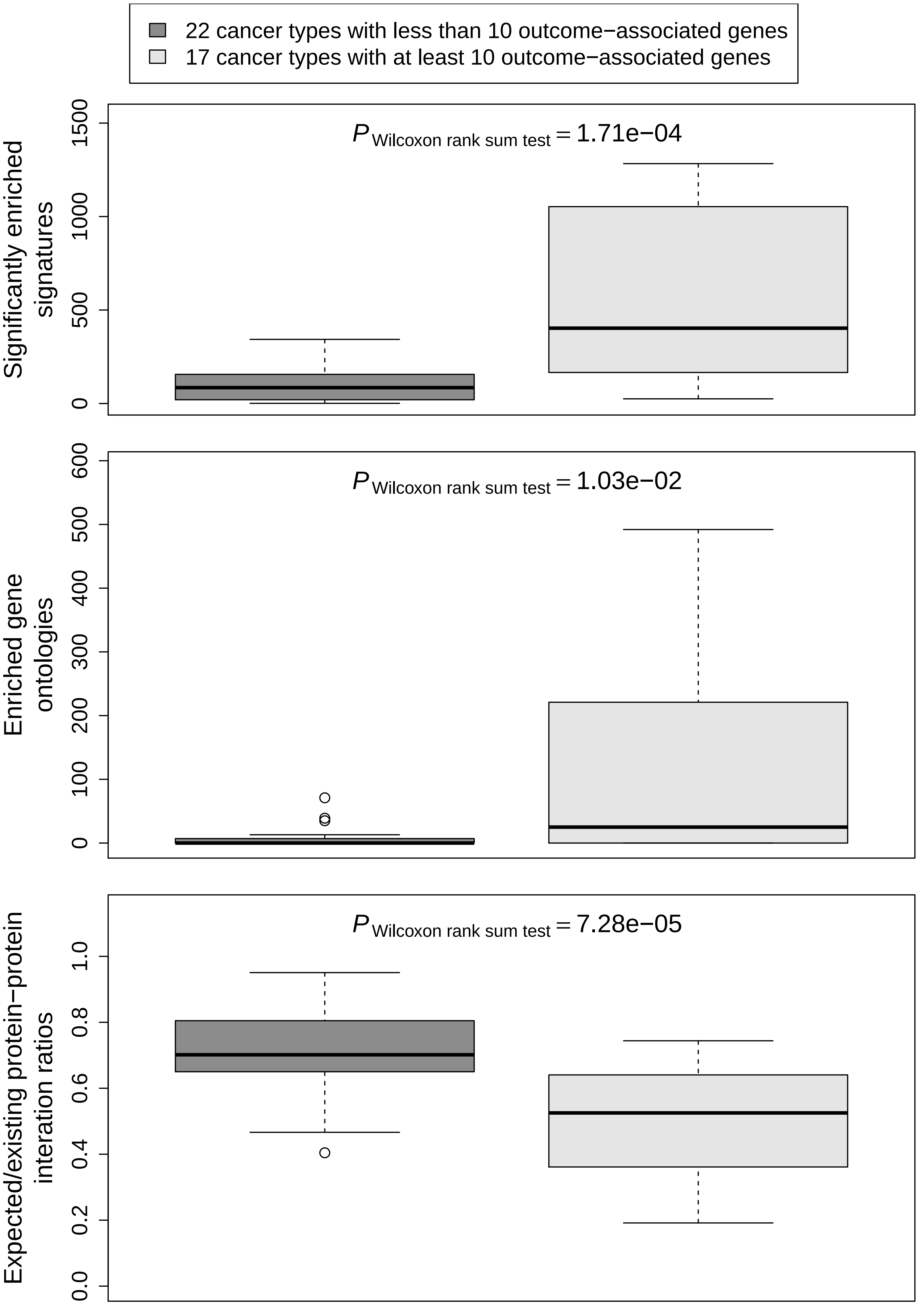

22 cancer types with less than 10 outcome−associated genes
17 cancer types with at least 10 outcome−associated genes
Significantly enriched signatures
P Wilcoxon rank sum test = 1.71e−04
0
500
1000
1500
Enriched gene ontologies
P Wilcoxon rank sum test = 1.03e−02
0
100
200
300
400
500
600
Expected/existing protein−protein interation ratios
P Wilcoxon rank sum test = 7.28e−05
0.0
0.2
0.4
0.6
0.8
1.0

Supplementary figure 2

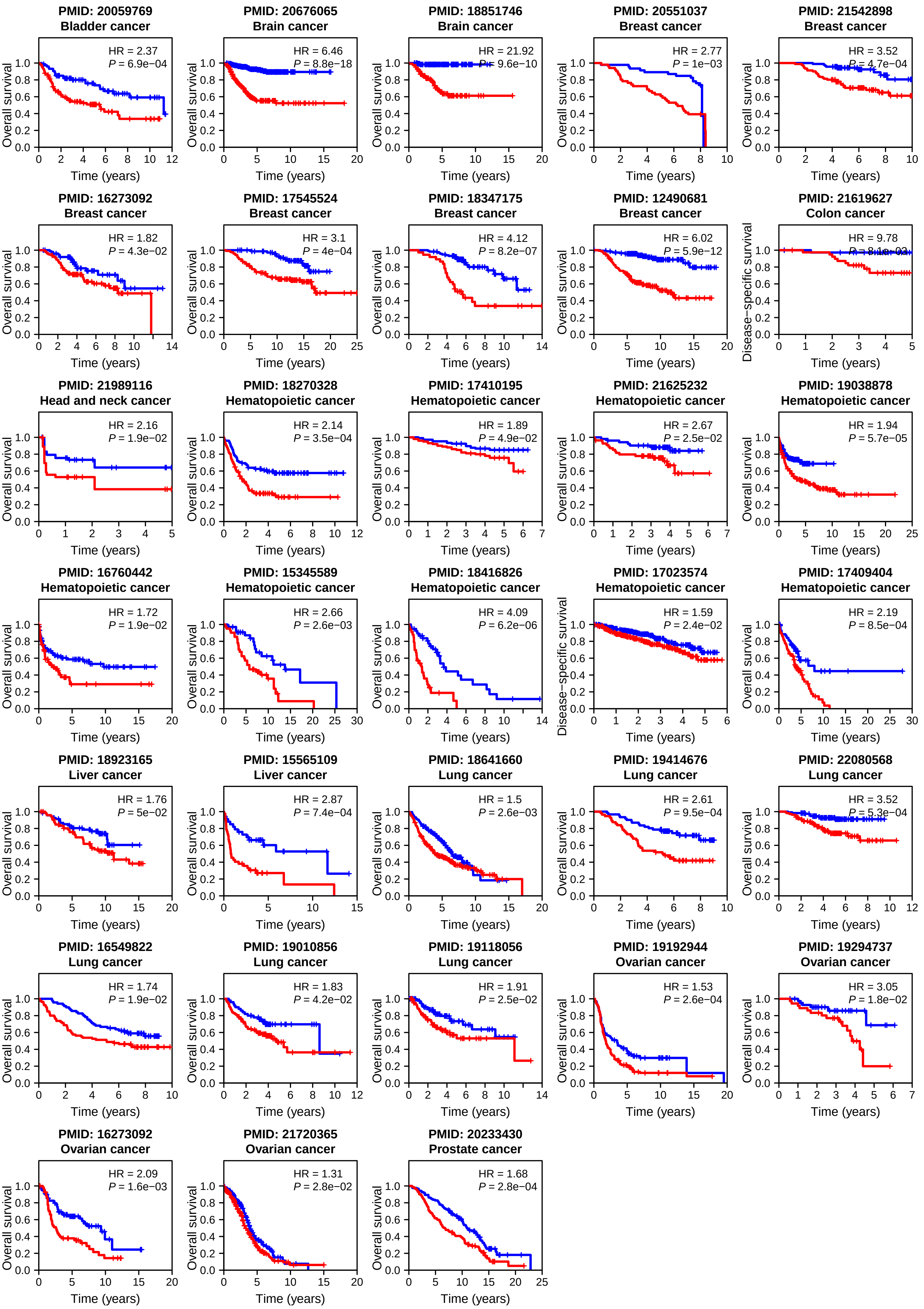

PMID: 20059769
Bladder cancer
HR = 2.37
P = 6.9e−04
PMID: 20676065
Brain cancer
HR = 6.46
P = 8.8e−18
PMID: 18851746
Brain cancer
HR = 21.92
P = 9.6e−10
PMID: 20551037
Breast cancer
HR = 2.77
P = 1e−03
PMID: 21542898
Breast cancer
HR = 3.52
P = 4.7e−04
PMID: 16273092
Breast cancer
HR = 1.82
P = 4.3e−02
PMID: 17545524
Breast cancer
HR = 3.1
P = 4e−04
PMID: 18347175
Breast cancer
HR = 4.12
P = 8.2e−07
PMID: 12490681
Breast cancer
HR = 6.02
P = 5.9e−12
PMID: 21619627
Colon cancer
HR = 9.78
Disease−specific survival
PMID: 21989116
Head and neck cancer
HR = 2.16
P = 1.9e−02
PMID: 18270328
Hematopoietic cancer
HR = 2.14
P = 3.5e−04
PMID: 17410195
Hematopoietic cancer
HR = 1.89
P = 4.9e−02
PMID: 21625232
Hematopoietic cancer
HR = 2.67
P = 2.5e−02
PMID: 19038878
Hematopoietic cancer
HR = 1.94
P = 5.7e−05
PMID: 16760442
Hematopoietic cancer
HR = 1.72
P = 1.9e−02
PMID: 15345589
Hematopoietic cancer
HR = 2.66
P = 2.6e−03
PMID: 18416826
Hematopoietic cancer
HR = 4.09
P = 6.2e−06
PMID: 17023574
Hematopoietic cancer
HR = 1.59
P = 2.4e−02
Disease−specific survival
PMID: 17409404
Hematopoietic cancer
HR = 2.19
P = 8.5e−04
PMID: 18923165
Liver cancer
HR = 1.76
P = 5e−02
PMID: 15565109
Liver cancer
HR = 2.87
P = 7.4e−04
PMID: 18641660
Lung cancer
HR = 1.5
P = 2.6e−03
PMID: 19414676
Lung cancer
HR = 2.61
P = 9.5e−04
PMID: 22080568
Lung cancer
HR = 3.52
P = 5.3e−04
PMID: 16549822
Lung cancer
HR = 1.74
P = 1.9e−02
PMID: 19010856
Lung cancer
HR = 1.83
P = 4.2e−02
PMID: 19118056
Lung cancer
HR = 1.91
P = 2.5e−02
PMID: 19192944
Ovarian cancer
HR = 1.53
P = 2.6e−04
PMID: 19294737
Ovarian cancer
HR = 3.05
P = 1.8e−02
PMID: 16273092
Ovarian cancer
HR = 2.09
P = 1.6e−03
PMID: 21720365
Ovarian cancer
HR = 1.31
P = 2.8e−02
PMID: 20233430
Prostate cancer
HR = 1.68
P = 2.8e−04
Overall survival
Time (years)

Supplementary figure 3

PMID: 20059769 − Bladder cancer
CINSARC coverage: 65 genes (97.01%)

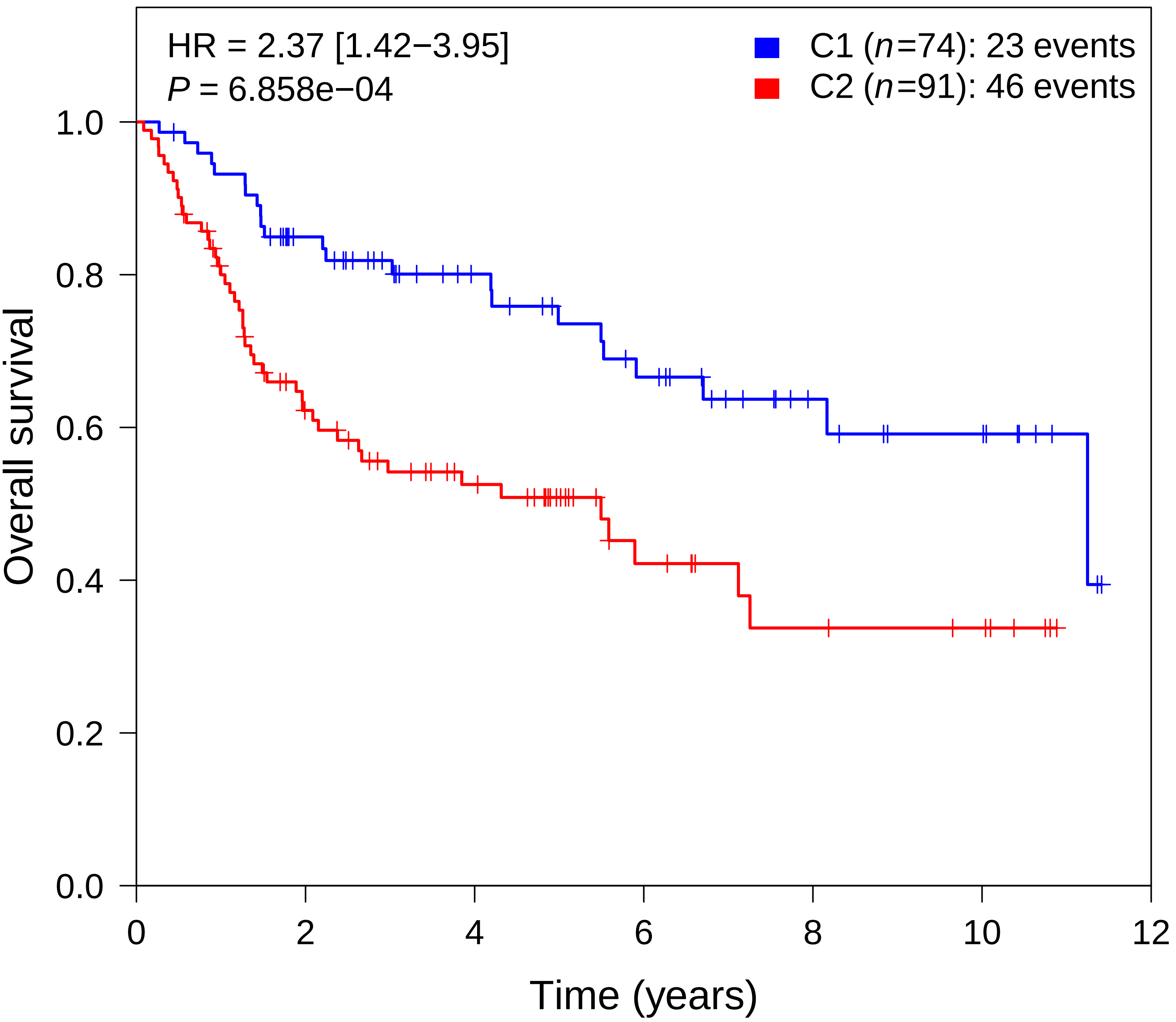

Group	Metrics	Time: 0	Time: 1	Time: 2	Time: 3	Time: 4	Time: 5
C1	N risk	74	68	55	46	38	32
	Events	0	5	11	13	14	17
	Survival	1	0.9317	0.8495	0.8186	0.8008	0.7357
C2	N risk	91	69	48	38	32	23
	Events	0	18	33	39	40	41
	Survival	1	0.7999	0.6223	0.5417	0.5253	0.5083

PMID: 20676065 − Brain cancer (Neuroblastoma)
CINSARC coverage: 67 genes (100%)

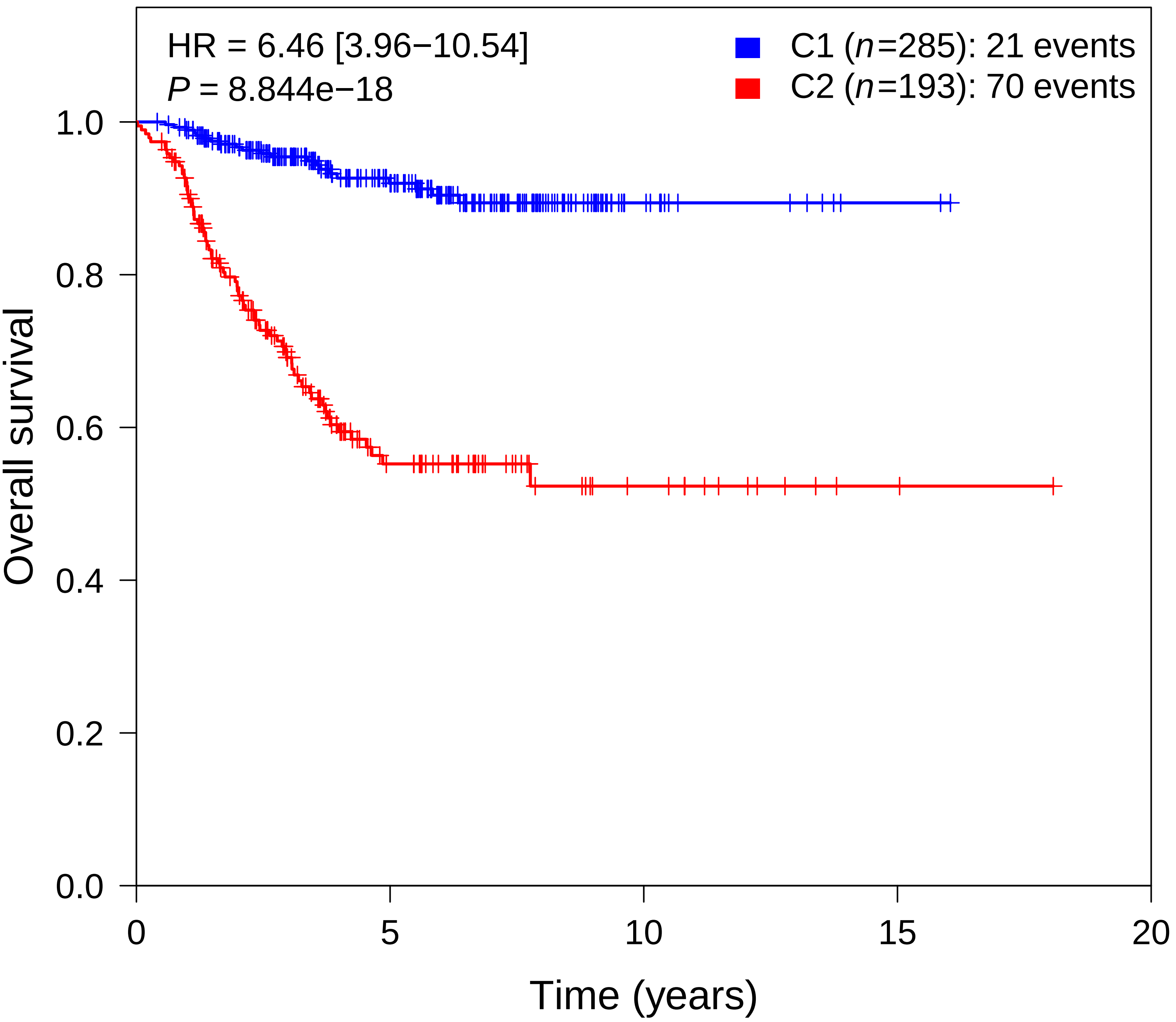

Group	Metrics	Time: 0	Time: 1	Time: 2	Time: 3	Time: 4	Time: 5
C1	N risk	285	277	243	200	158	136
	Events	0	3	9	12	17	18
	Survival	1	0.9894	0.967	0.9543	0.9264	0.9197
C2	N risk	193	169	127	92	65	49
	Events	0	17	40	53	65	69
	Survival	1	0.9105	0.7786	0.6915	0.5945	0.5522

PMID: 18851746 − Brain cancer (Neuroblastoma)
CINSARC coverage: 55 genes (82.09%)

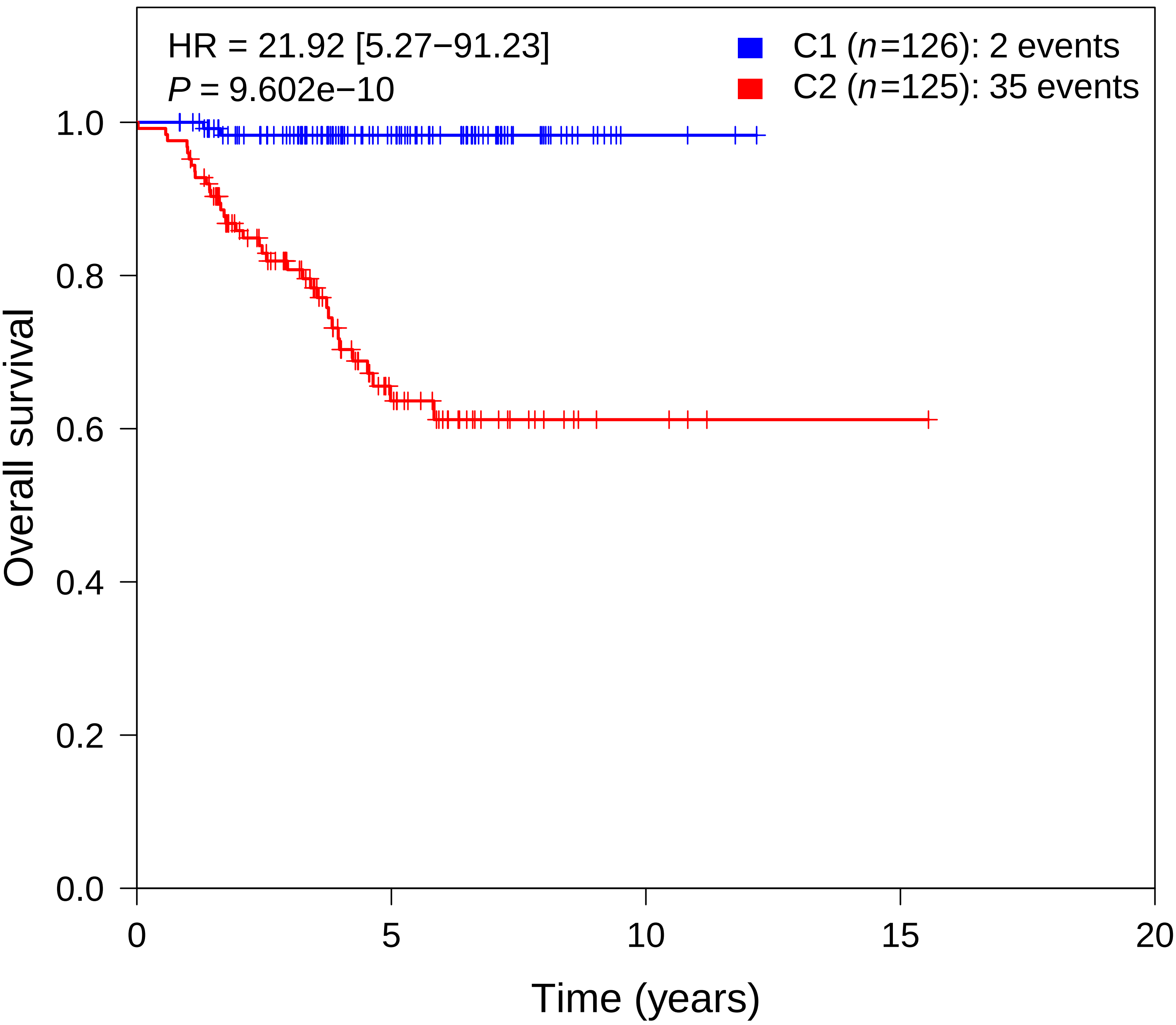

Group	Metrics	Time: 0	Time: 1	Time: 2	Time: 3	Time: 4	Time: 5
C1	N risk	126	124	109	100	76	61
	Events	0	0	2	2	2	2
	Survival	1	1	0.9831	0.9831	0.9831	0.9831
C2	N risk	125	120	91	71	50	33
	Events	0	5	17	22	30	34
	Survival	1	0.96	0.8585	0.8075	0.7033	0.6363

PMID: 20551037 − Breast cancer
CINSARC coverage: 67 genes (100%)

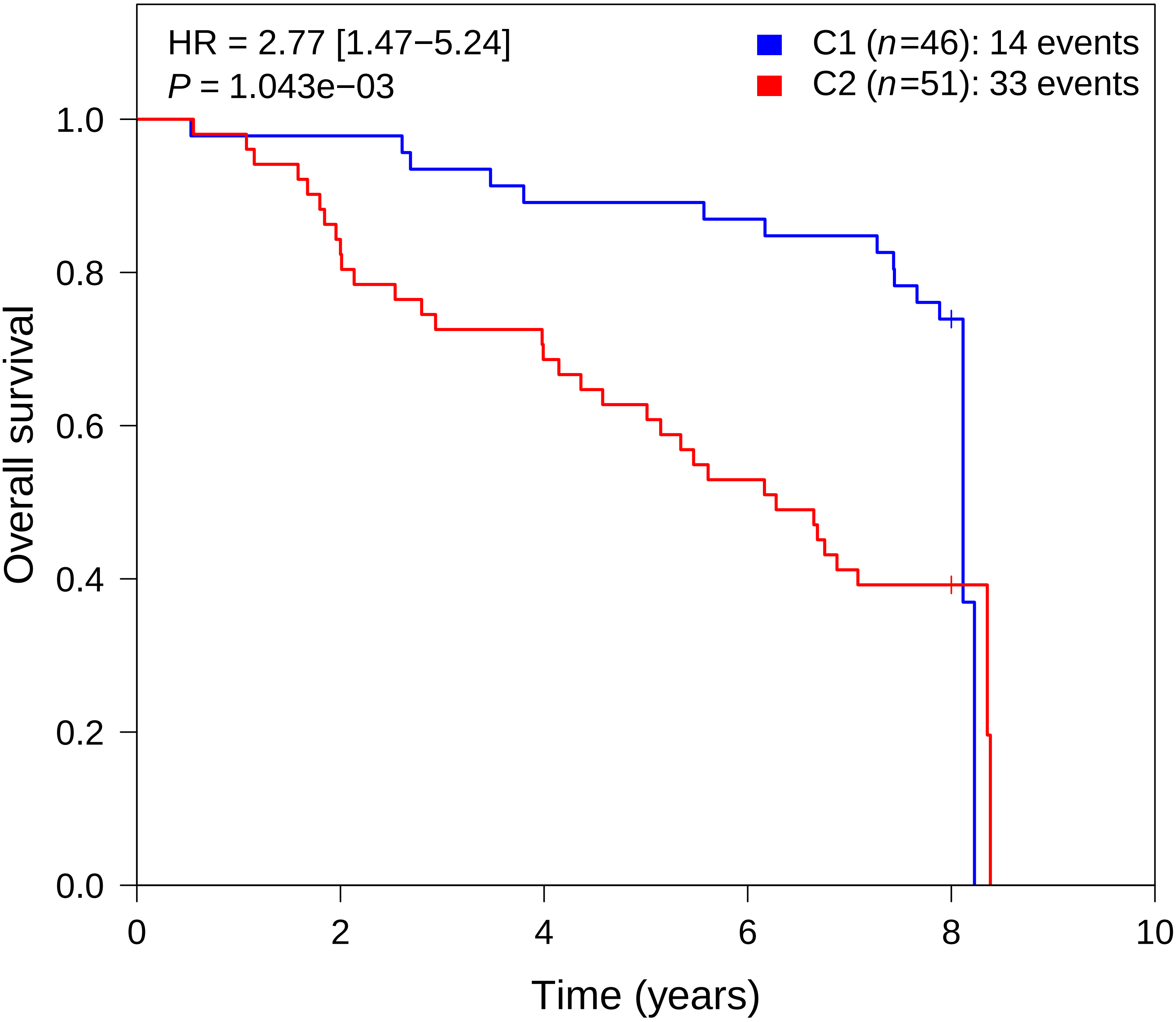

Group	Metrics	Time: 0	Time: 1	Time: 2	Time: 3	Time: 4	Time: 5
C1	N risk	46	45	45	43	41	41
	Events	0	1	1	3	5	5
	Survival	1	0.9783	0.9783	0.9348	0.8913	0.8913
C2	N risk	51	50	43	37	35	32
	Events	0	1	9	14	16	19
	Survival	1	0.9804	0.8235	0.7255	0.6863	0.6275

PMID: 21542898 − Breast cancer
CINSARC coverage: 67 genes (100%)

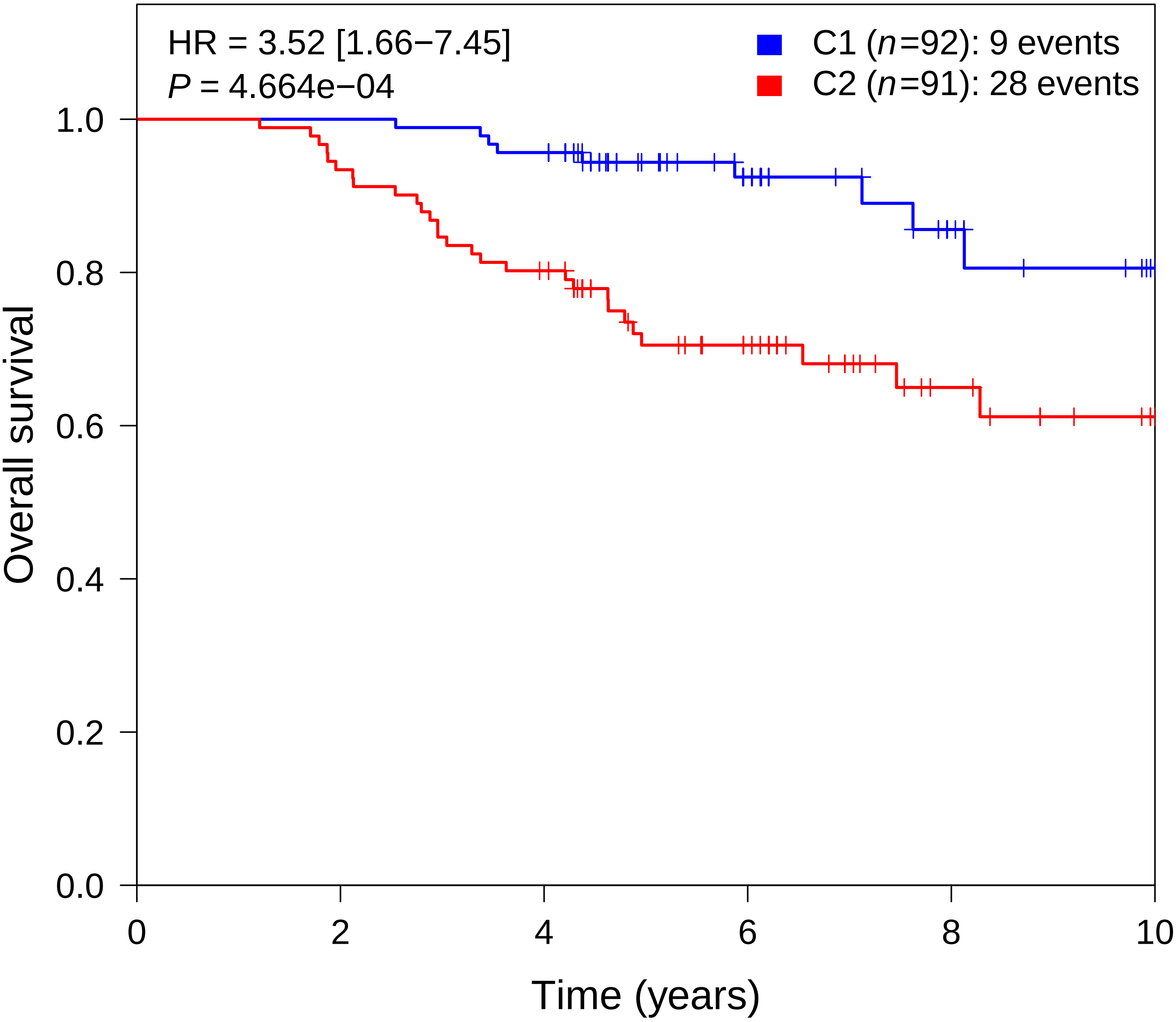

Group	Metrics	Time: 0	Time: 1	Time: 2	Time: 3	Time: 4	Time: 5
C1	N risk	92	92	92	91	88	56
	Events	0	0	0	1	4	5
	Survival	1	1	1	0.9891	0.9565	0.9438
C2	N risk	91	91	85	77	72	47
	Events	0	0	6	14	18	25
	Survival	1	1	0.9341	0.8462	0.8022	0.7051

PMID: 16273092 − Breast cancer
CINSARC coverage: 46 genes (68.66%)

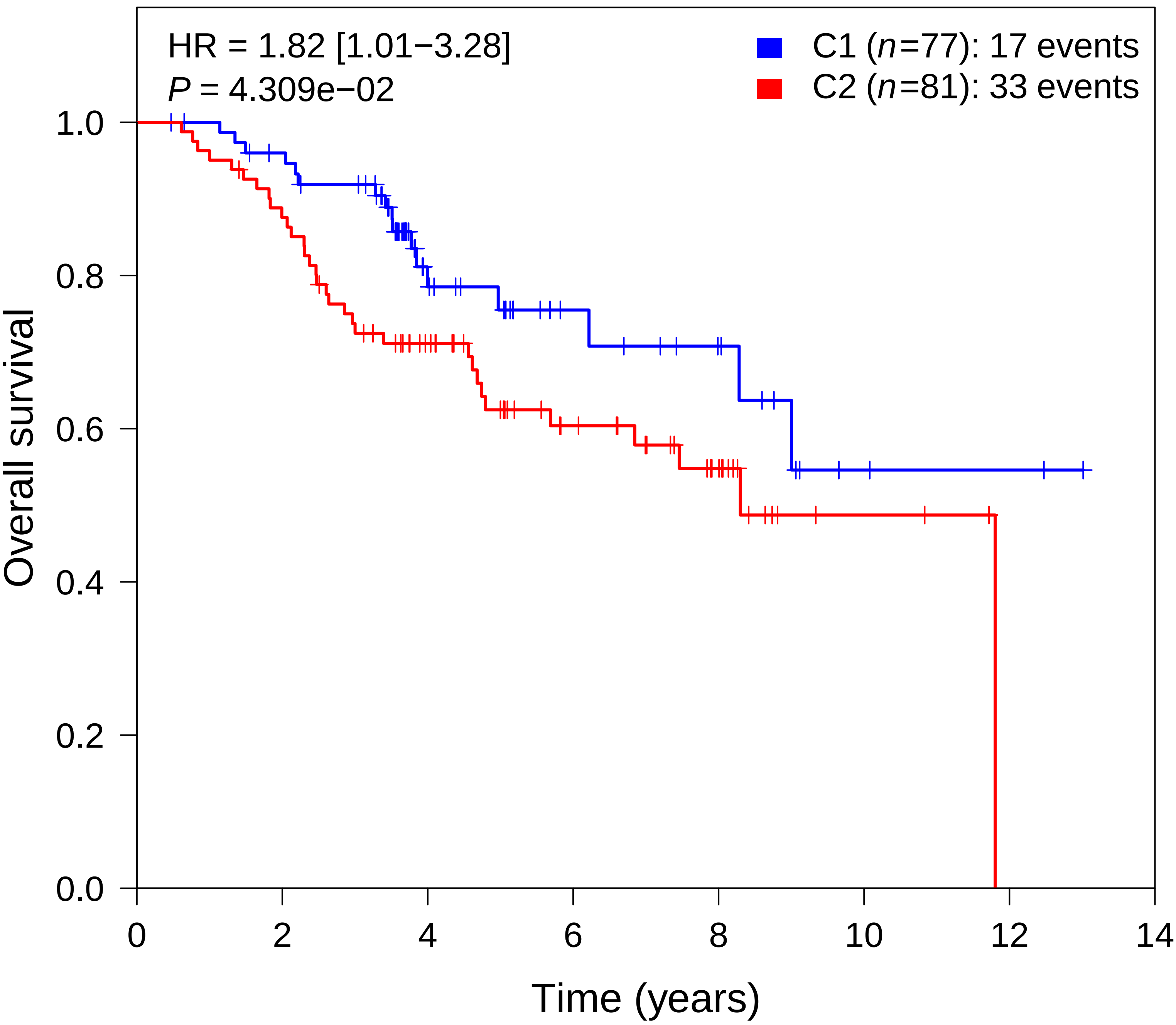

Group	Metrics	Time: 0	Time: 1	Time: 2	Time: 3	Time: 4	Time: 5
C1	N risk	77	75	70	66	30	25
	Events	0	0	3	6	13	14
	Survival	1	1	0.96	0.9189	0.7852	0.755
C2	N risk	81	77	70	58	47	35
	Events	0	4	10	21	23	28
	Survival	1	0.9506	0.8757	0.7373	0.7114	0.6247

PMID: 17545524 − Breast cancer
CINSARC coverage: 62 genes (92.54%)

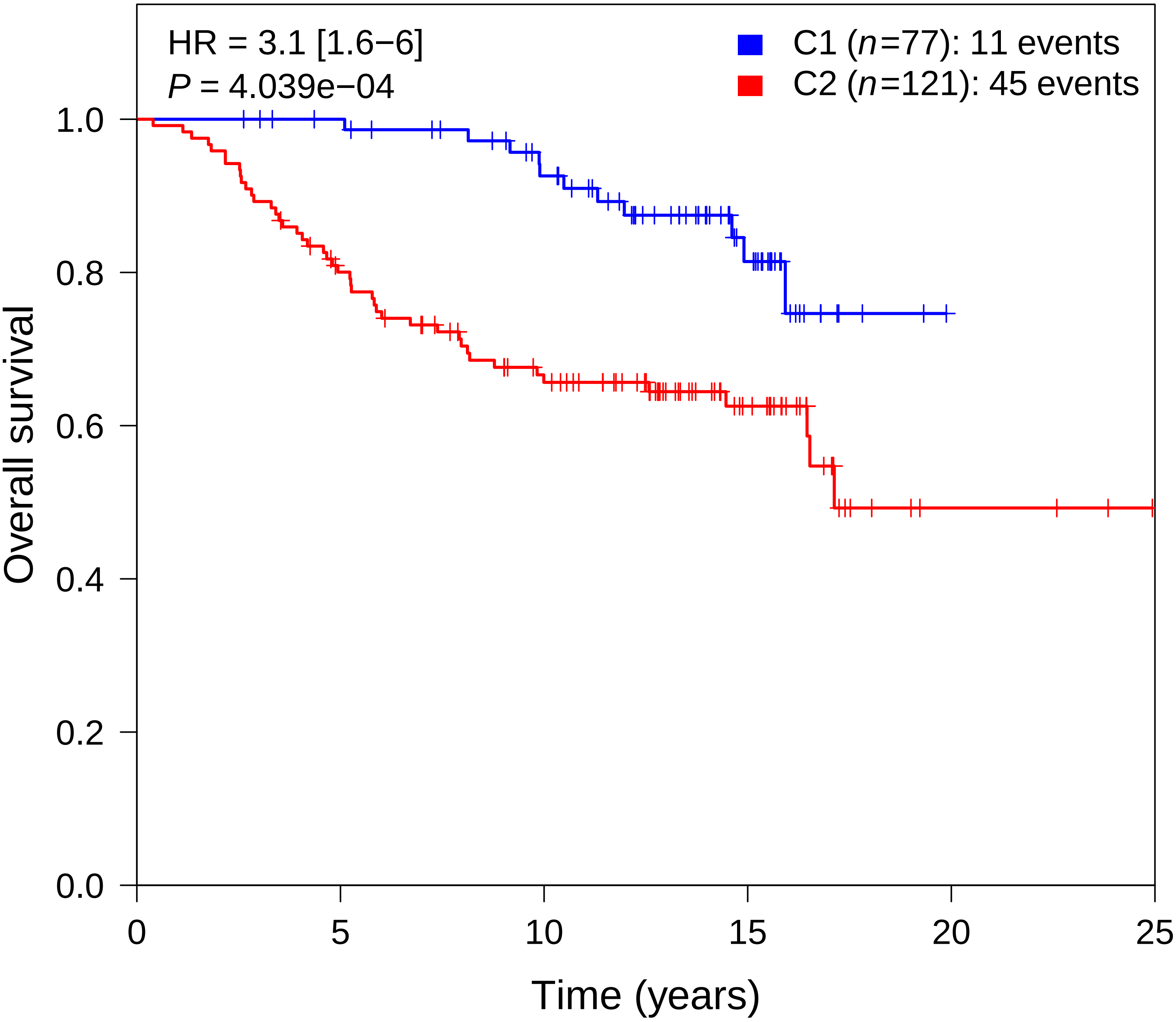

Group	Metrics	Time: 0	Time: 1	Time: 2	Time: 3	Time: 4	Time: 5
C1	N risk	77	77	77	76	74	73
	Events	0	0	0	0	0	0
	Survival	1	1	1	1	1	1
C2	N risk	121	120	116	108	102	93
	Events	0	1	5	13	18	24
	Survival	1	0.9917	0.9587	0.8926	0.8511	0.8004

PMID: 18347175 − Breast cancer
CINSARC coverage: 32 genes (47.76%)

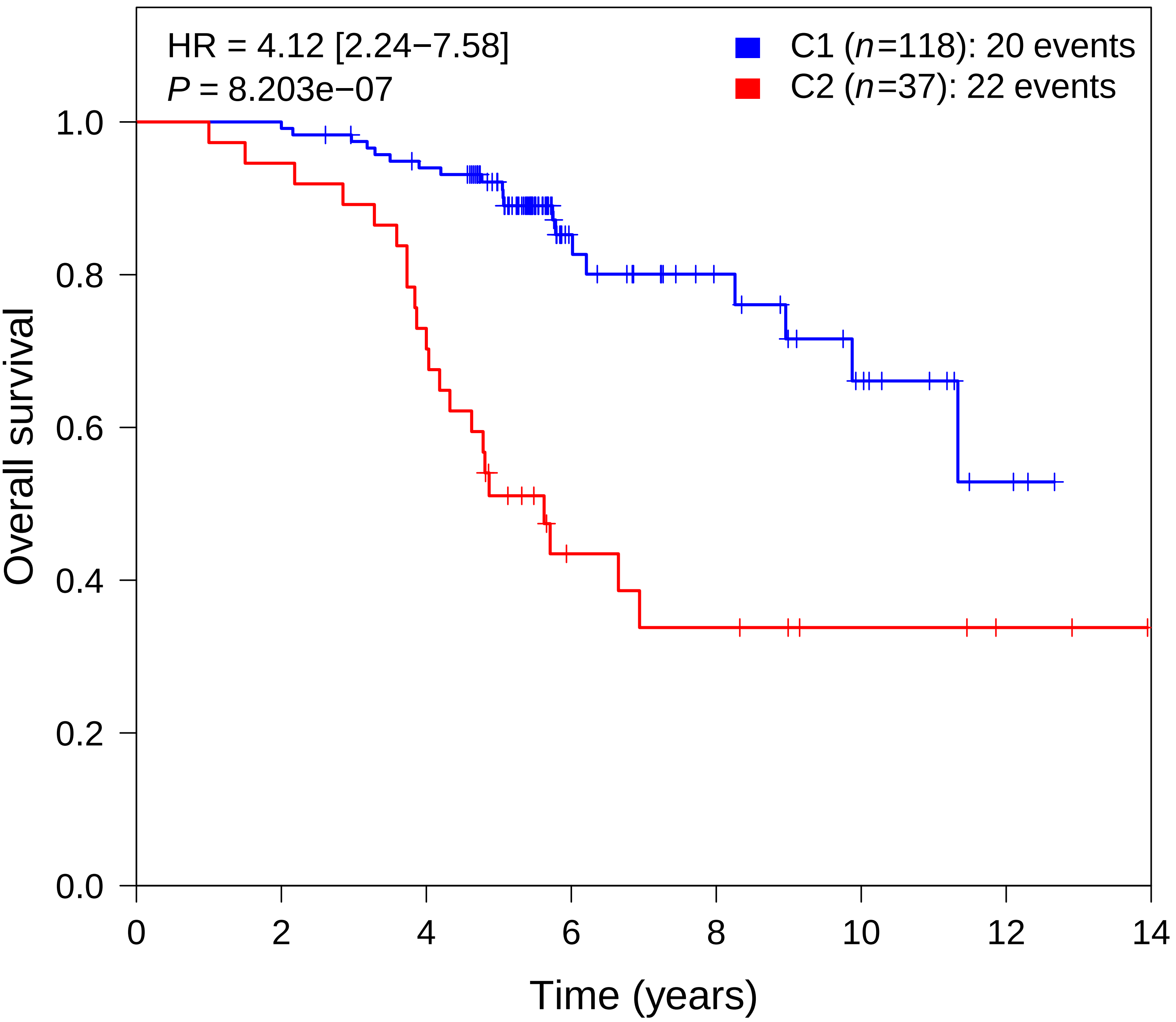

Group	Metrics	Time: 0	Time: 1	Time: 2	Time: 3	Time: 4	Time: 5
C1	N risk	118	118	118	113	108	89
	Events	0	0	1	3	7	9
	Survival	1	1	0.9915	0.9744	0.9399	0.9214
C2	N risk	37	37	35	33	27	17
	Events	0	1	2	4	11	18
	Survival	1	0.973	0.9459	0.8919	0.7027	0.5105

PMID: 12490681 − Breast cancer
CINSARC coverage: 57 genes (85.07%)

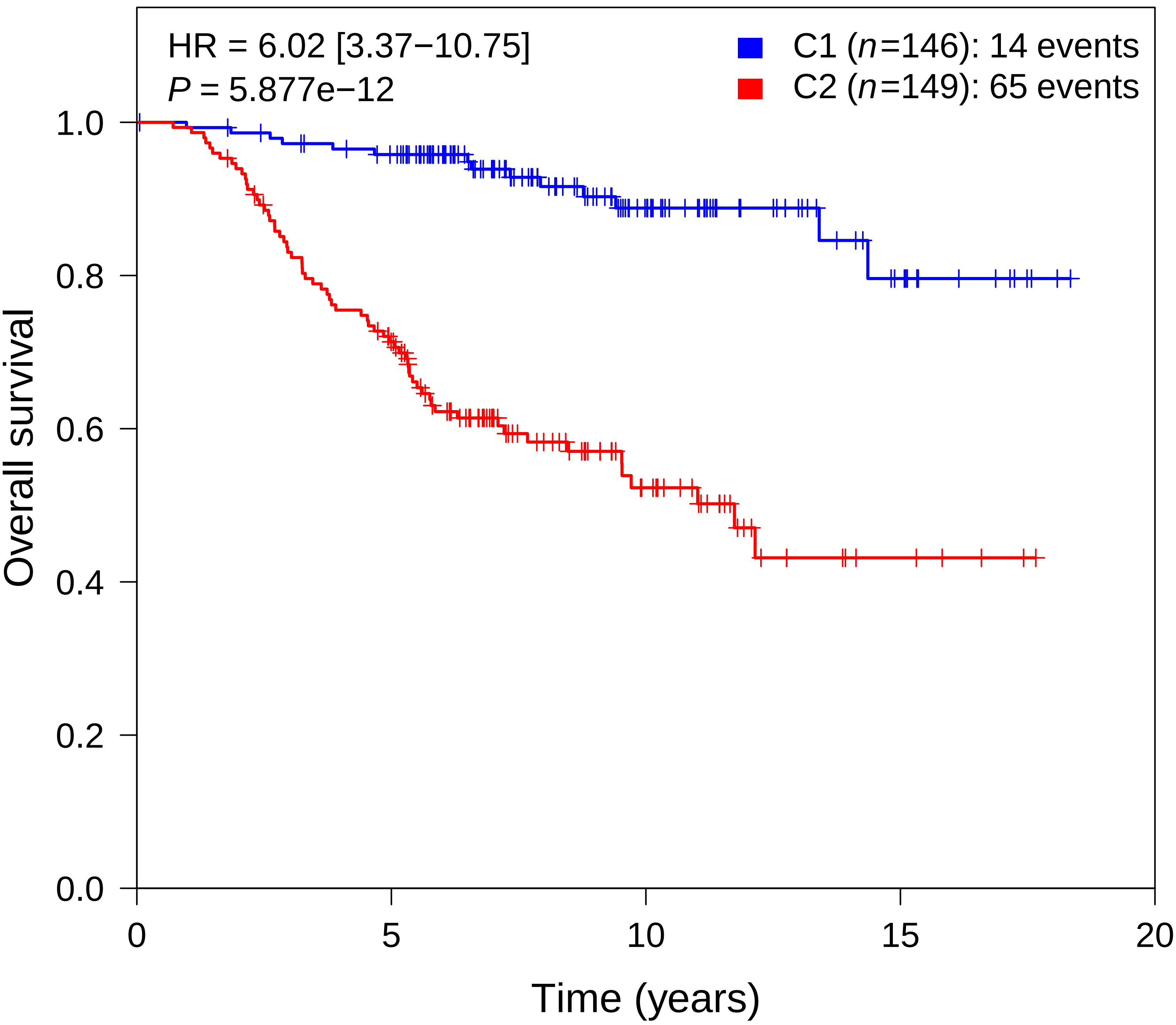

Group	Metrics	Time: 0	Time: 1	Time: 2	Time: 3	Time: 4	Time: 5
C1	N risk	146	144	142	139	136	132
	Events	0	1	2	4	5	6
	Survival	1	0.9931	0.9862	0.9722	0.9651	0.9579
C2	N risk	149	148	139	121	110	100
	Events	0	1	9	25	36	42
	Survival	1	0.9933	0.9395	0.8303	0.7548	0.7134

PMID: 21619627 − Colon cancer
CINSARC coverage: 67 genes (100%)

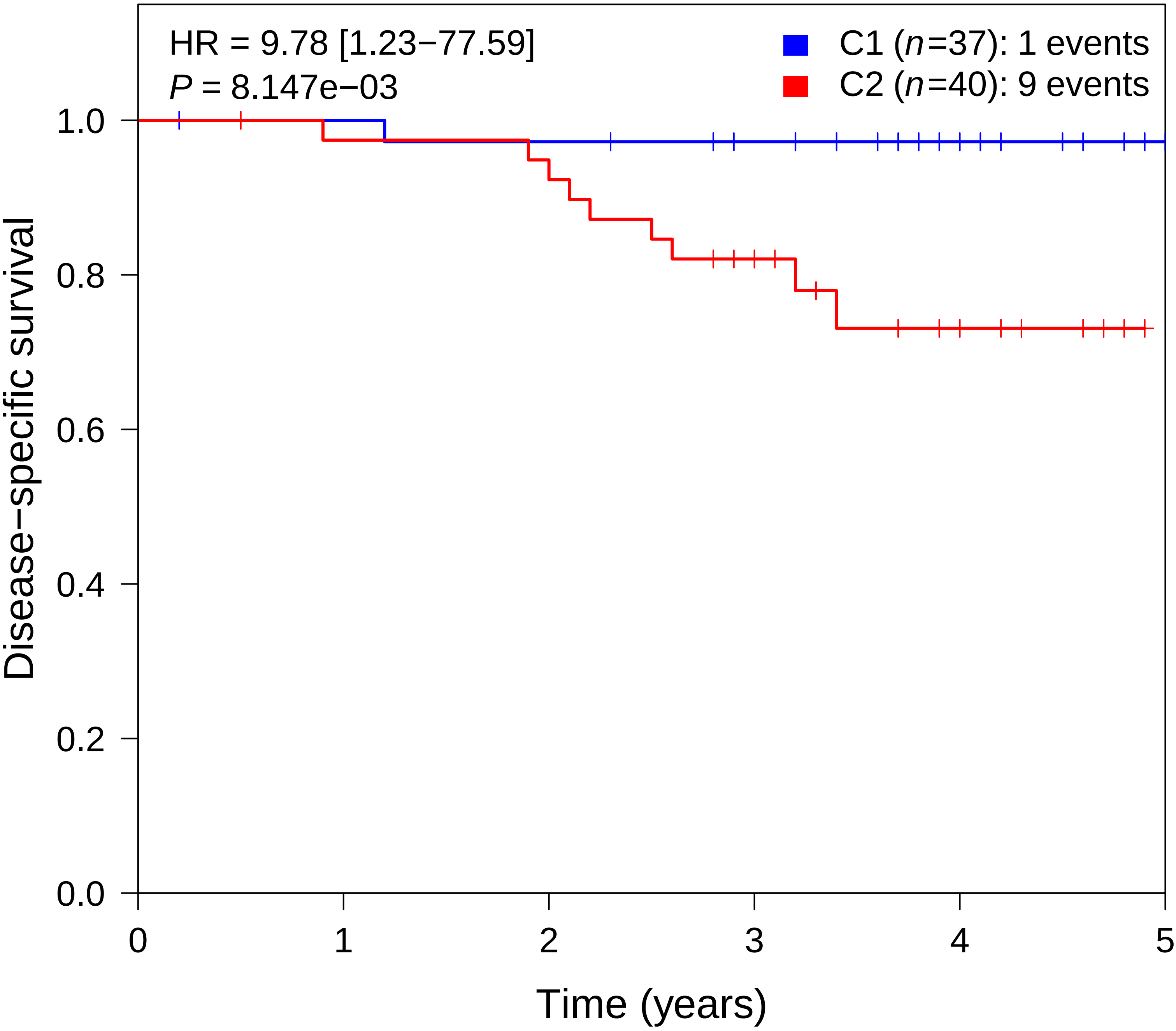

Group	Metrics	Time: 0	Time: 1	Time: 2	Time: 3	Time: 4	Time: 5
C1	N risk	37	36	35	30	14	1
	Events	0	0	1	1	1	1
	Survival	1	1	0.9722	0.9722	0.9722	0.9722
C2	N risk	40	38	37	26	10	0
	Events	0	1	3	7	9	9
	Survival	1	0.9744	0.9231	0.8205	0.7308	0.7308

PMID: 21989116 − Head and neck cancer (Oral SCC)
CINSARC coverage: 67 genes (100%)

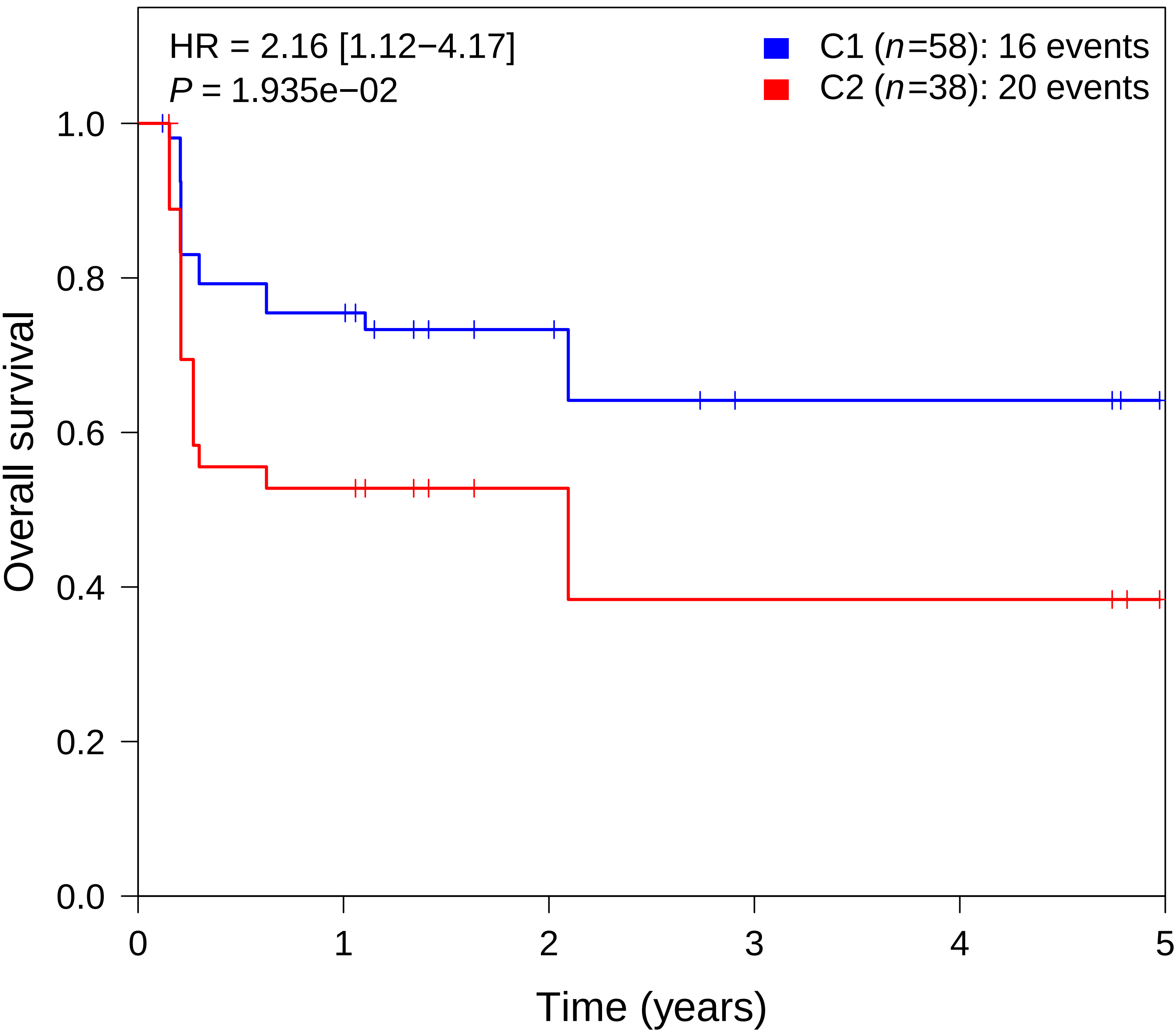

Group	Metrics	Time: 0	Time: 1	Time: 2	Time: 3	Time: 4	Time: 5
C1	N risk	58	40	20	8	8	0
	Events	0	13	14	16	16	16
	Survival	1	0.7547	0.7332	0.6415	0.6415	0.6415
C2	N risk	38	19	11	8	8	0
	Events	0	17	17	20	20	20
	Survival	1	0.5278	0.5278	0.3838	0.3838	0.3838

PMID: 18270328 − Hematopoietic cancer (AML)
CINSARC coverage: 67 genes (100%)

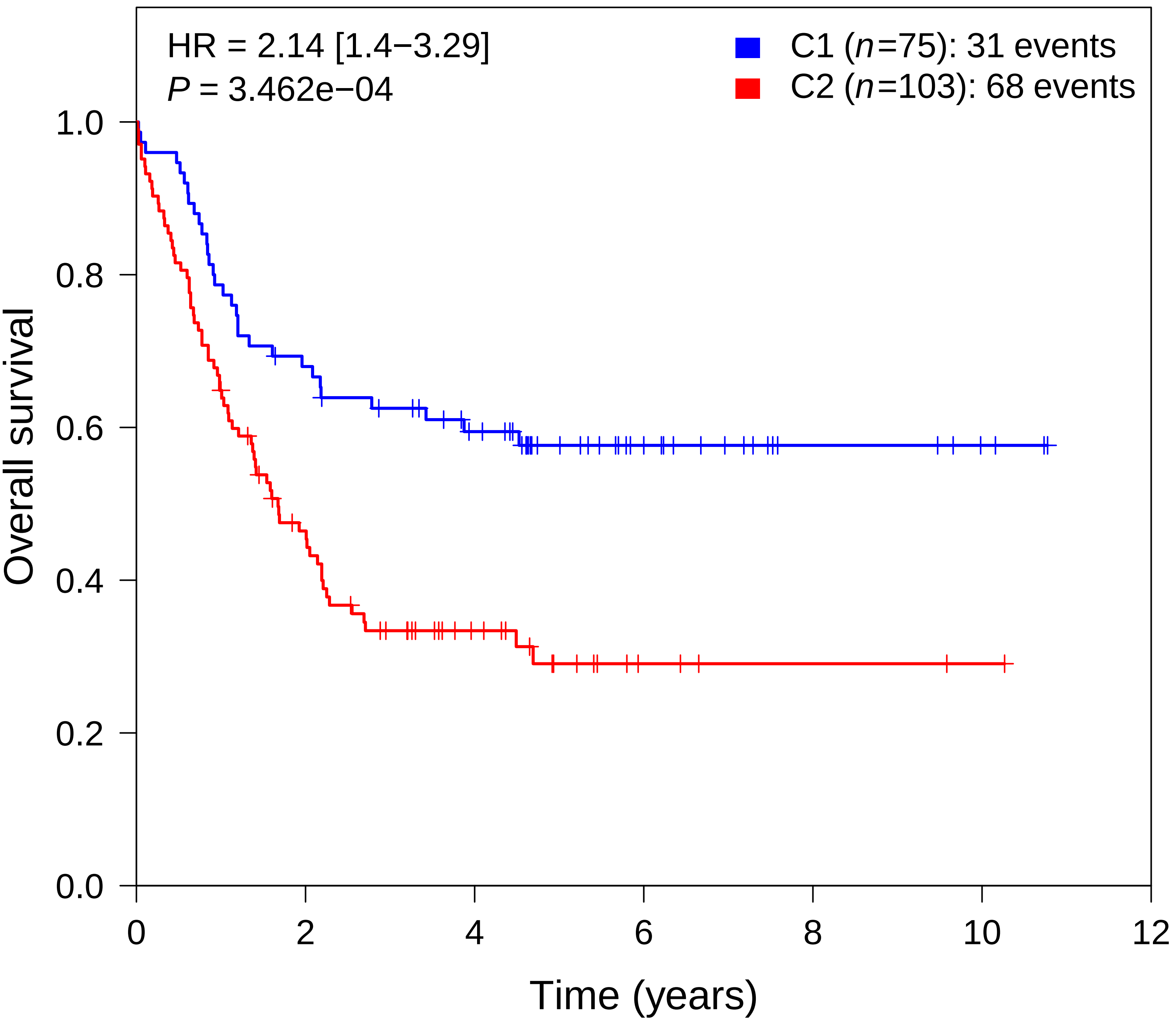

Group	Metrics	Time: 0	Time: 1	Time: 2	Time: 3	Time: 4	Time: 5
C1	N risk	75	59	50	44	37	25
	Events	0	16	24	28	30	31
	Survival	1	0.7867	0.6797	0.6251	0.5945	0.5765
C2	N risk	103	66	43	28	19	9
	Events	0	36	54	66	66	68
	Survival	1	0.6486	0.4644	0.3339	0.3339	0.2906

PMID: 17410195 − Hematopoietic cancer (B_ALL)
CINSARC coverage: 67 genes (100%)

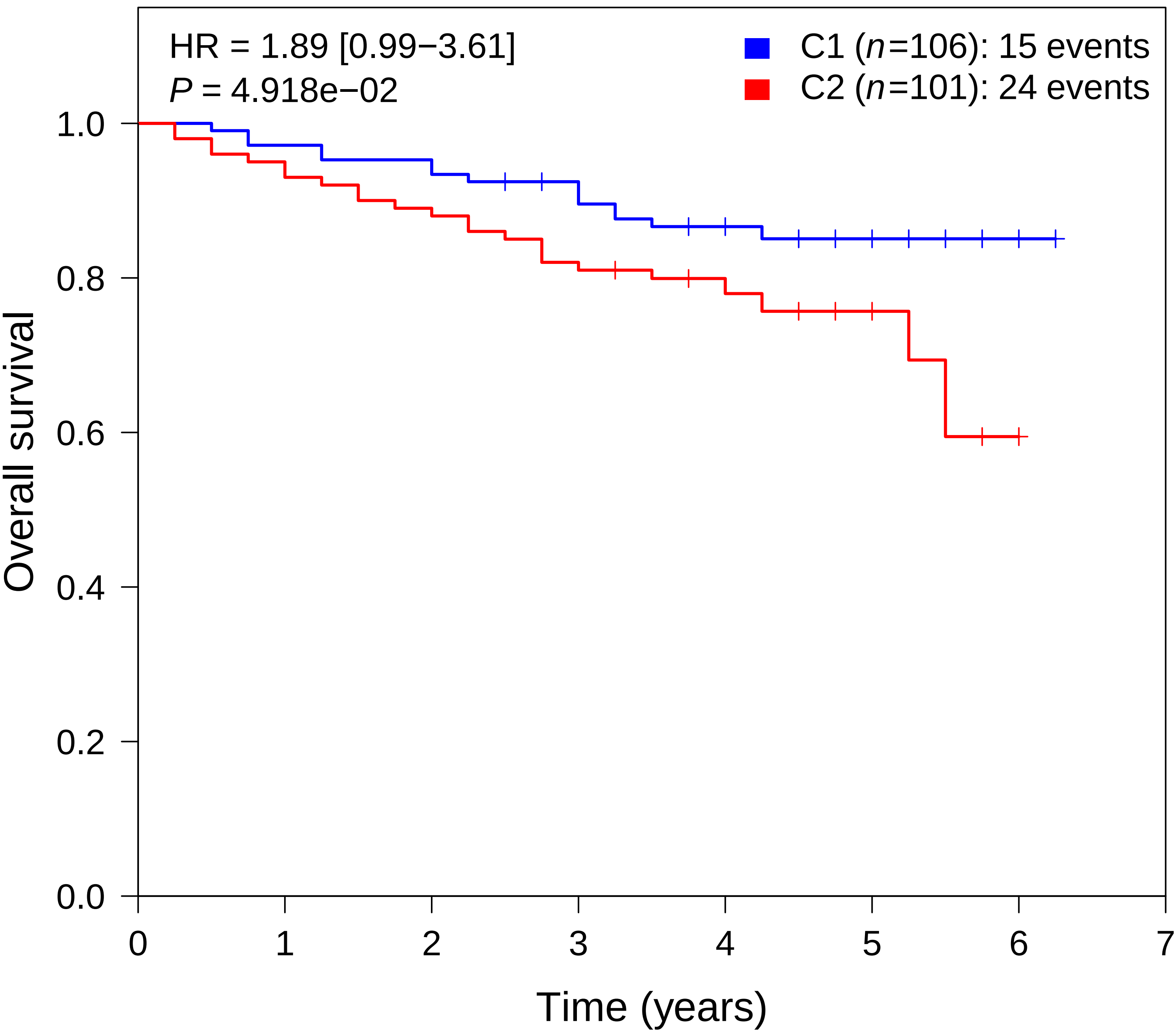

Group	Metrics	Time: 0	Time: 1	Time: 2	Time: 3	Time: 4	Time: 5
C1	N risk	106	103	101	96	64	25
	Events	0	3	7	11	14	15
	Survival	1	0.9717	0.934	0.8956	0.8664	0.8507
C2	N risk	101	95	89	81	41	16
	Events	0	7	12	19	21	22
	Survival	1	0.9302	0.8802	0.81	0.7797	0.7568

PMID: 21625232 − Hematopoietic cancer (CLL)
CINSARC coverage: 67 genes (100%)

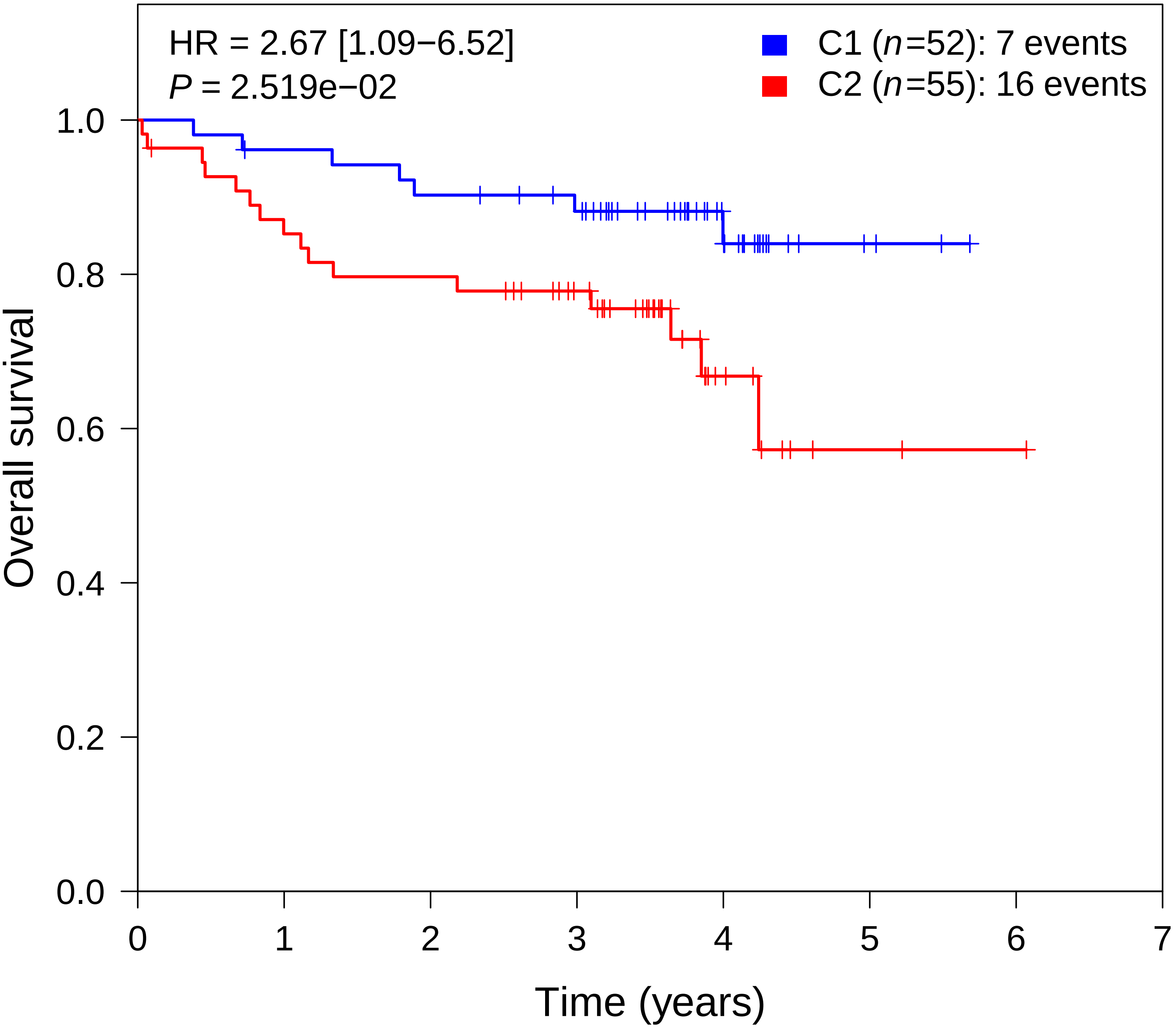

Group	Metrics	Time: 0	Time: 1	Time: 2	Time: 3	Time: 4	Time: 5
C1	N risk	52	49	46	42	20	3
	Events	0	2	5	6	7	7
	Survival	1	0.9615	0.9027	0.8817	0.8397	0.8397
C2	N risk	55	46	43	35	9	2
	Events	0	8	11	12	15	16
	Survival	1	0.8524	0.7969	0.7783	0.668	0.5725

PMID: 19038878 − Hematopoietic cancer (DLBCL)
CINSARC coverage: 67 genes (100%)

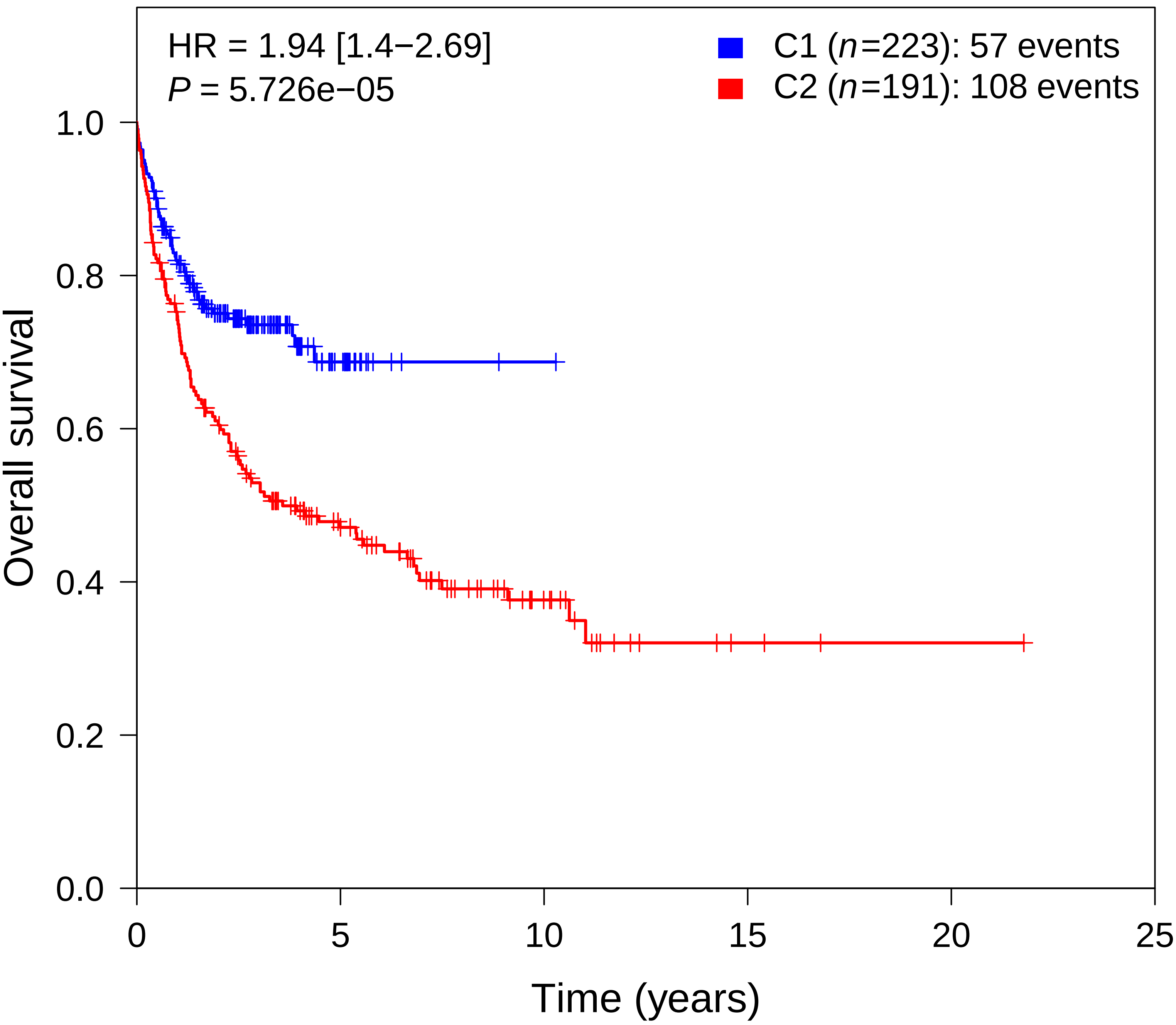

Group	Metrics	Time: 0	Time: 1	Time: 2	Time: 3	Time: 4	Time: 5
C1	N risk	223	165	118	75	43	24
	Events	1	39	52	54	56	57
	Survival	0.9955	0.8196	0.7505	0.7356	0.7073	0.6871
C2	N risk	191	137	108	89	75	63
	Events	1	49	74	87	93	96
	Survival	0.9948	0.7416	0.6045	0.5294	0.4927	0.4712

PMID: 16760442 − Hematopoietic cancer (DLBCL)
CINSARC coverage: 62 genes (92.54%)

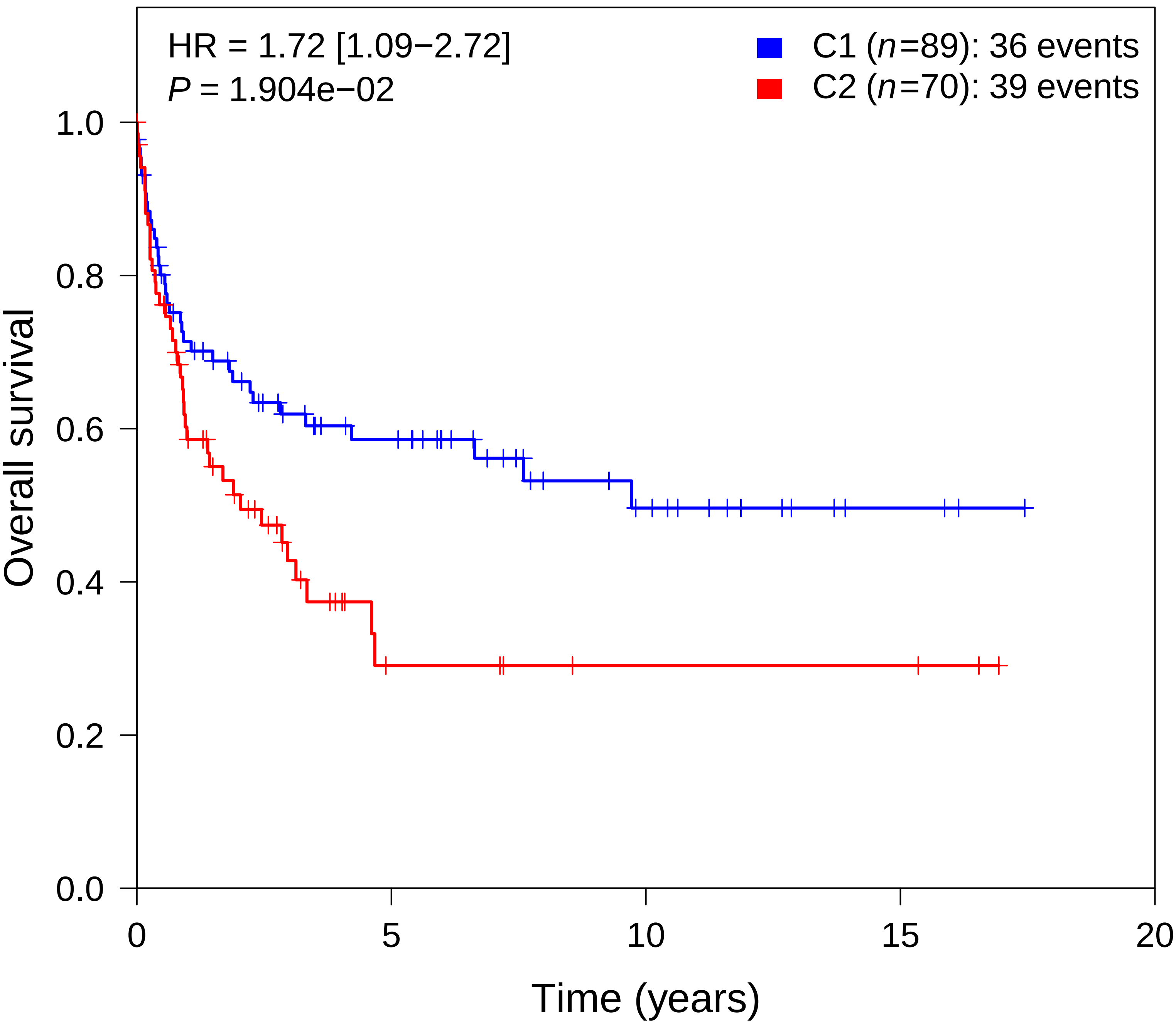

Group	Metrics	Time: 0	Time: 1	Time: 2	Time: 3	Time: 4	Time: 5
C1	N risk	89	57	49	41	35	33
	Events	2	24	28	31	32	33
	Survival	0.9775	0.7139	0.6614	0.6191	0.6036	0.5859
C2	N risk	70	36	27	17	11	6
	Events	0	27	31	35	37	39
	Survival	1	0.586	0.5138	0.4278	0.3739	0.2908

PMID: 15345589 − Hematopoietic cancer (FL)
CINSARC coverage: 38 genes (56.72%)

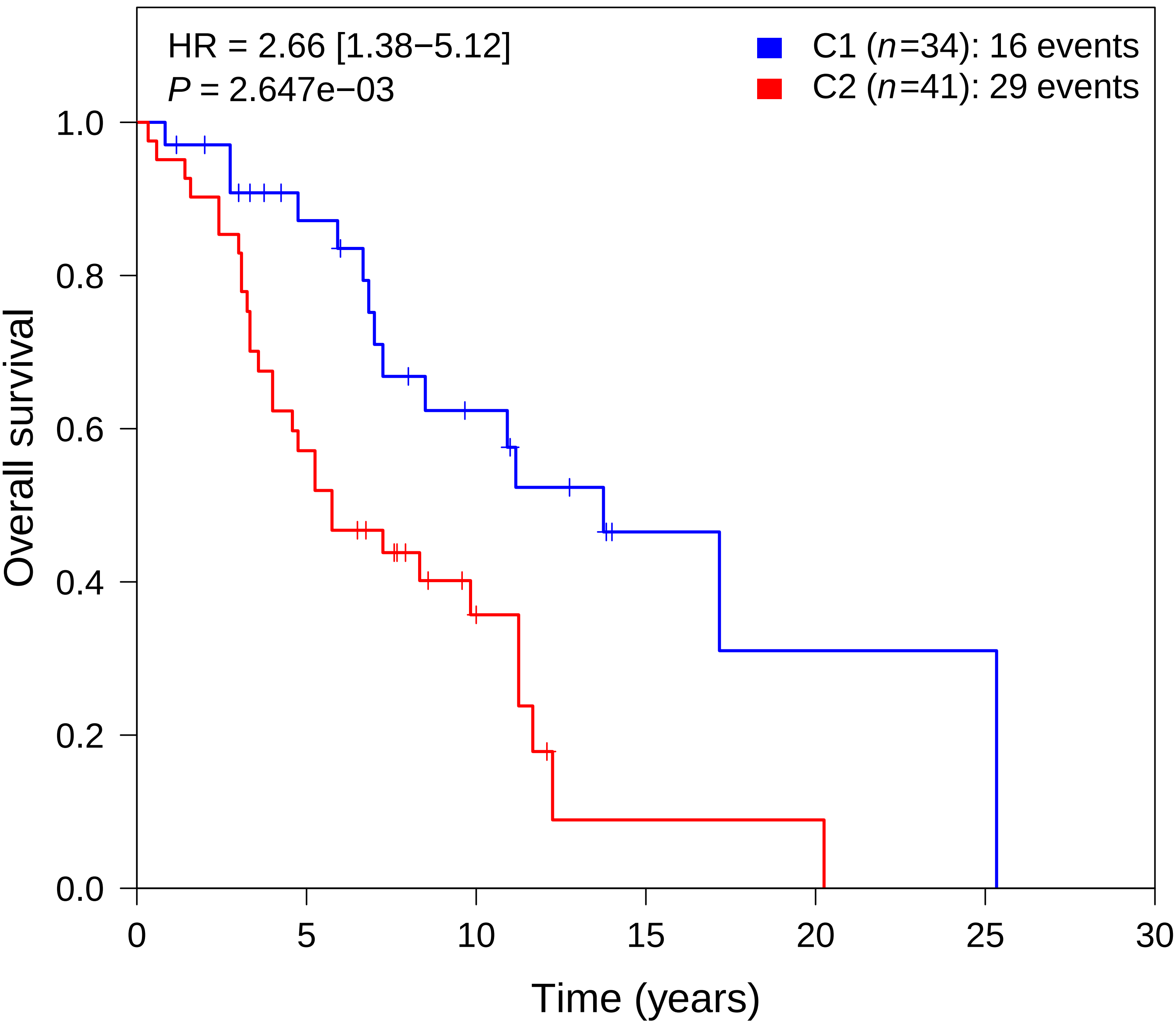

Group	Metrics	Time: 0	Time: 1	Time: 2	Time: 3	Time: 4	Time: 5
C1	N risk	34	33	32	29	26	24
	Events	0	1	1	3	3	4
	Survival	1	0.9706	0.9706	0.908	0.908	0.8717
C2	N risk	41	39	37	35	26	22
	Events	0	2	4	7	15	17
	Survival	1	0.9512	0.9024	0.8293	0.6232	0.5713

PMID: 18416826 − Hematopoietic cancer (Mantle cell lymphoma)
CINSARC coverage: 25 genes (37.31%)

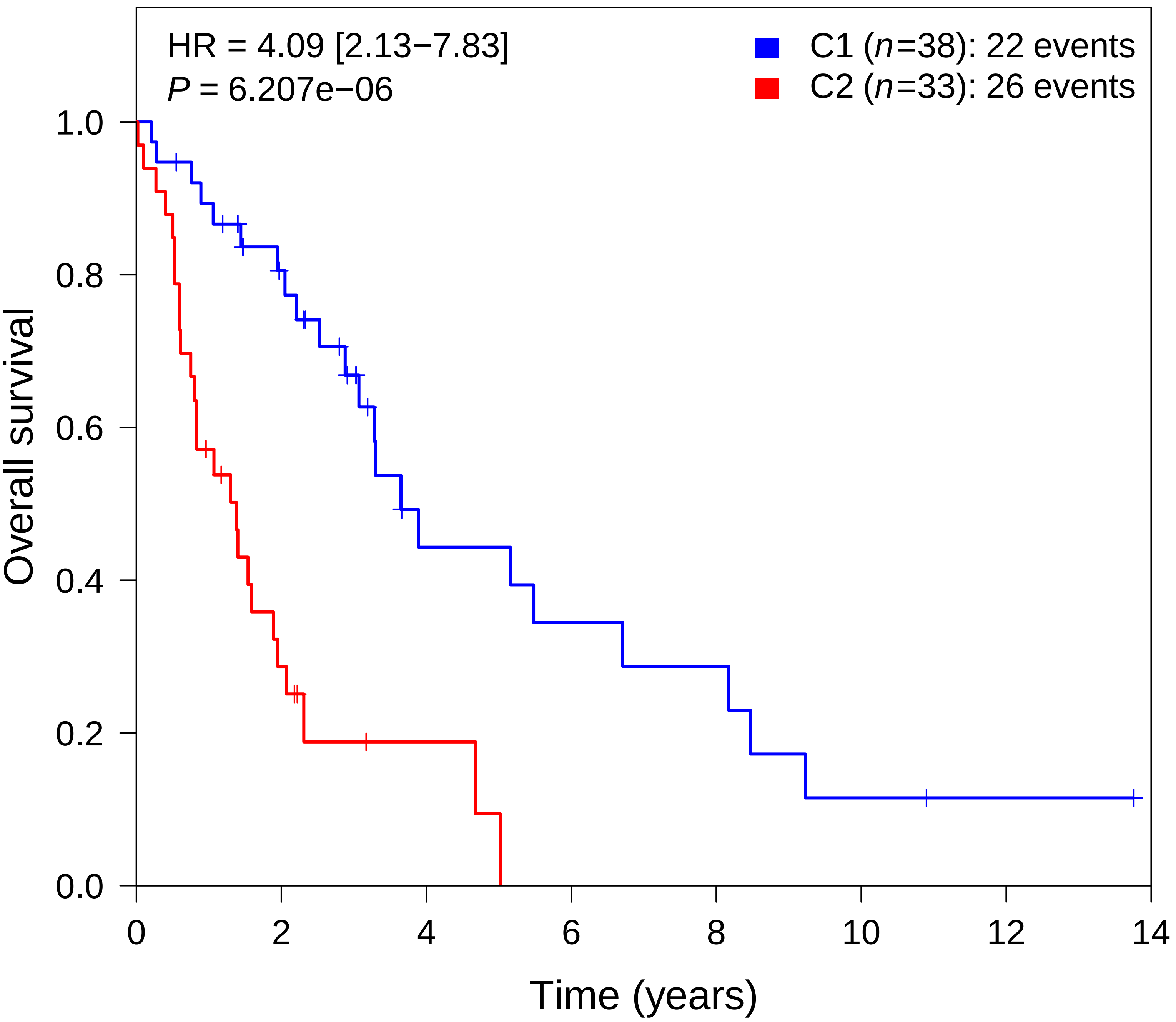

Group	Metrics	Time: 0	Time: 1	Time: 2	Time: 3	Time: 4	Time: 5
C1	N risk	38	33	25	17	9	9
	Events	0	4	7	11	16	16
	Survival	1	0.8932	0.8053	0.6685	0.4432	0.4432
C2	N risk	33	17	8	3	2	1
	Events	0	14	22	24	24	25
	Survival	1	0.5714	0.2868	0.1882	0.1882	0.0941

PMID: 17023574 − Hematopoietic cancer (Multiple myeloma)
CINSARC coverage: 67 genes (100%)

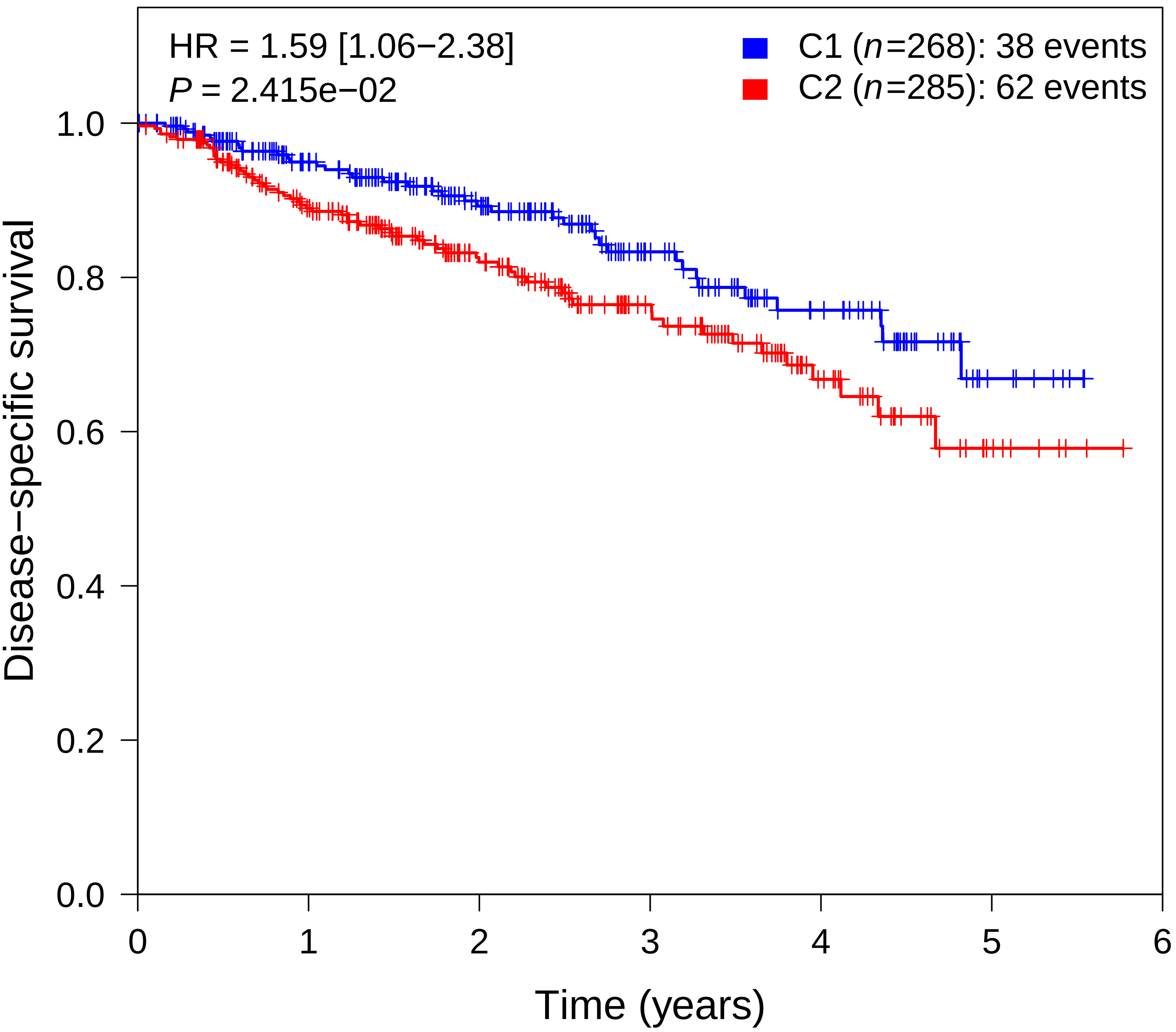

Group	Metrics	Time: 0	Time: 1	Time: 2	Time: 3	Time: 4	Time: 5
C1	N risk	268	196	132	77	45	9
	Events	0	12	22	29	35	38
	Survival	1	0.9496	0.8924	0.8332	0.7574	0.6687
C2	N risk	285	214	135	82	35	8
	Events	1	29	43	51	59	62
	Survival	0.9965	0.8897	0.8198	0.7646	0.6678	0.5784

PMID: 17409404 − Hematopoietic cancer (Multiple myeloma)
CINSARC coverage: 62 genes (92.54%)

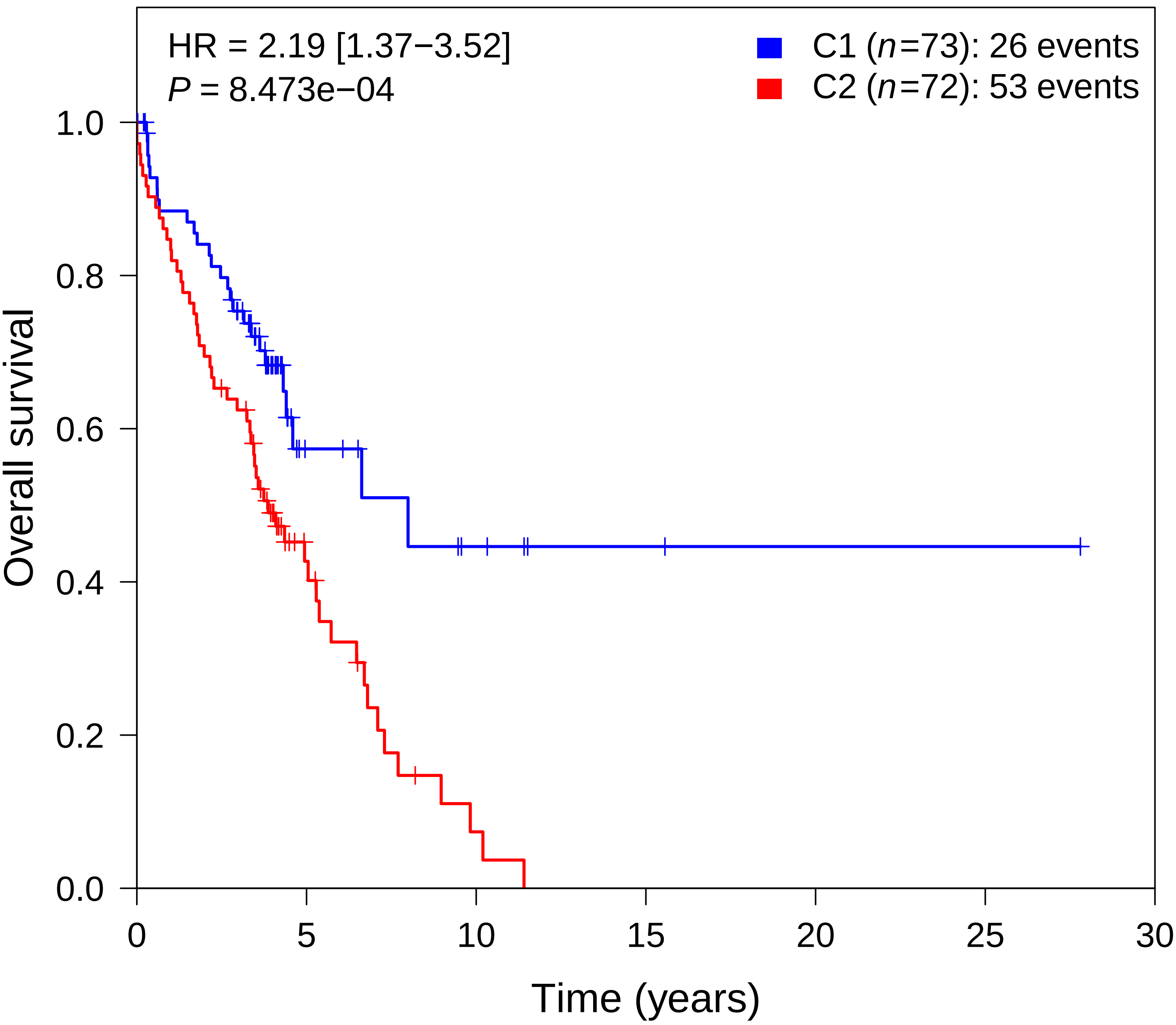

Group	Metrics	Time: 0	Time: 1	Time: 2	Time: 3	Time: 4	Time: 5
C1	N risk	73	61	58	48	27	11
	Events	0	8	11	17	21	24
	Survival	1	0.8842	0.8408	0.7535	0.6829	0.5736
C2	N risk	72	60	50	44	30	17
	Events	2	12	22	27	36	39
	Survival	0.9722	0.8333	0.6944	0.6244	0.4901	0.427

PMID: 18923165 − Liver cancer
CINSARC coverage: 41 genes (61.19%)

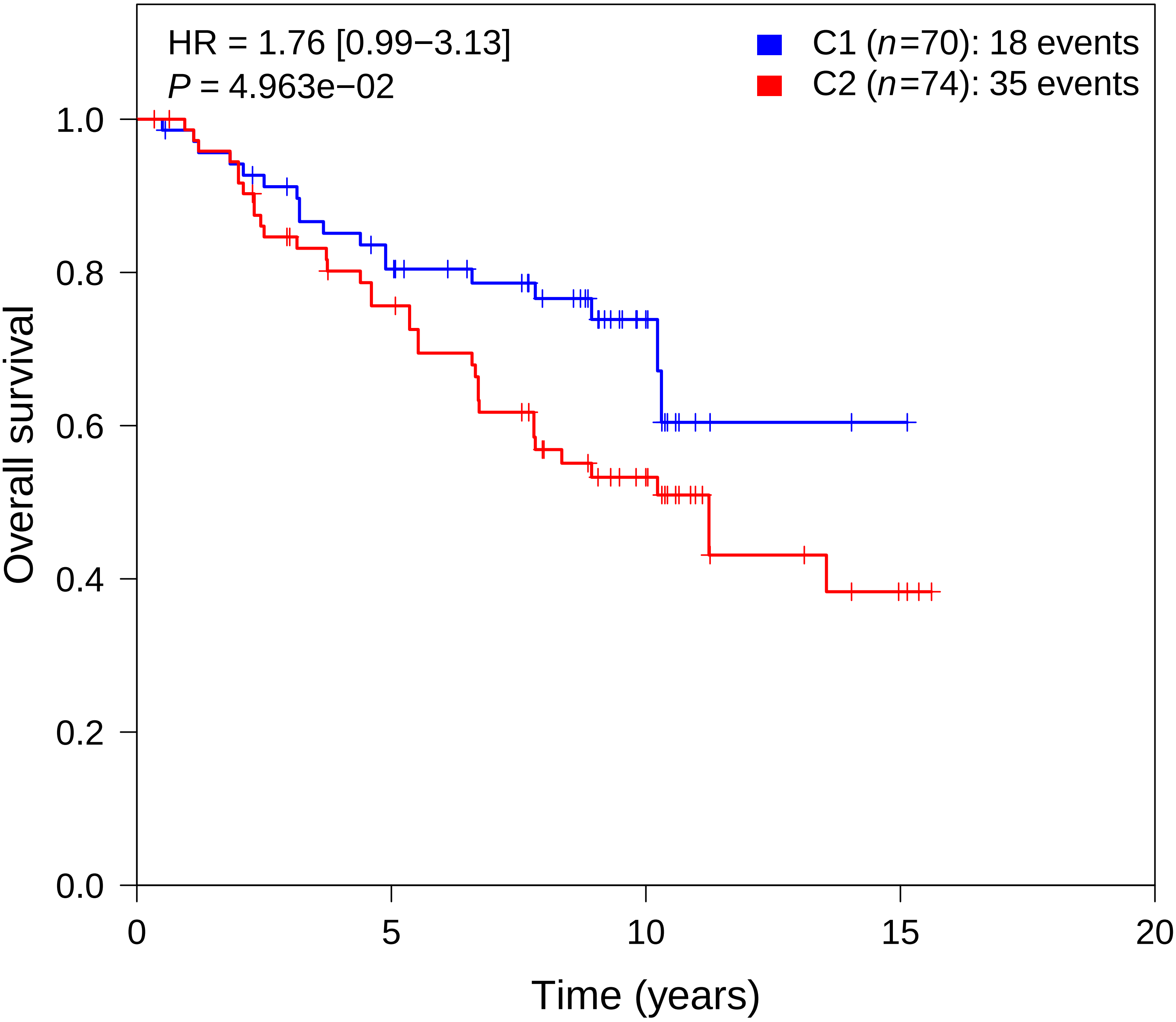

Group	Metrics	Time: 0	Time: 1	Time: 2	Time: 3	Time: 4	Time: 5
C1	N risk	70	67	64	60	56	51
	Events	0	1	4	6	10	13
	Survival	1	0.9857	0.9416	0.9119	0.8511	0.8044
C2	N risk	74	71	66	59	53	50
	Events	0	1	6	11	14	17
	Survival	1	0.9861	0.9167	0.8464	0.8018	0.7564

PMID: 15565109 − Liver cancer
CINSARC coverage: 66 genes (98.51%)

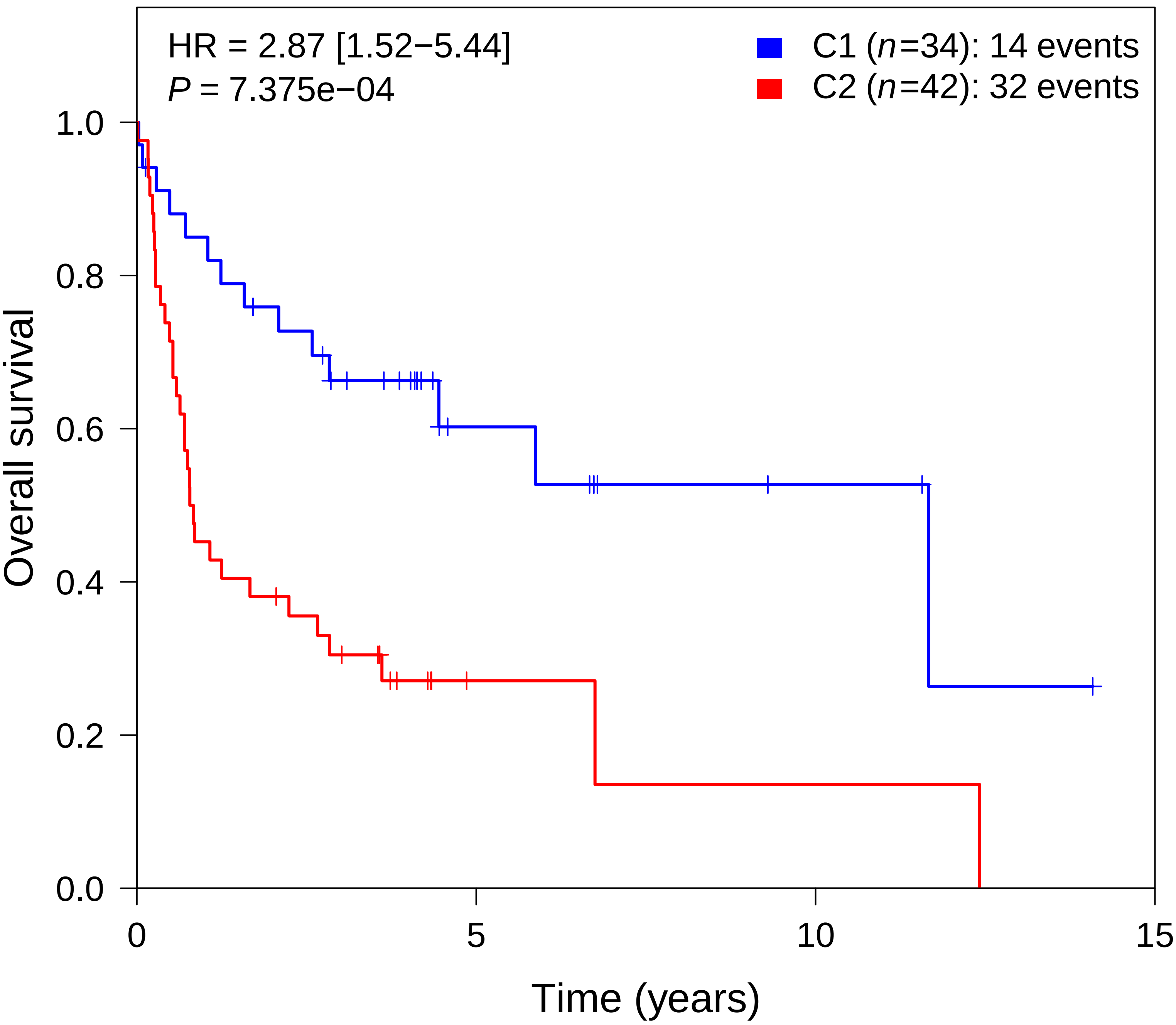

Group	Metrics	Time: 0	Time: 1	Time: 2	Time: 3	Time: 4	Time: 5
C1	N risk	34	28	24	19	16	8
	Events	0	5	8	11	11	12
	Survival	1	0.8501	0.759	0.6626	0.6626	0.6024
C2	N risk	42	19	16	12	6	2
	Events	0	23	26	29	30	30
	Survival	1	0.4524	0.381	0.3048	0.2709	0.2709

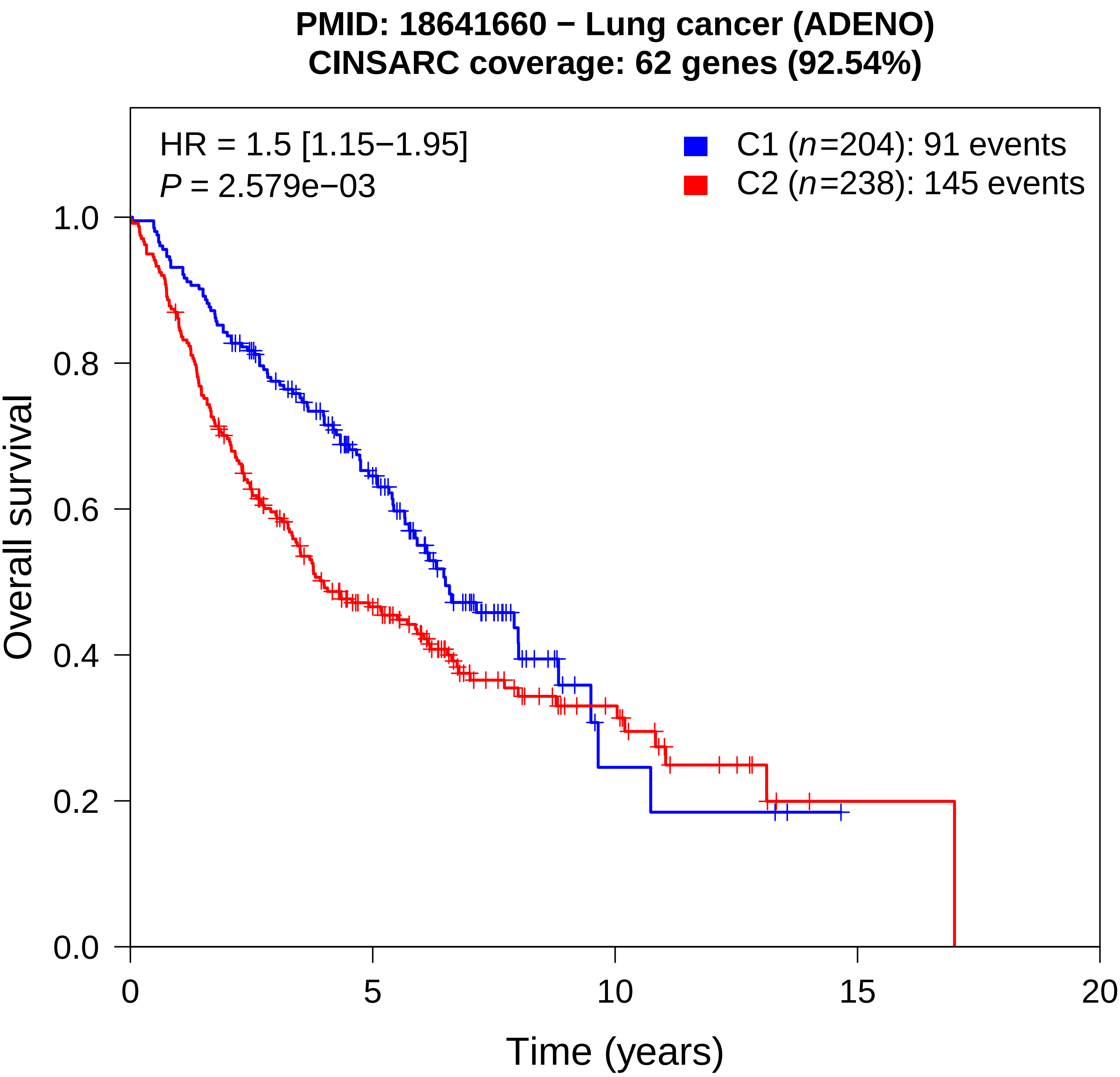

Group	Metrics	Time: 0	Time: 1	Time: 2	Time: 3	Time: 4	Time: 5
C1	N risk	204	189	169	146	115	87
	Events	0	14	33	45	55	65
	Survival	1	0.9312	0.8372	0.7751	0.715	0.6454
C2	N risk	238	203	163	132	102	84
	Events	0	35	72	96	117	122
	Survival	1	0.8529	0.6964	0.5916	0.4919	0.466

PMID: 19414676 − Lung cancer (ADENO)
CINSARC coverage: 67 genes (100%)

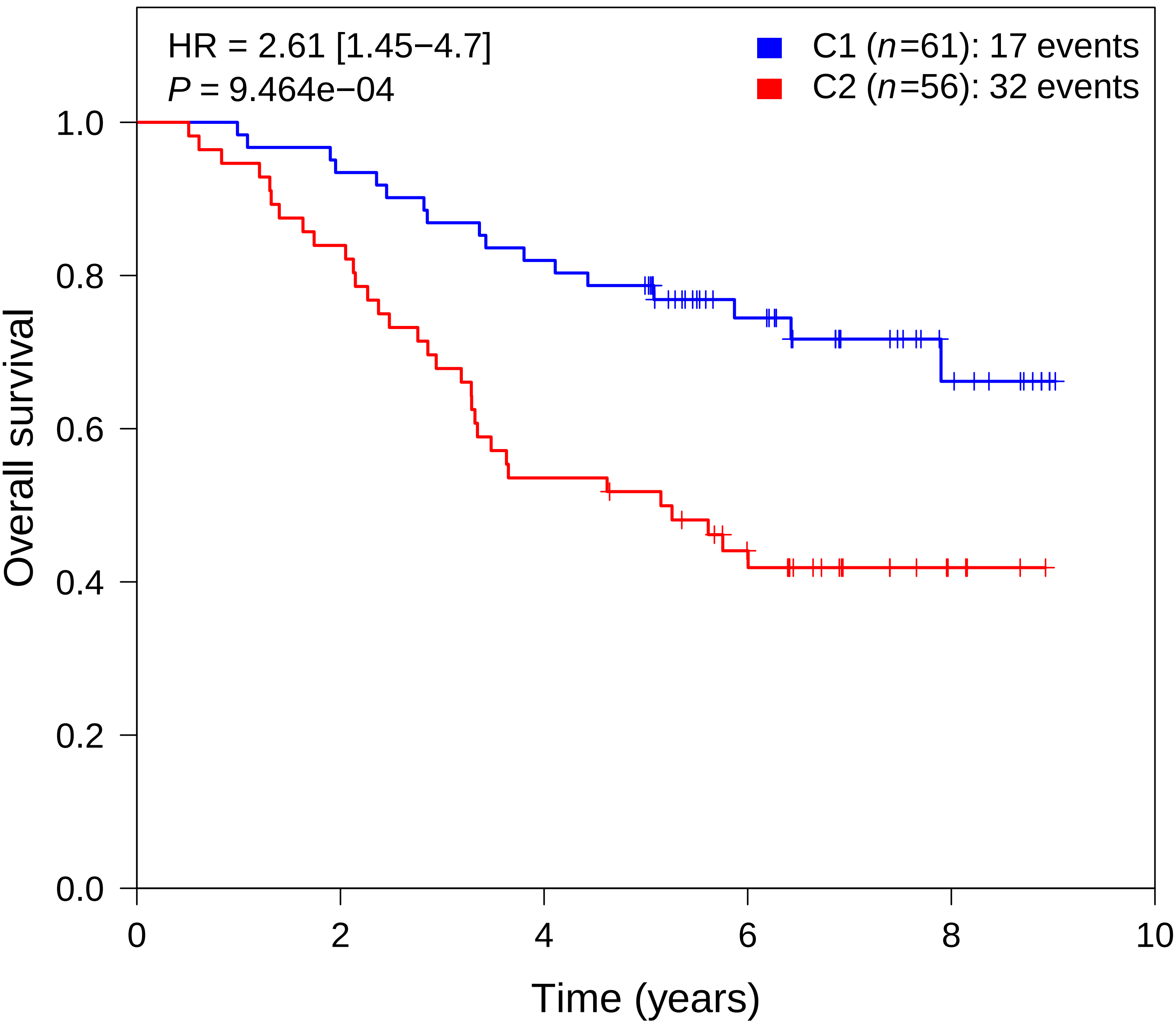

Group	Metrics	Time: 0	Time: 1	Time: 2	Time: 3	Time: 4	Time: 5
C1	N risk	61	60	57	53	50	47
	Events	0	1	4	8	11	13
	Survival	1	0.9836	0.9344	0.8689	0.8197	0.7869
C2	N risk	56	53	47	38	30	28
	Events	0	3	9	18	26	27
	Survival	1	0.9464	0.8393	0.6786	0.5357	0.5179

PMID: 22080568 − Lung cancer (ADENO)
CINSARC coverage: 67 genes (100%)

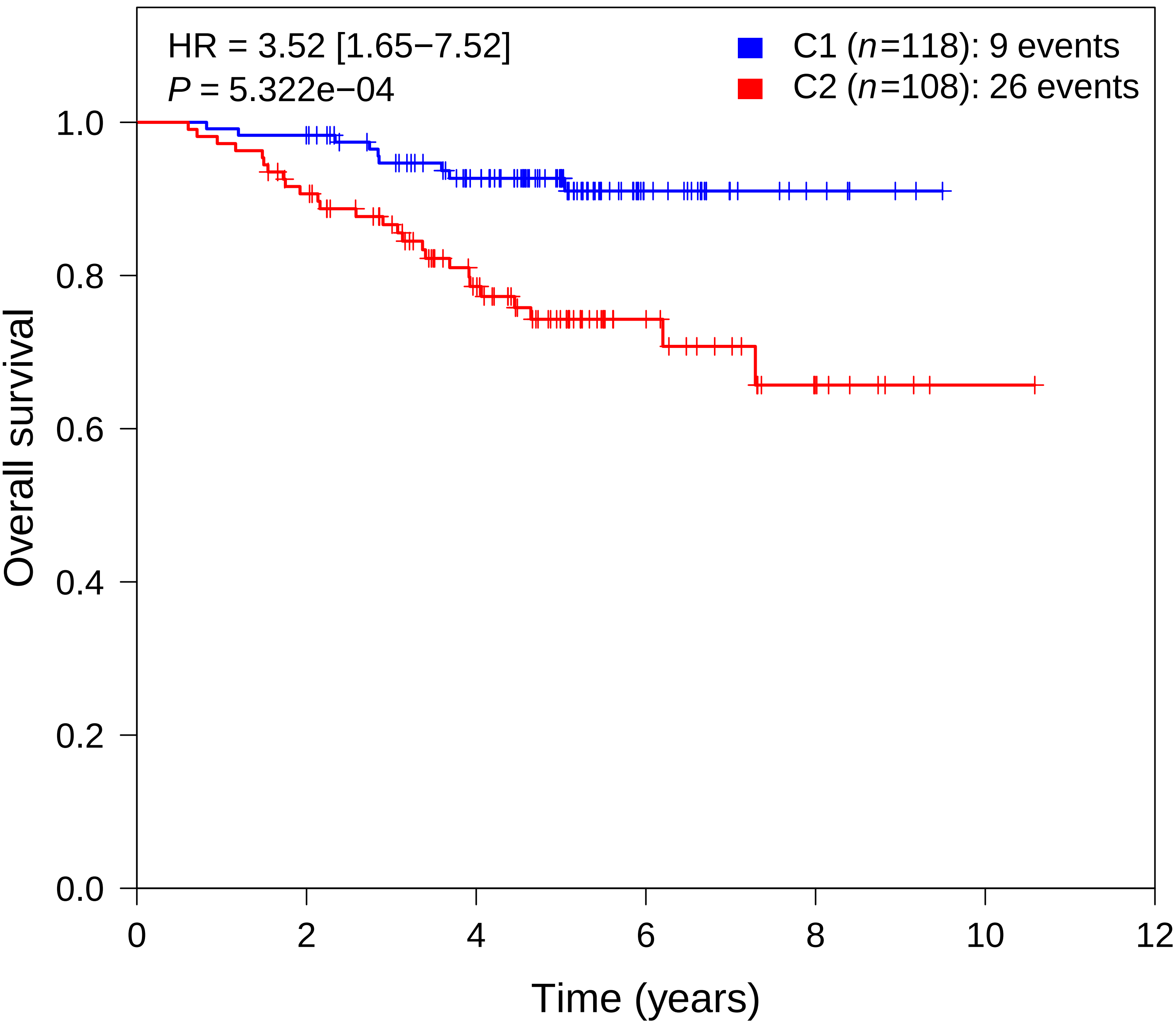

Group	Metrics	Time: 0	Time: 1	Time: 2	Time: 3	Time: 4	Time: 5
C1	N risk	118	117	115	103	87	61
	Events	0	1	2	6	8	8
	Survival	1	0.9915	0.9831	0.9468	0.9269	0.9269
C2	N risk	108	105	95	82	63	42
	Events	0	3	10	14	21	24
	Survival	1	0.9722	0.9067	0.8664	0.7857	0.7429

PMID: 16549822 − Lung cancer (SCC)
CINSARC coverage: 54 genes (80.6%)

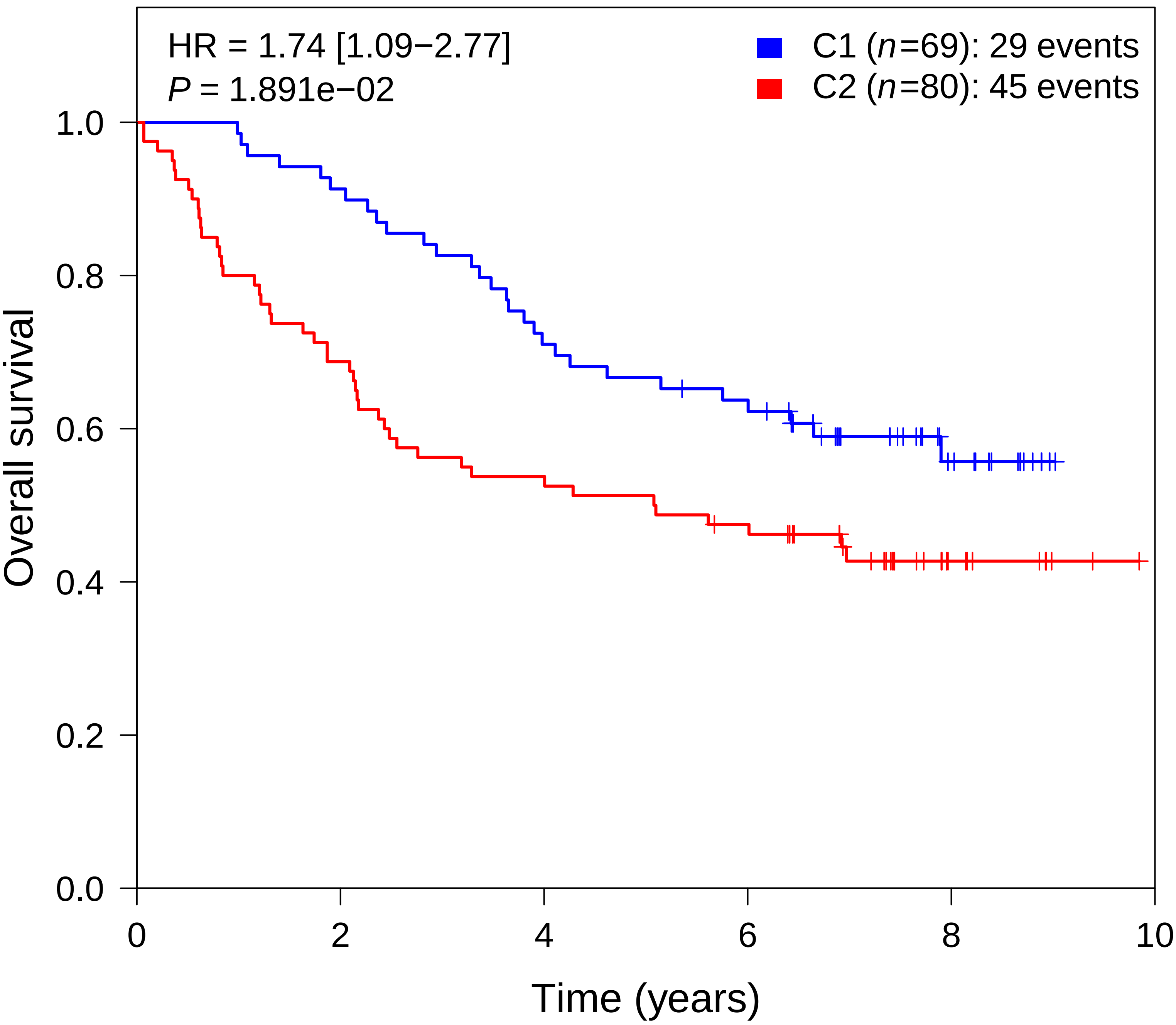

Group	Metrics	Time: 0	Time: 1	Time: 2	Time: 3	Time: 4	Time: 5
C1	N risk	69	68	63	57	49	46
	Events	0	1	6	12	20	23
	Survival	1	0.9855	0.913	0.8261	0.7101	0.6667
C2	N risk	80	64	55	45	43	41
	Events	0	16	25	35	37	39
	Survival	1	0.8	0.6875	0.5625	0.5375	0.5125

PMID: 19010856 − Lung cancer (SCC)
CINSARC coverage: 66 genes (98.51%)

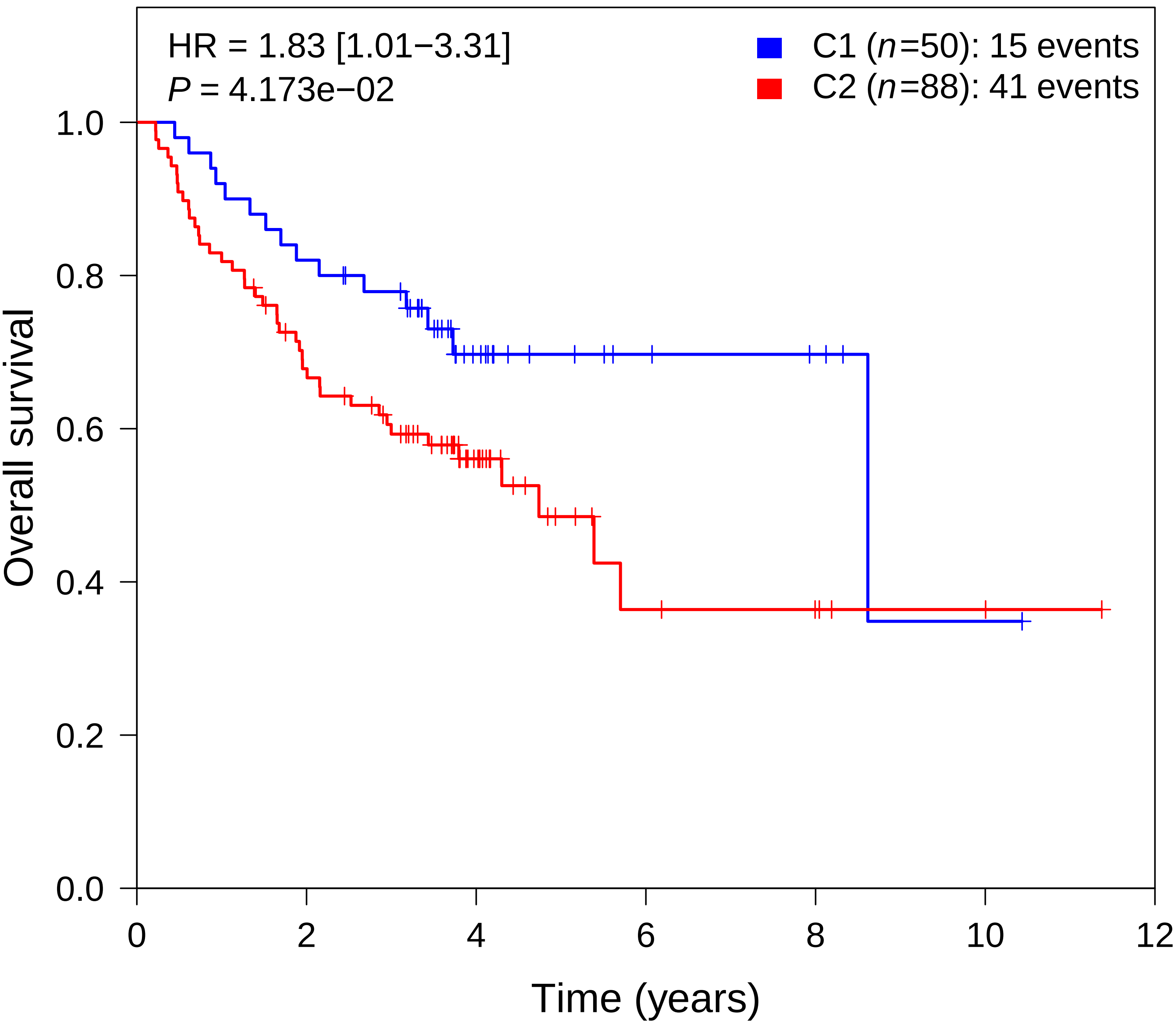

Group	Metrics	Time: 0	Time: 1	Time: 2	Time: 3	Time: 4	Time: 5
C1	N risk	50	46	41	37	16	9
	Events	0	4	9	11	14	14
	Survival	1	0.92	0.82	0.7789	0.6971	0.6971
C2	N risk	88	72	57	47	24	10
	Events	0	16	28	35	37	39
	Survival	1	0.8182	0.6783	0.5929	0.5607	0.4852

PMID: 19118056 − Lung cancer (SCC)
CINSARC coverage: 55 genes (82.09%)

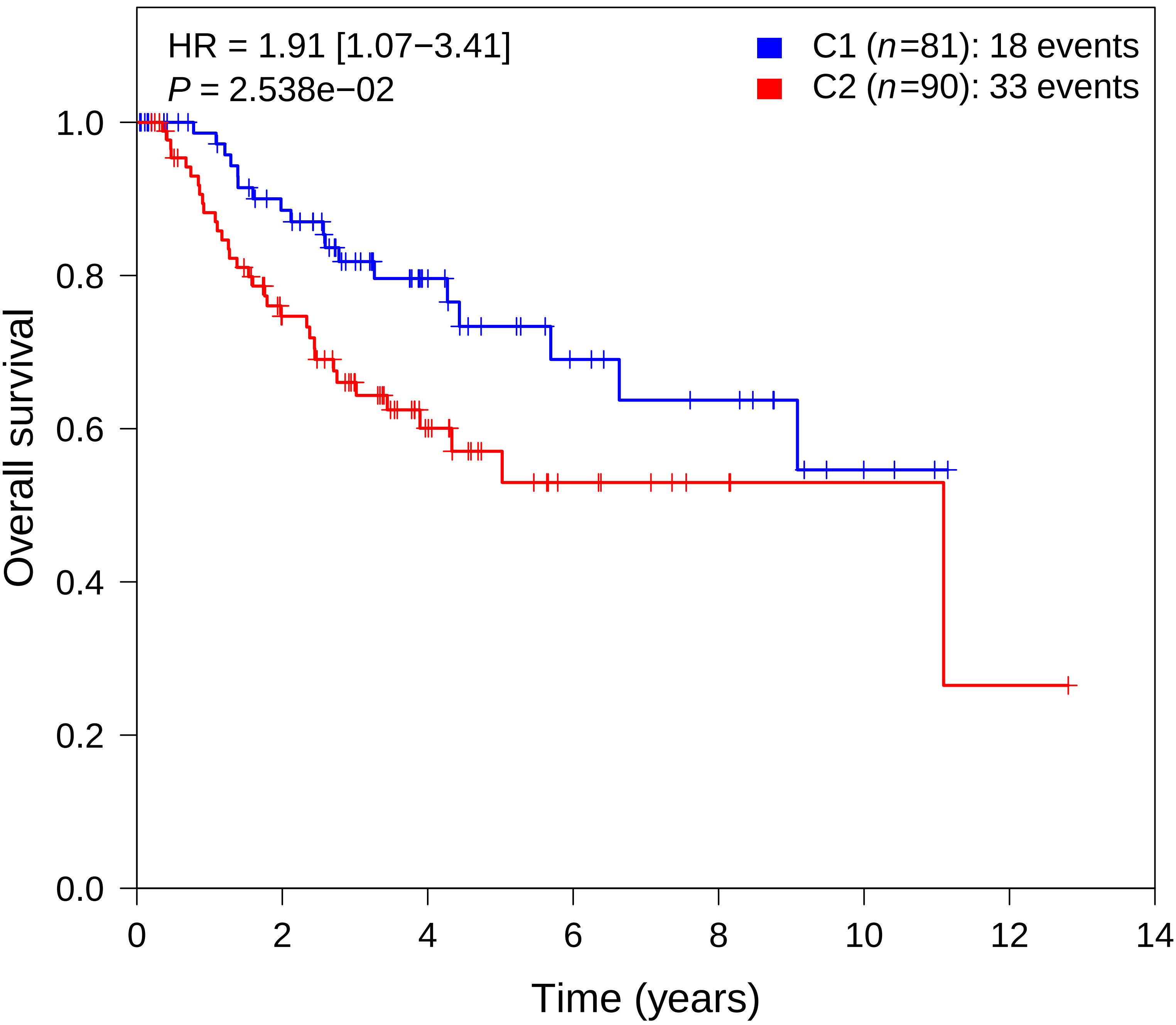

Group	Metrics	Time: 0	Time: 1	Time: 2	Time: 3	Time: 4	Time: 5
C1	N risk	81	70	59	43	28	20
	Events	0	1	8	12	13	15
	Survival	1	0.9859	0.8851	0.8182	0.796	0.7335
C2	N risk	90	74	53	40	24	14
	Events	0	10	21	27	30	31
	Survival	1	0.8821	0.7468	0.6605	0.6006	0.5705

PMID: 19192944 − Ovarian cancer
CINSARC coverage: 66 genes (98.51%)

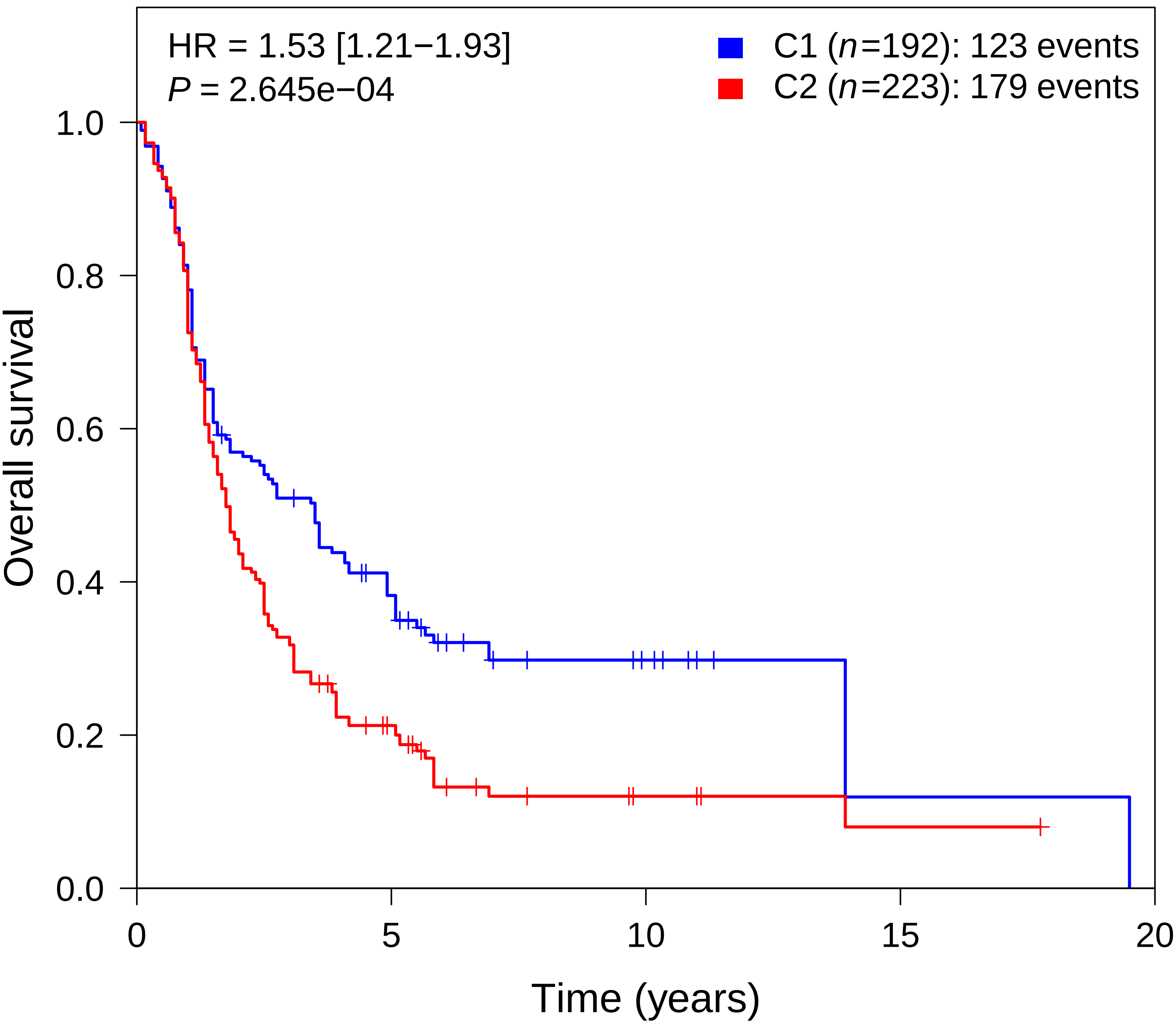

Group	Metrics	Time: 0	Time: 1	Time: 2	Time: 3	Time: 4	Time: 5
C1	N risk	192	151	101	82	66	47
	Events	0	41	80	90	101	109
	Survival	1	0.7811	0.5693	0.5093	0.4382	0.3823
C2	N risk	223	179	96	65	41	34
	Events	0	61	123	147	165	167
	Survival	1	0.7253	0.4365	0.3177	0.2234	0.2125

PMID: 19294737 − Ovarian cancer
CINSARC coverage: 62 genes (92.54%)

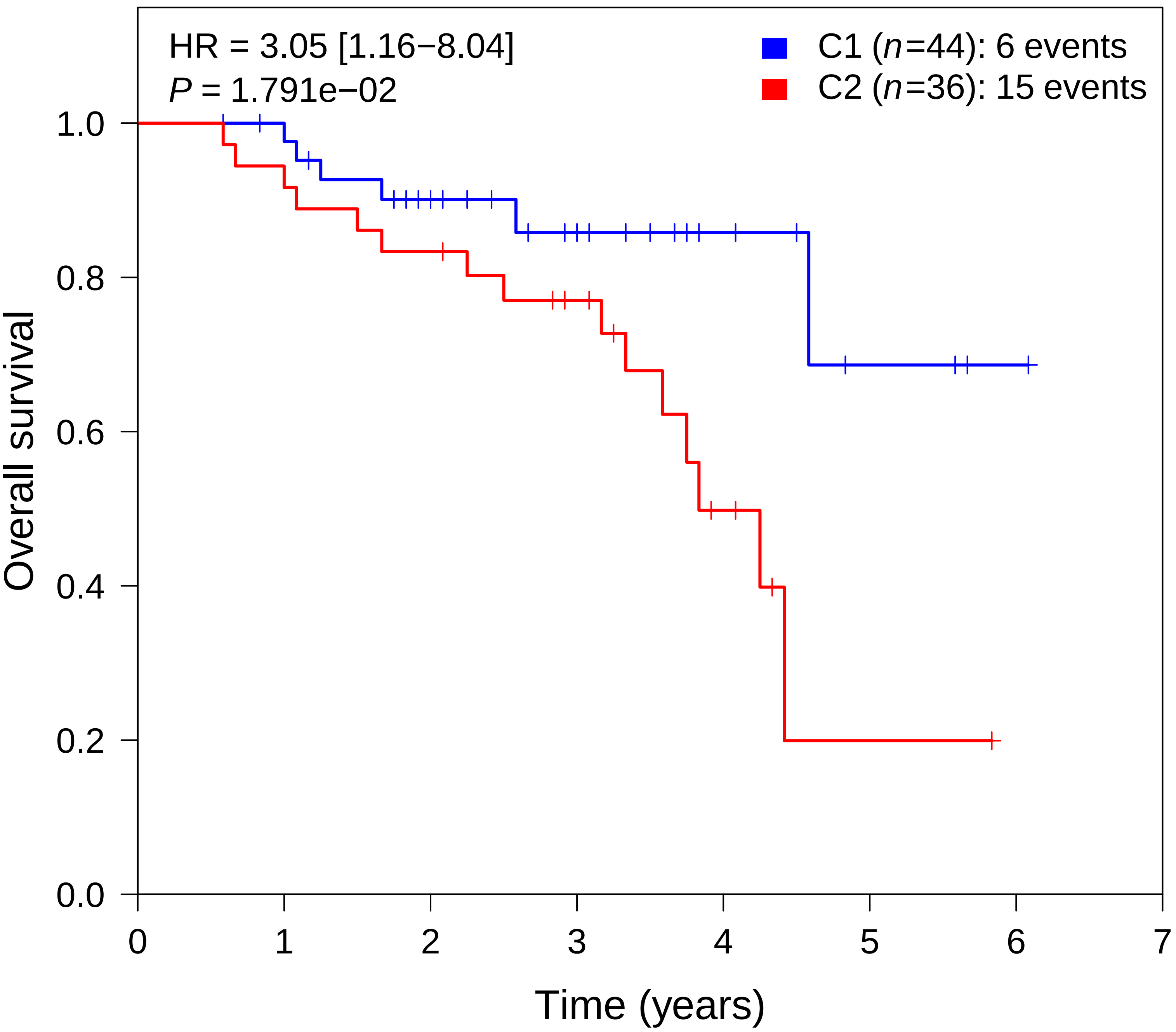

Group	Metrics	Time: 0	Time: 1	Time: 2	Time: 3	Time: 4	Time: 5
C1	N risk	44	42	27	18	7	3
	Events	0	1	4	5	5	6
	Survival	1	0.9762	0.901	0.8581	0.8581	0.6865
C2	N risk	36	34	29	20	7	1
	Events	0	3	6	8	13	15
	Survival	1	0.9167	0.8333	0.7704	0.498	0.1992

PMID: 16273092 − Ovarian cancer
CINSARC coverage: 62 genes (92.54%)

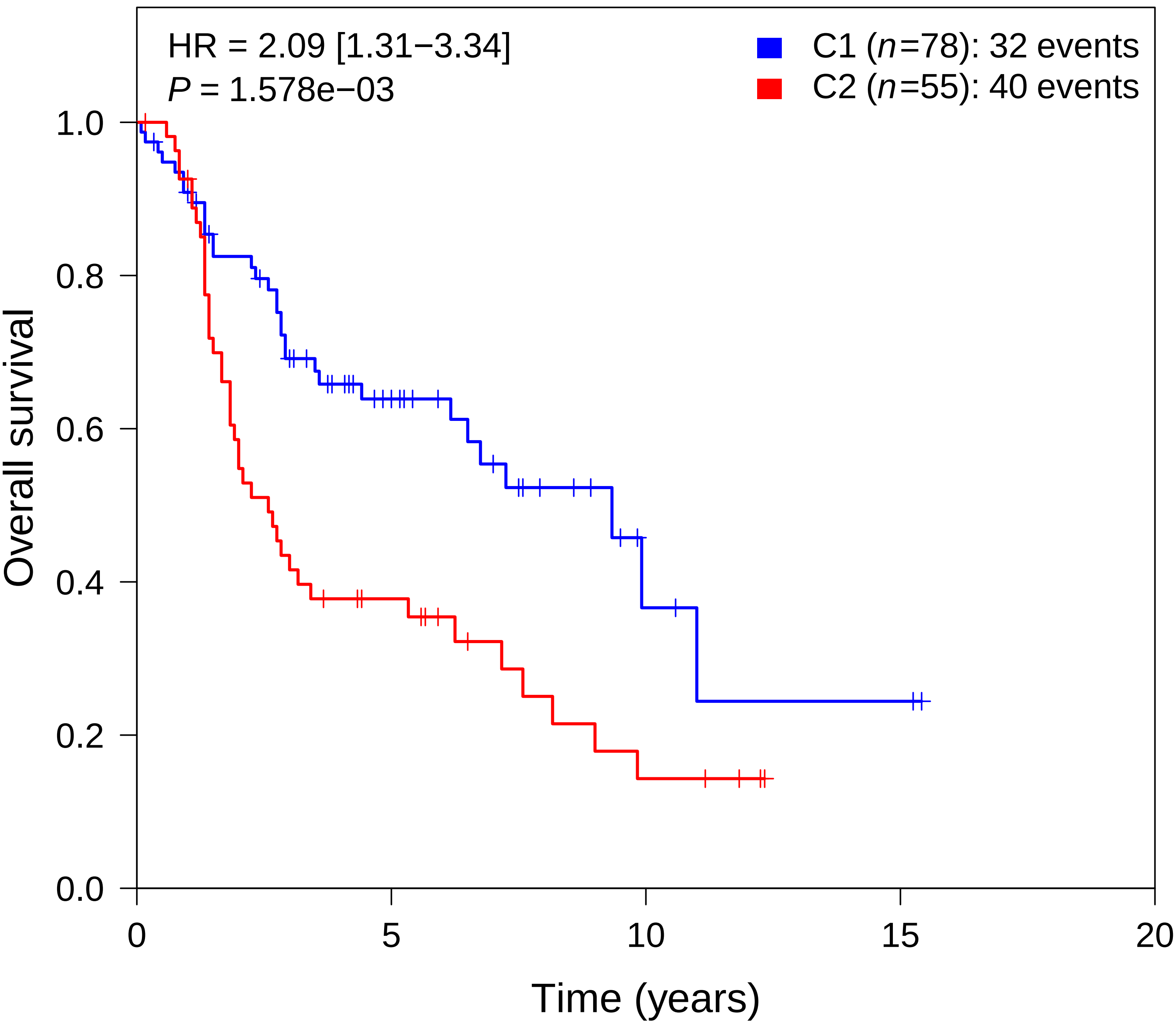

Group	Metrics	Time: 0	Time: 1	Time: 2	Time: 3	Time: 4	Time: 5
C1	N risk	78	69	57	45	37	31
	Events	0	7	13	22	24	25
	Survival	1	0.9085	0.8249	0.6915	0.6582	0.6388
C2	N risk	55	50	31	23	19	16
	Events	0	4	24	31	33	33
	Survival	1	0.9259	0.548	0.4157	0.3779	0.3779

PMID: 21720365 − Ovarian cancer
CINSARC coverage: 62 genes (92.54%)

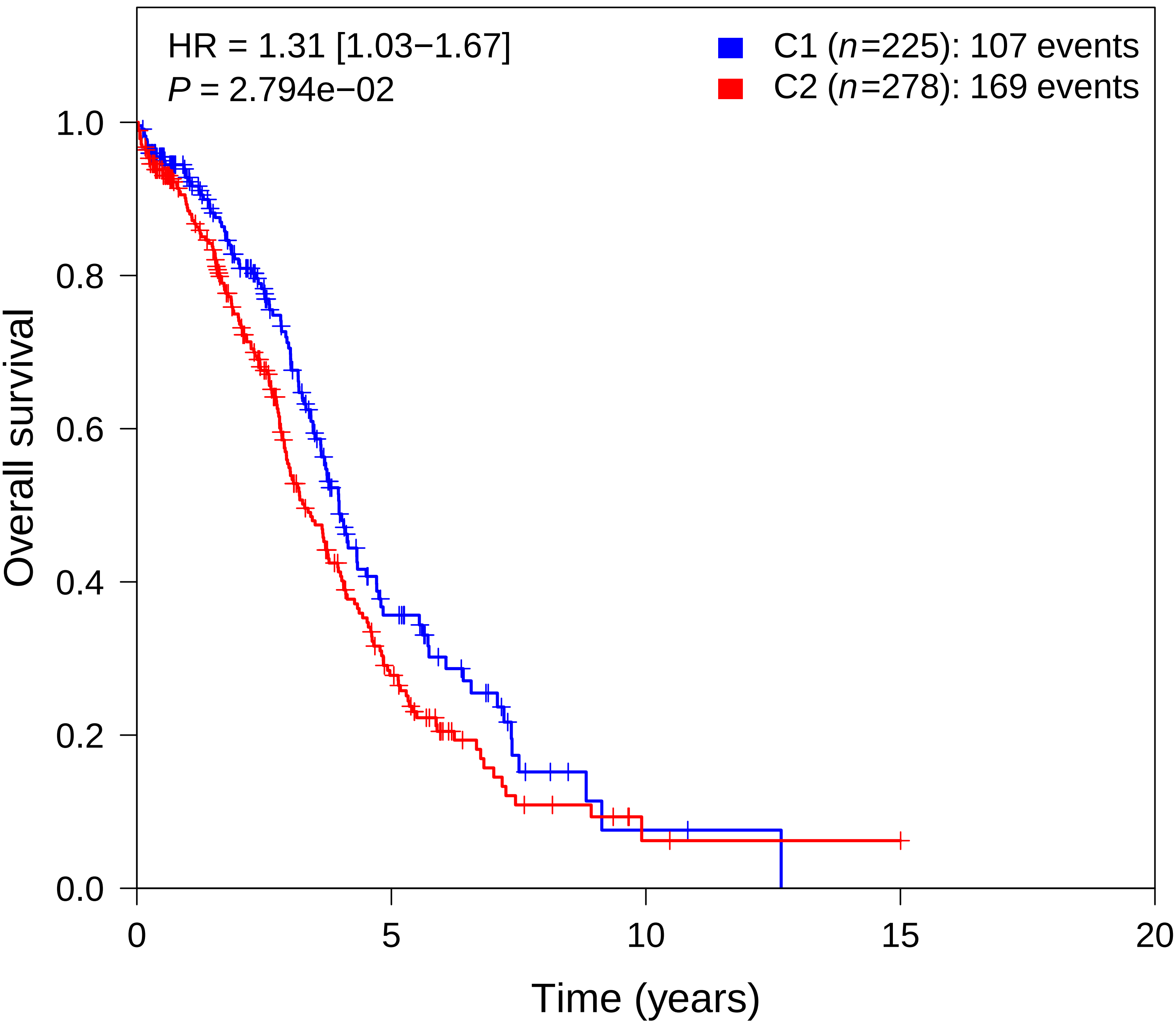

Group	Metrics	Time: 0	Time: 1	Time: 2	Time: 3	Time: 4	Time: 5
C1	N risk	225	166	132	98	56	33
	Events	0	15	34	50	78	92
	Survival	1	0.928	0.8155	0.7051	0.4887	0.3565
C2	N risk	278	210	165	106	71	43
	Events	0	30	62	102	127	149
	Survival	1	0.8843	0.7452	0.5491	0.413	0.278

PMID: 20233430 − Prostate cancer
CINSARC coverage: 41 genes (61.19%)

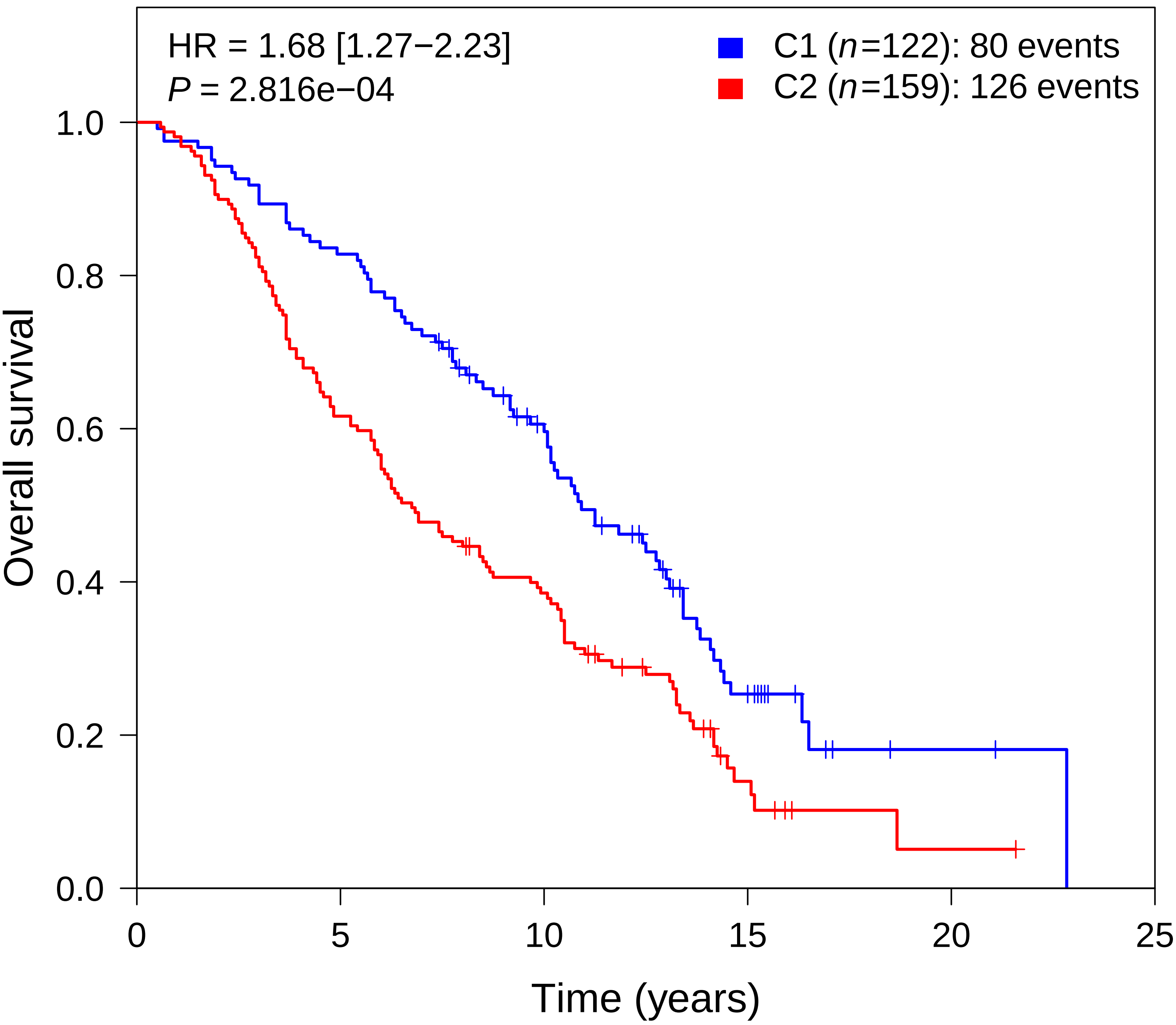

Group	Metrics	Time: 0	Time: 1	Time: 2	Time: 3	Time: 4	Time: 5
C1	N risk	122	119	115	112	105	101
	Events	0	3	7	13	17	21
	Survival	1	0.9754	0.9426	0.8934	0.8607	0.8279
C2	N risk	159	156	144	131	110	98
	Events	0	3	16	30	49	61
	Survival	1	0.9811	0.8994	0.8113	0.6918	0.6164

Supplementary figure 4

PMID: 18641660
Lung cancer (ADENO)

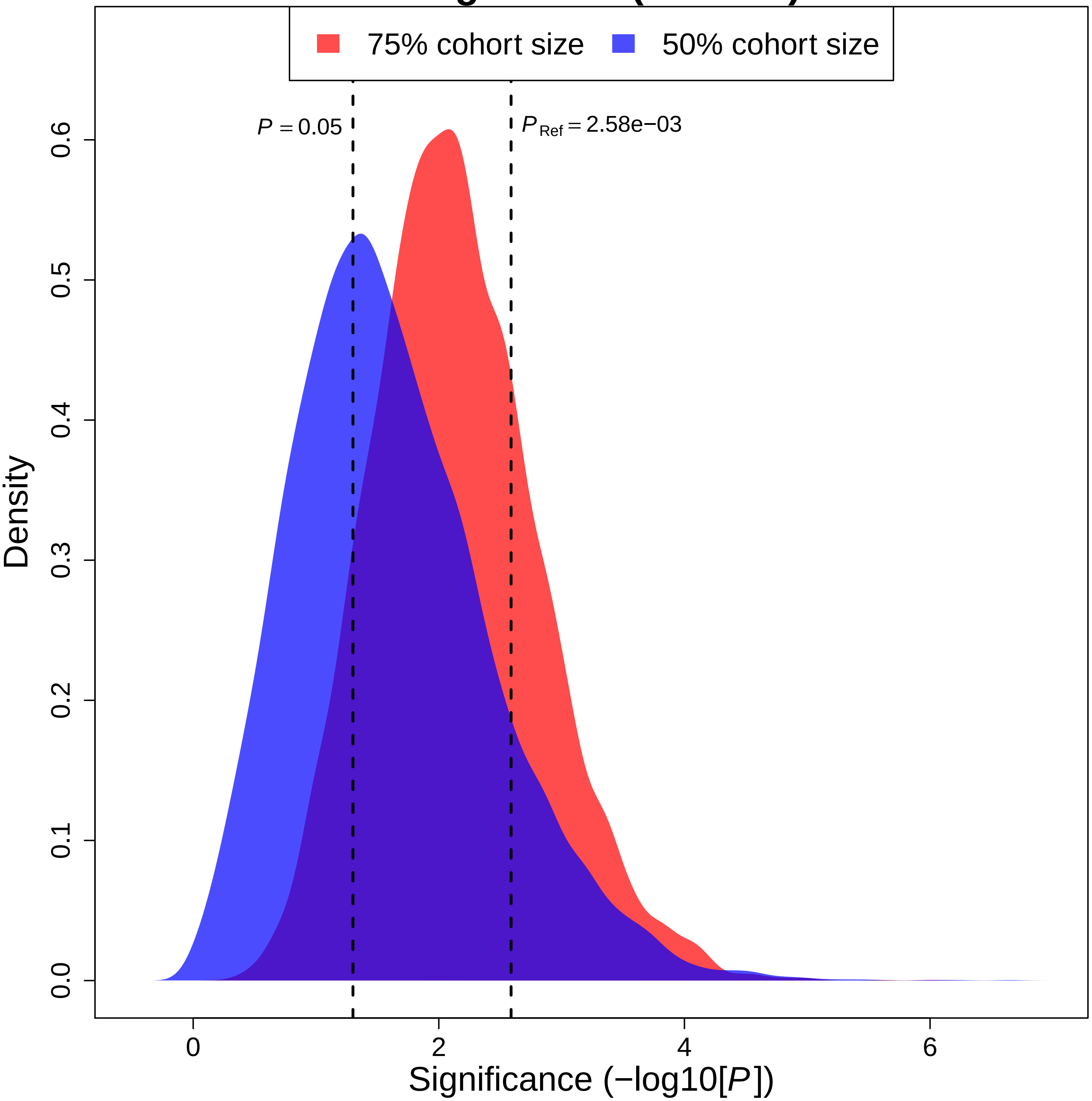

Cohort size: 442	$P > 0.05$	$P_{Ref} < P < 0.05$	$P < P_{Ref}$
75% (332)	9.78%	66.28%	23.94%
50% (221)	38.16%	49.99%	11.85%

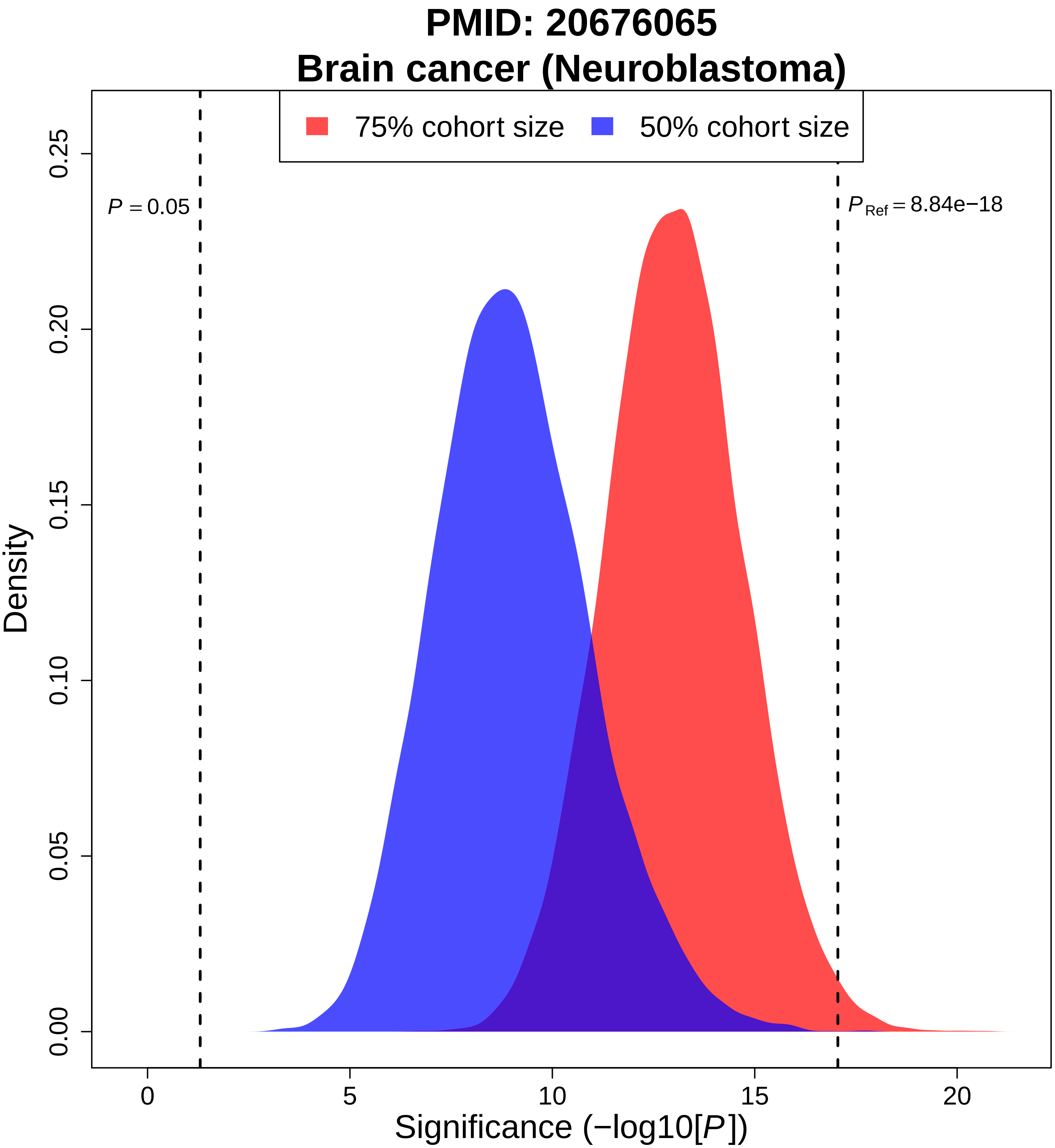

**Cohort size: 478**	$P > 0.05$	$P_{Ref} < P < 0.05$	$P < P_{Ref}$
75% (358)	0%	99.06%	0.94%
50% (239)	0%	99.98%	0.02%

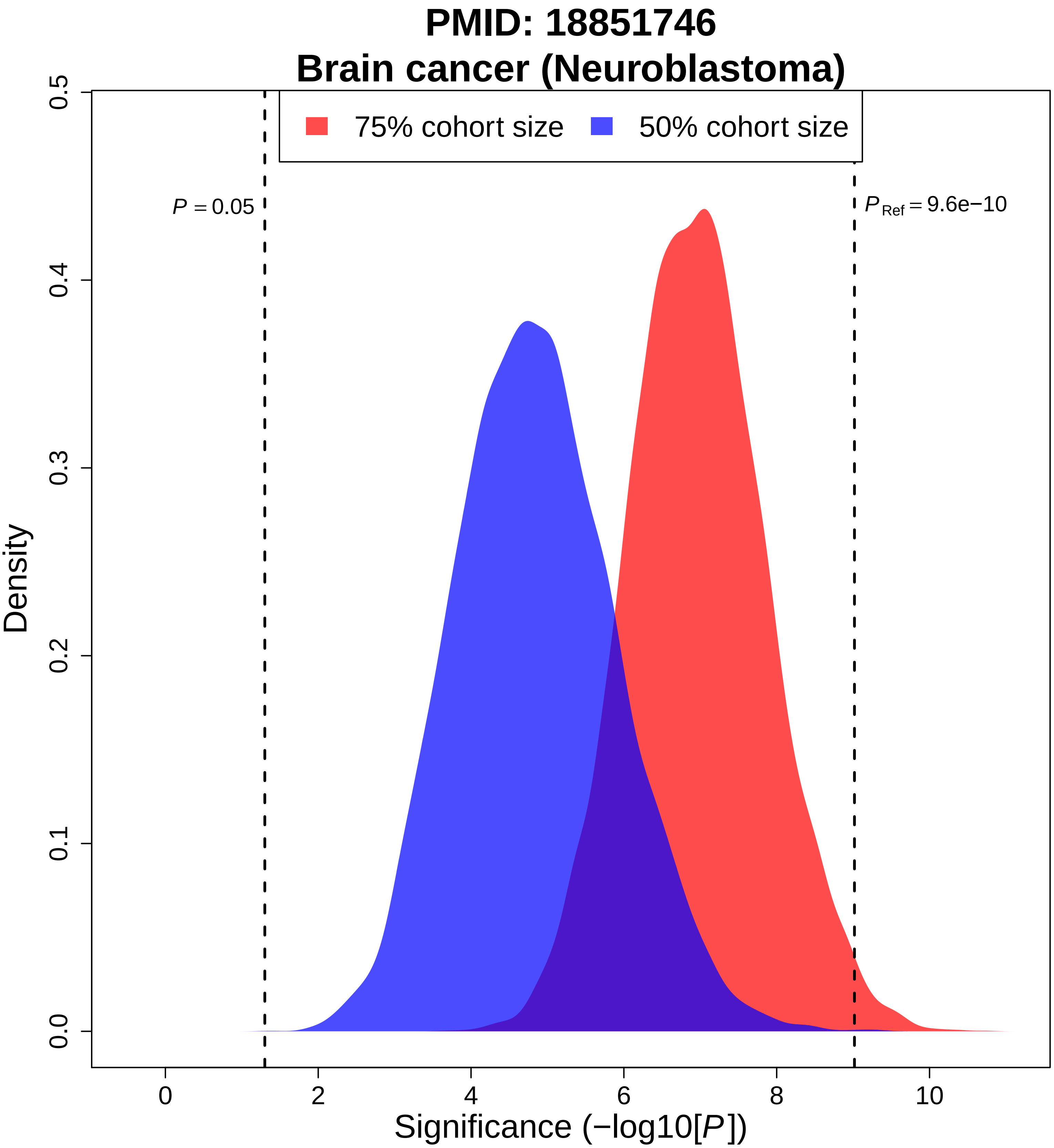

**Cohort size: 251**	$P > 0.05$	$P_{Ref} < P < 0.05$	$P < P_{Ref}$
75% (188)	0%	98.72%	1.28%
50% (126)	0%	99.96%	0.04%

PMID: 19038878
Hematopoietic cancer (DLBCL)

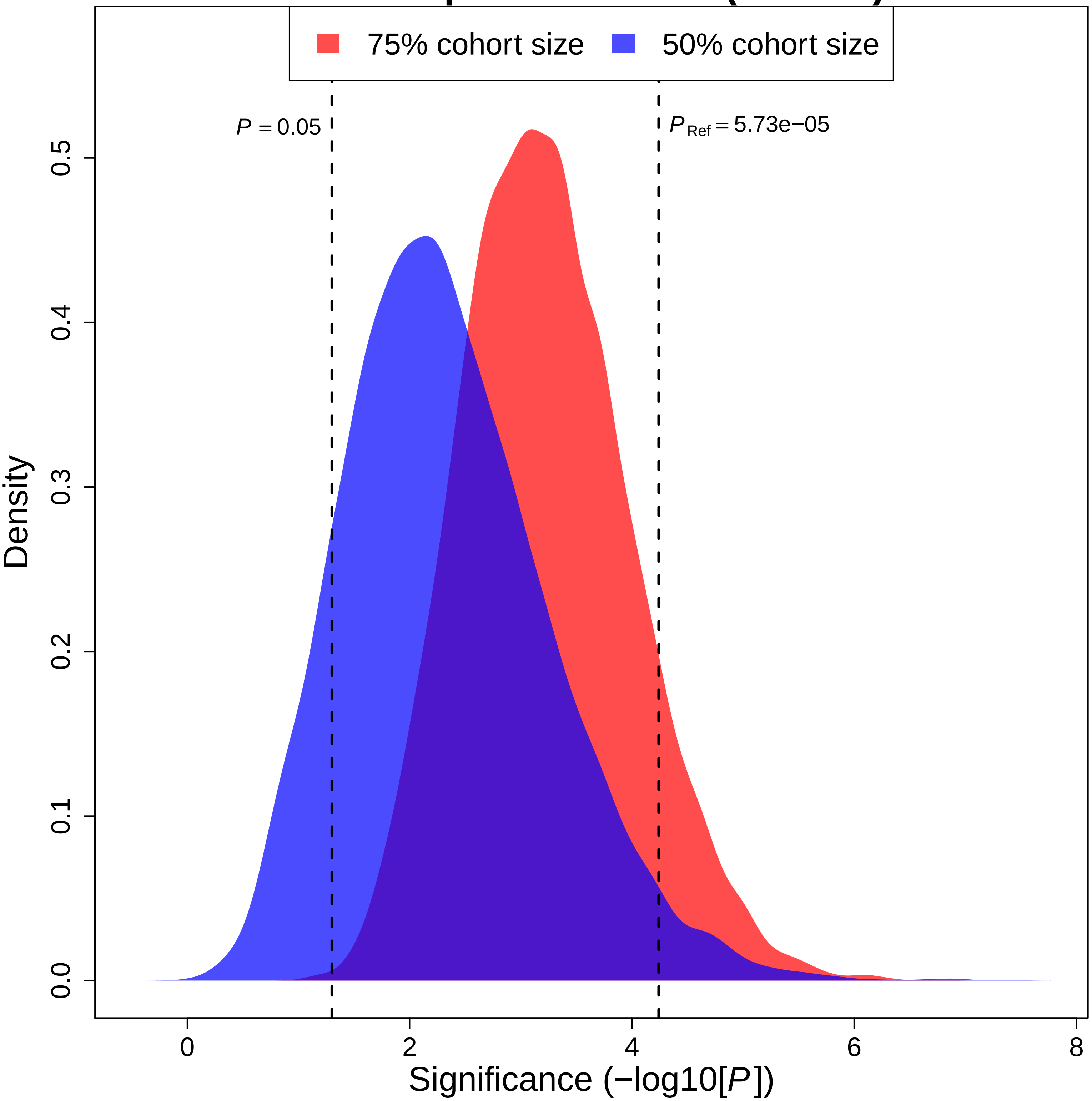

Cohort size: 414	$P > 0.05$	$P_{Ref} < P < 0.05$	$P < P_{Ref}$
75% (310)	0.1%	90.22%	9.68%
50% (207)	11.89%	85.12%	2.99%

PMID: 17410195
Hematopoietic cancer (B ALL)

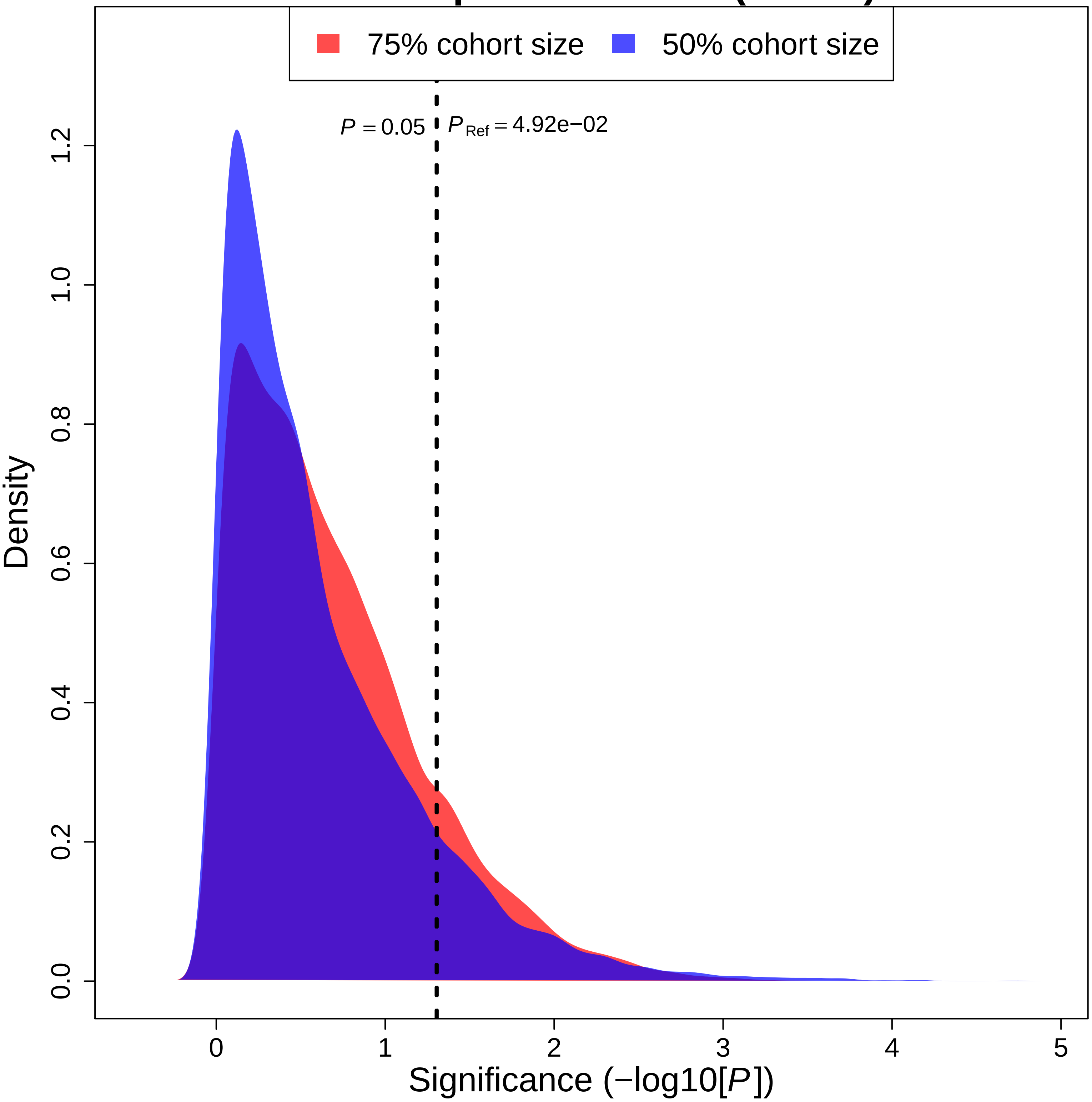

**Cohort size: 207**	$P > 0.05$	$P_{Ref} < P < 0.05$	$P < P_{Ref}$
75% (155)	85.89%	0.19%	13.92%
50% (104)	88.23%	0.13%	11.64%

PMID: 19192944
Ovarian cancer

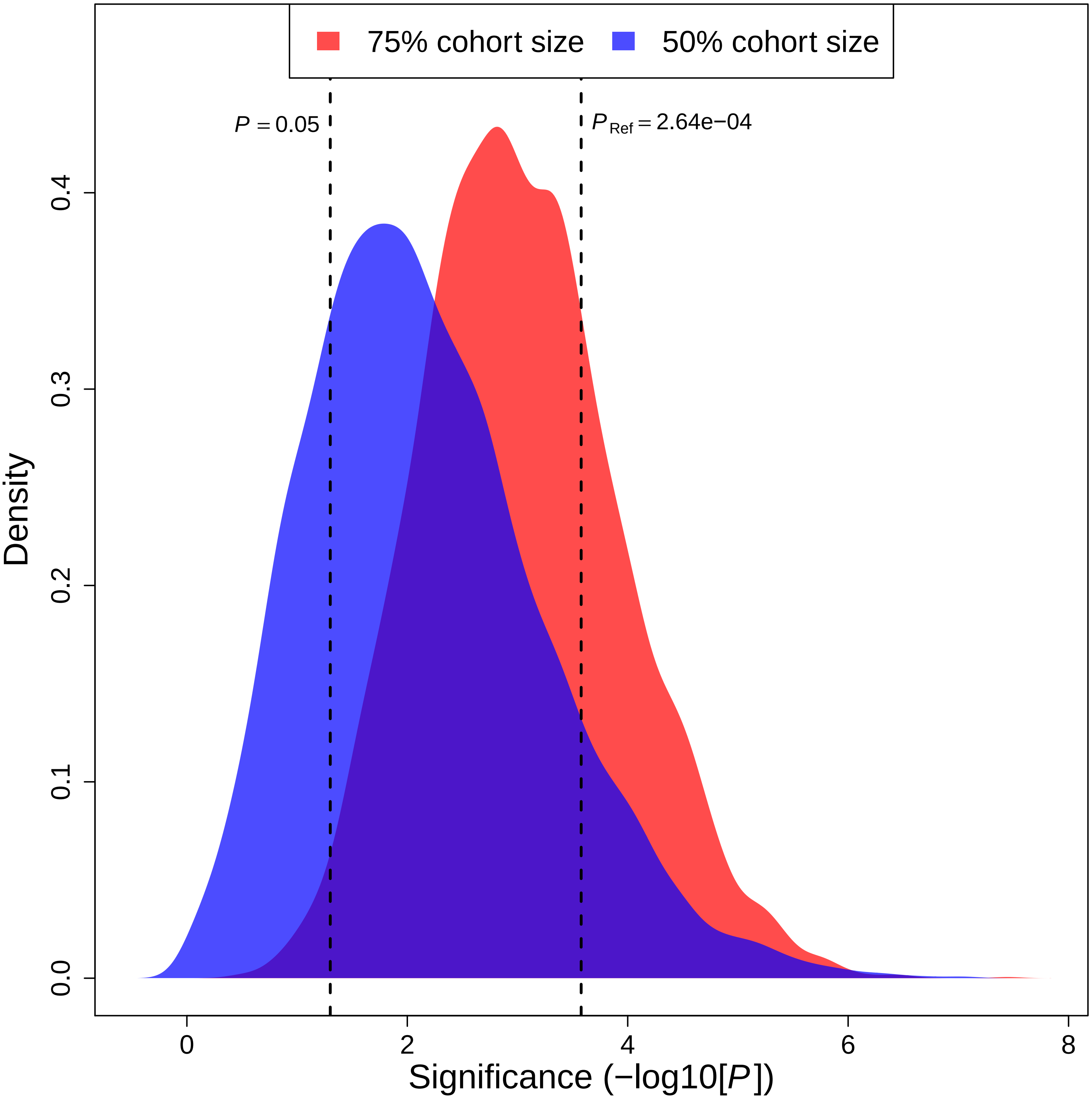

**Cohort size: 415**	$P > 0.05$	$P_{Ref} < P < 0.05$	$P < P_{Ref}$
75% (311)	1.62%	72.09%	26.29%
50% (208)	22.04%	67.58%	10.38%

PMID: 20233430
Prostate cancer

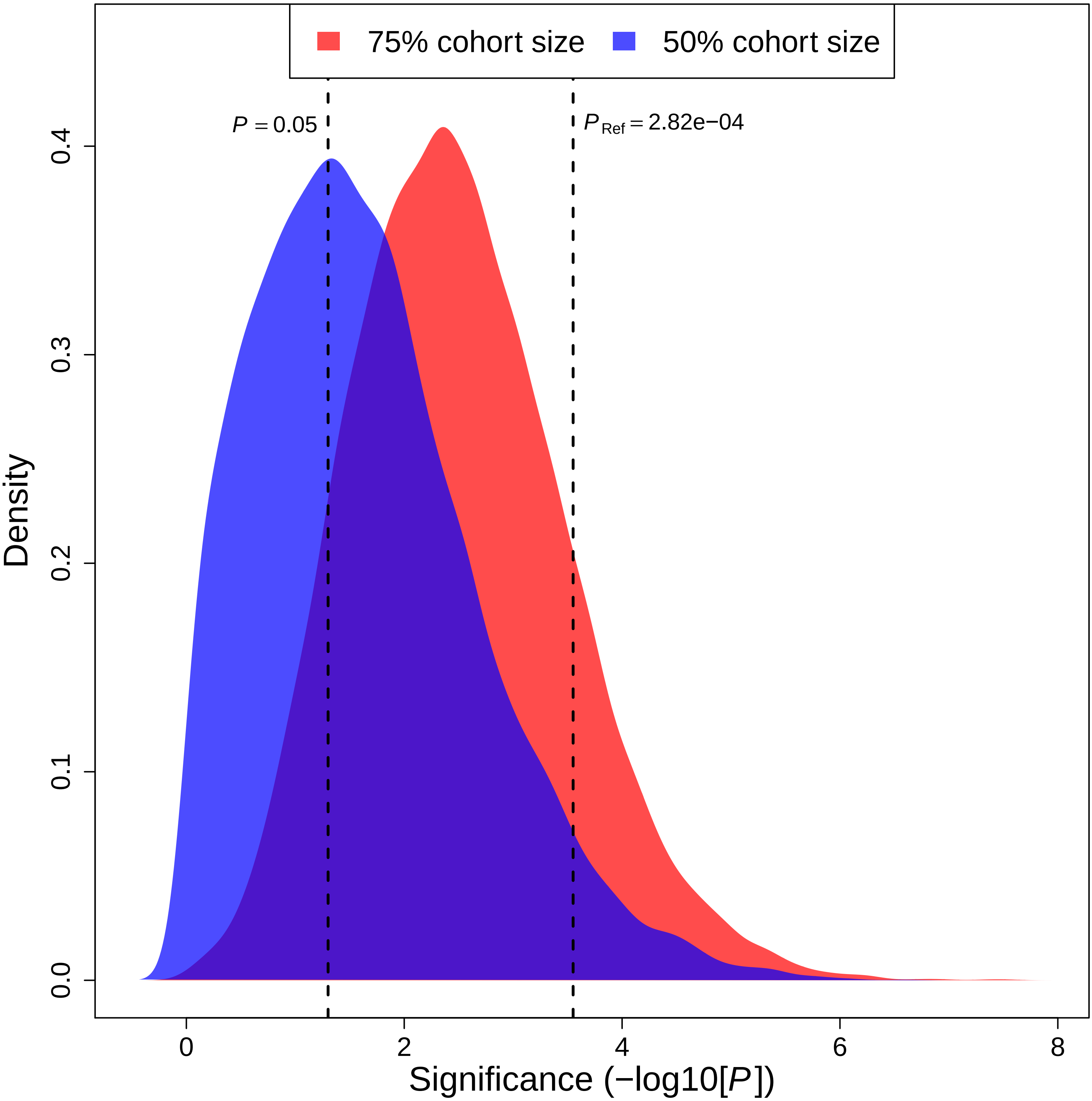

**Cohort size: 281**	$P > 0.05$	$P_{Ref} < P < 0.05$	$P < P_{Ref}$
75% (211)	10.31%	75.3%	14.39%
50% (140)	41.89%	53.4%	4.71%

PMID: 17023574
Hematopoietic cancer (Multiple myeloma)

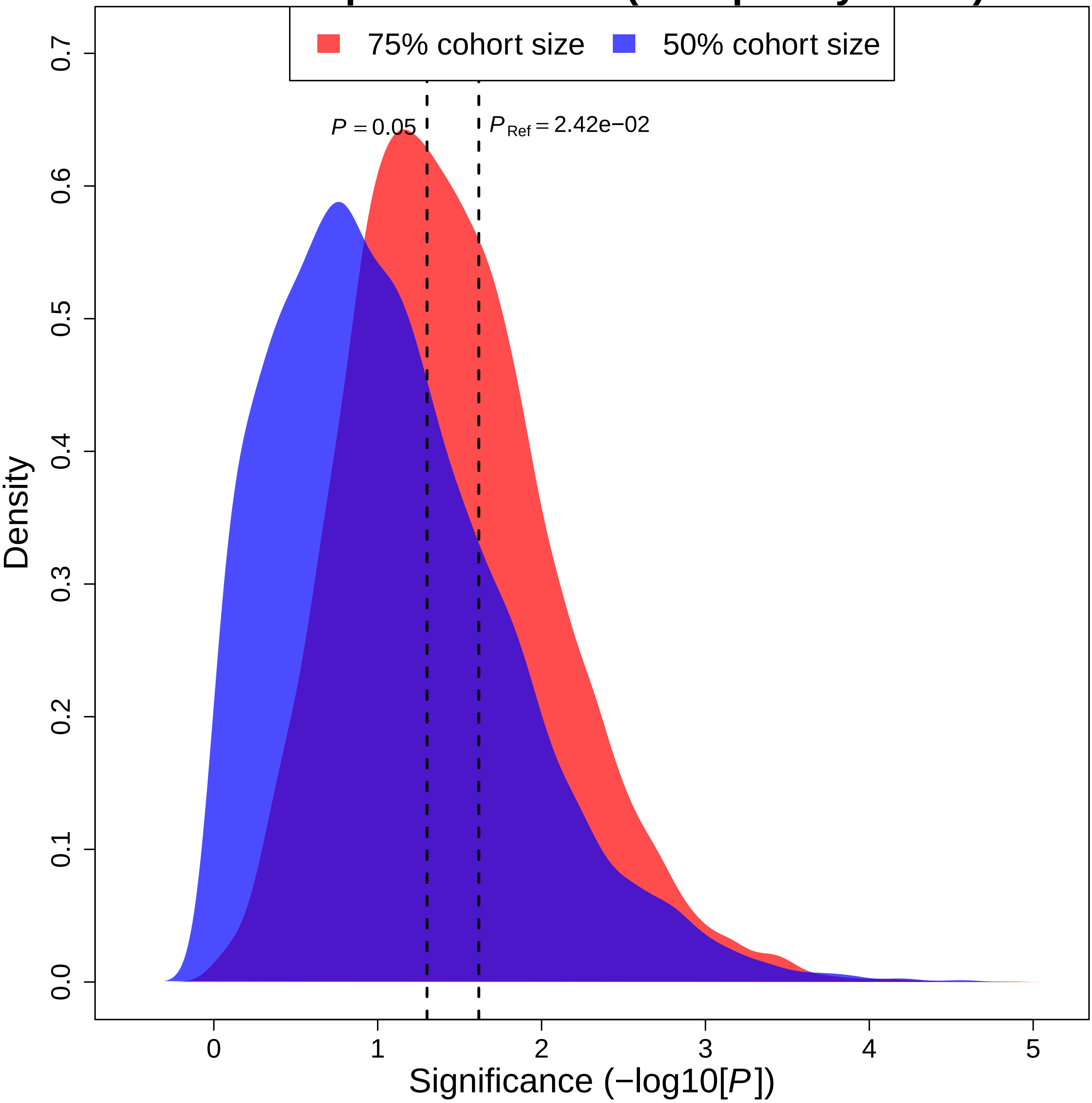

**Cohort size: 553**	$P > 0.05$	$P_{Ref} < P < 0.05$	$P < P_{Ref}$
75% (415)	44.55%	18.75%	36.7%
50% (276)	66.52%	12.26%	21.22%

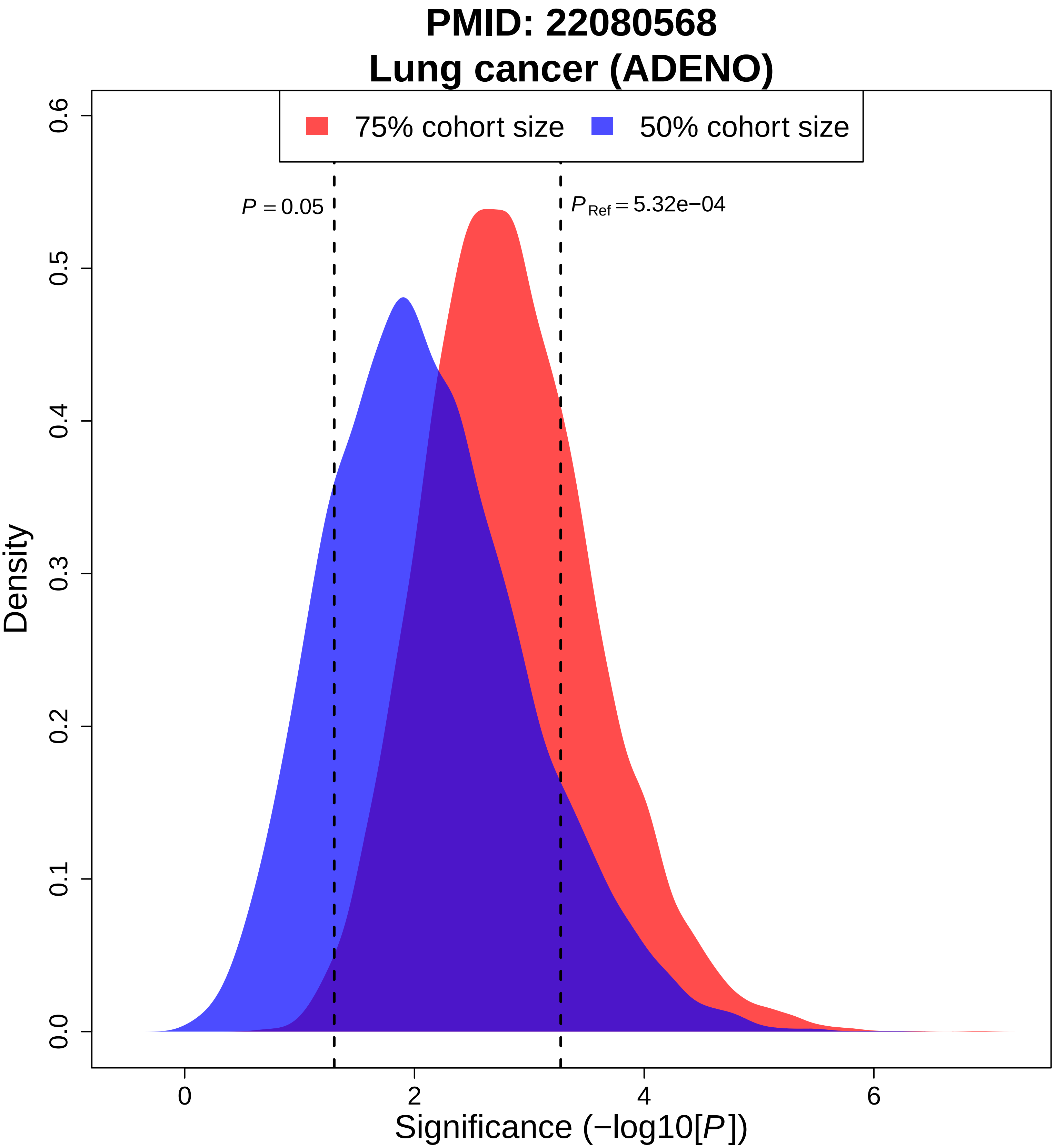

**Cohort size: 226**	$P > 0.05$	$P_{Ref} < P < 0.05$	$P < P_{Ref}$
75% (170)	0.89%	72.85%	26.26%
50% (113)	17.61%	72.29%	10.1%

PMID: 21720365
Ovarian cancer

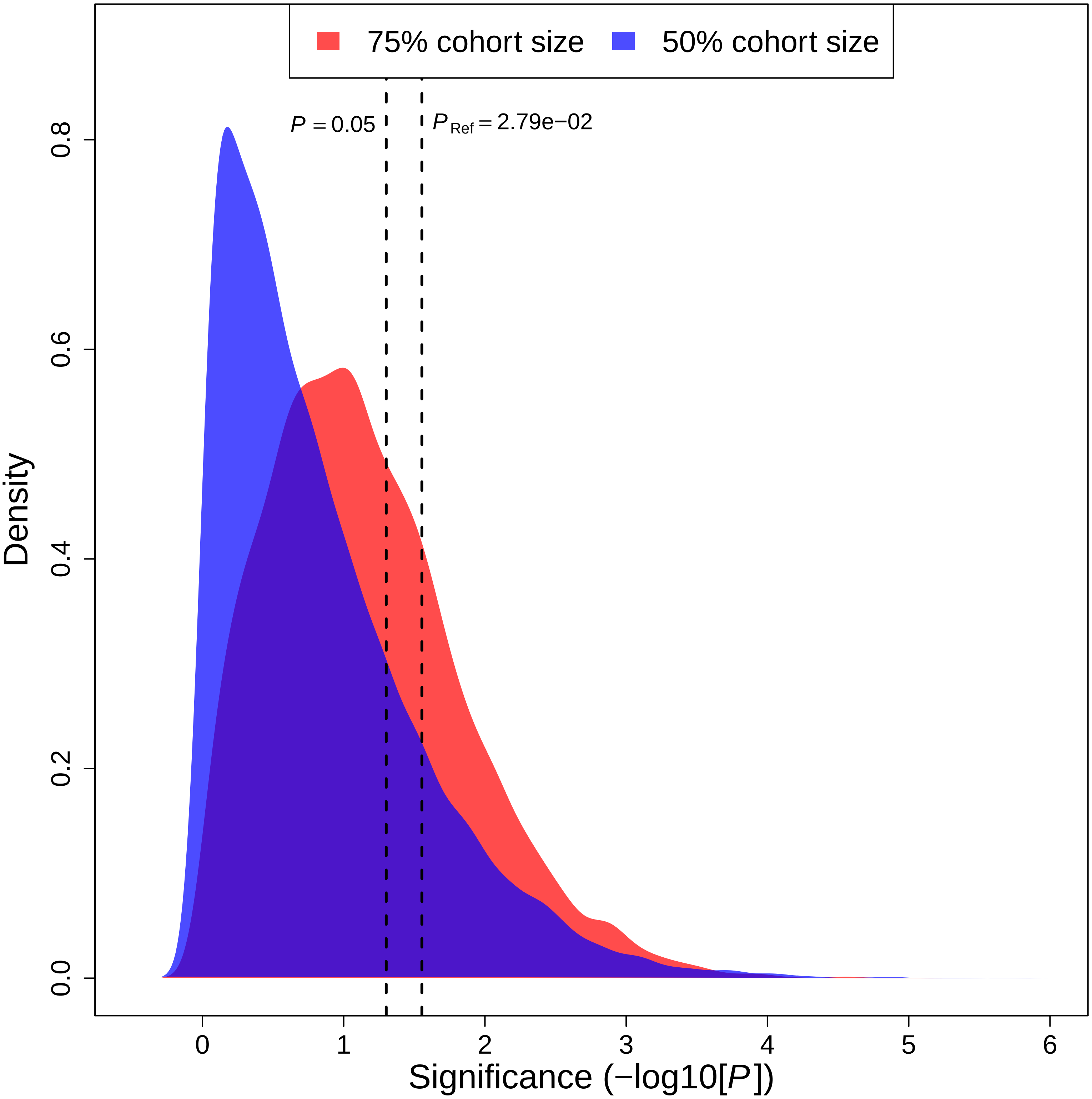

**Cohort size: 503**	$P > 0.05$	$P_{Ref} < P < 0.05$	$P < P_{Ref}$
75% (377)	62.58%	11.69%	25.73%
50% (252)	78.63%	6.59%	14.78%

PMID: 12490681
Breast cancer

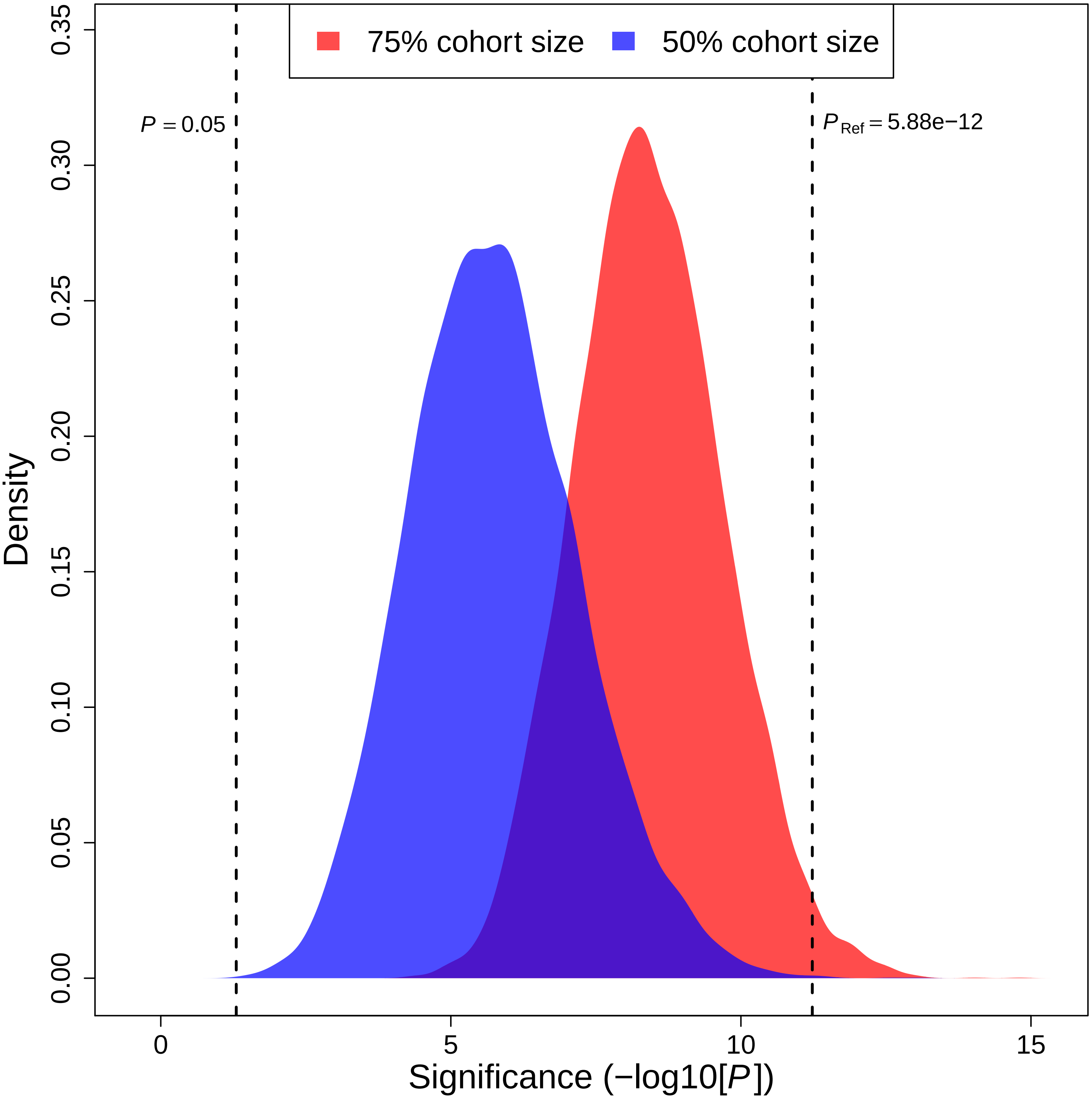

Cohort size: 295	$P > 0.05$	$P_{Ref} < P < 0.05$	$P < P_{Ref}$
75% (221)	0%	98.21%	1.79%
50% (148)	0%	99.95%	0.05%

Supplementary table 2

PRECOG ID	PMID	Accession	Platform	Cancer type	Cancer subtype	Outcome	P	HR	HR CI	CINSARC genes	Cohort size
30	20551037	GSE20486	GPL6947	Breast cancer		OS	1.04e−03	2.77	1.47−5.24	67	97
17	12670911	Nutt Glioma	HGU95Av2 EntrezCDF	Brain cancer	Glioma	OS	1.12e−01	1.82	0.86−3.82	46	50
111	11707567	ca00191	HGU95Av2 EntrezCDF	Lung cancer	ADENO	OS	1.18e−01	1.46	0.91−2.34	46	125
27	19435916	GSE10885	GPL887	Breast cancer		OS	1.23e−01	2.22	0.8−6.2	62	52
14	18565887	GSE7696	HGU133Plus2 EntrezCDF	Brain cancer	Glioblastoma	OS	1.29e−01	0.68	0.42−1.12	67	80
166	21372215	GSE21257	GPL10295	Sarcoma	Osteosarcoma	OS	1.48e−01	1.88	0.79−4.46	67	53
65	23634996	TCGA AML	HGU133Plus2 EntrezCDF	Hematopoietic cancer	AML	OS	1.50e−01	1.33	0.9−1.96	67	182
153	16273092	GSE3149	HGU133A EntrezCDF	Ovarian cancer		OS	1.58e−03	2.09	1.31−3.34	62	133
143	18505921	GSE8401	HGU133A EntrezCDF	Melanoma	Metastasis	OS	1.62e−01	0.64	0.33−1.21	62	67
125	20421987	GSE19188	HGU133Plus2 EntrezCDF	Lung cancer	SCC	OS	1.63e−01	1.5	0.85−2.66	67	82
28	19435916	GSE10885	GPL1390	Breast cancer		OS	1.75e−01	1.94	0.73−5.14	50	93
150	19294737	GSE14764	HGU133A EntrezCDF	Ovarian cancer		OS	1.79e−02	0.33	0.12−0.86	62	80
129	17082175	GSE5123	GPL3877	Lung cancer	SCC	OS	1.80e−01	1.68	0.78−3.59	56	51
128	16549822	GSE11969	GPL7015	Lung cancer	SCC	OS	1.89e−02	1.74	1.09−2.77	54	149
75	16760442	GSE4475	HGU133A EntrezCDF	Hematopoietic cancer	DLBCL	OS	1.90e−02	1.72	1.09−2.72	62	159
58	21989116	GSE31056	HGU133Plus2 EntrezCDF	Head and neck cancer	Oral SCC	OS	1.94e−02	2.16	1.12−4.17	67	96
136	16885343	GSE4573	GPL96	Lung cancer	SCC	OS	1.97e−01	0.73	0.45−1.18	61	130
159	20644708	GSE21501	GPL4133	Pancreatic cancer		OS	2.16e−01	1.36	0.83−2.21	66	102
126	21742808	GSE29013	HGU133Plus2 EntrezCDF	Lung cancer	SCC	OS	2.20e−01	1.84	0.69−4.9	67	55
32	21364938	GSE19783	GPL6480	Breast cancer		OS	2.28e−01	1.41	0.81−2.45	67	112
109	12118244	ca00153	Hu6800 EntrezCDF	Lung cancer	ADENO	OS	2.32e−01	1.65	0.72−3.8	29	86
21	20015288	GSE16581	HGU133Plus2 EntrezCDF	Brain cancer	Meningioma	OS	2.34e−01	0.4	0.08−1.92	67	67
87	17023574	GSE24080	HGU133Plus2 EntrezCDF	Hematopoietic cancer	Multiple myeloma	DSS	2.42e−02	1.59	1.06−2.38	67	553
72	21625232	GSE22762	HGU133Plus2 EntrezCDF	Hematopoietic cancer	CLL	OS	2.52e−02	2.67	1.09−6.52	67	107
135	19118056	Roepman LungCancer	Roepman	Lung cancer	SCC	OS	2.54e−02	1.91	1.07−3.41	55	171
114	18641660	ca00182	HGU133A EntrezCDF	Lung cancer	ADENO	OS	2.58e−03	1.5	1.15−1.95	62	442
157	19192944	GSE13876	GPL7759	Ovarian cancer		OS	2.64e−04	1.53	1.21−1.93	66	415
81	15345589	Glas FL	Glas FL	Hematopoietic cancer	FL	OS	2.65e−03	2.66	1.38−5.12	38	75
158	21720365	TCGA Ovarian	HTHGU133A EntrezCDF	Ovarian cancer		OS	2.79e−02	1.31	1.03−1.67	62	503
161	20233430	GSE16560	GPL5474	Prostate cancer		OS	2.82e−04	1.68	1.27−2.23	41	281
132	20643781	GSE17710	GPL9053	Lung cancer	SCC	OS	2.86e−01	1.5	0.71−3.17	66	56
66	19171880	GSE14468	HGU133Plus2 EntrezCDF	Hematopoietic cancer	AML	OS	2.98e−01	1.17	0.87−1.59	67	262
31	20098429	GSE16446	HGU133Plus2 EntrezCDF	Breast cancer		OS	3.19e−01	0.59	0.21−1.67	67	107
64	18270328	GSE10358	HGU133Plus2 EntrezCDF	Hematopoietic cancer	AML	OS	3.46e−04	2.14	1.4−3.29	67	178
155	22241791	GSE32062	GPL6480	Ovarian cancer		OS	3.59e−01	1.19	0.82−1.71	67	260
74	18615101	E−TABM−346	HGU133A EntrezCDF	Hematopoietic cancer	DLBCL	OS	3.62e−01	1.39	0.68−2.85	62	53
76	16760443	GSE4732	GPL3706	Hematopoietic cancer	DLBCL	OS	3.62e−01	1.16	0.84−1.61	27	272
29	21586611	GSE29174	GPL3676	Breast cancer		OS	3.76e−01	0.72	0.34−1.5	56	96
123	18486272	GSE10245	HGU133Plus2 EntrezCDF	Lung cancer	SCC	OS	3.81e−01	0.69	0.3−1.6	67	58
36	17545524	GSE7390	HGU133A EntrezCDF	Breast cancer		OS	4.04e−04	3.1	1.6−6	62	198
134	19010856	GSE8894	GPL570	Lung cancer	SCC	OS	4.17e−02	1.83	1.01−3.31	66	138
37	16273092	GSE3143	HGU95Av2 EntrezCDF	Breast cancer		OS	4.31e−02	1.82	1.01−3.28	46	158
148	19962670	GSE18521	HGU133Plus2 EntrezCDF	Ovarian cancer		OS	4.33e−01	0.77	0.41−1.44	67	53
39	21542898	GSE24450	GPL6947	Breast cancer		OS	4.66e−04	3.52	1.66−7.45	67	183
133	17601969	GSE5828	GPL3877	Lung cancer	SCC	OS	4.72e−01	0.77	0.37−1.58	54	59
46	21619627	GSE24549	GPL5175	Colon cancer		DSS	4.83e−01	0.8	0.43−1.48	67	83
68	17410195	GSE11877	HGU133Plus2 EntrezCDF	Hematopoietic cancer	B ALL	OS	4.92e−02	1.89	0.99−3.61	67	207
93	18923165	GSE10143	GPL5474	Liver cancer		OS	4.96e−02	1.76	0.99−3.13	41	144
59	18716133	GSE12417	HGU133Plus2 EntrezCDF	Hematopoietic cancer	AML	OS	5.19e−01	0.83	0.46−1.47	67	79
149	16204010	GSE31245	HGU95Av2 EntrezCDF	Ovarian cancer		OS	5.19e−01	0.77	0.34−1.73	46	57
113	22080568	GSE31210	HGU133Plus2 EntrezCDF	Lung cancer	ADENO	OS	5.32e−04	3.52	1.65−7.52	67	226
130	20823422	GSE14814	HGU133A EntrezCDF	Lung cancer	SCC	OS	5.49e−01	0.82	0.44−1.56	62	90
154	18593951	GSE26712	HGU133A EntrezCDF	Ovarian cancer		DSS	5.67e−01	0.9	0.63−1.28	62	185
80	19038878	GSE10846	HGU133Plus2 EntrezCDF	Hematopoietic cancer	DLBCL	OS	5.73e−05	1.94	1.4−2.69	67	414
67	17312329	GSE5314	GPL3999	Hematopoietic cancer	B ALL	OS	5.85e−01	1.19	0.64−2.22	54	53
41	12490681	Vijver BreastCancer	Vijver Agilent	Breast cancer		OS	5.88e−12	6.02	3.37−10.75	57	295
142	20460471	GSE22155	GPL6102	Melanoma		OS	6.08e−02	1.75	0.97−3.18	65	54
83	18416826	GSE10793	GPL3278	Hematopoietic cancer	Mantle cell lymphoma	OS	6.21e−06	4.09	2.13−7.83	25	71
139	15016488	GSE1037	GPL962	Lung cancer	SCLC	OS	6.33e−02	2.41	0.93−6.3	55	61
20	11807556	Pomeroy CNS	GPL80	Brain cancer	Medulloblastoma	OS	6.37e−01	0.8	0.32−1.99	28	60
156	18698038	GSE9899	HGU133Plus2 EntrezCDF	Ovarian cancer		OS	6.46e−01	1.09	0.75−1.59	67	278
44	19399471	GSE12945	HGU133A EntrezCDF	Colon cancer		OS	6.72e−01	0.78	0.25−2.46	62	62
6	20059769	GSE13507	GPL6102	Bladder cancer		OS	6.86e−04	2.37	1.42−3.95	65	165
131	16273092	GSE3141	HGU133Plus2 EntrezCDF	Lung cancer	SCC	OS	7.17e−01	0.91	0.54−1.53	67	111
152	20300634	GSE17260	GPL6848	Ovarian cancer		OS	7.37e−01	1.1	0.62−1.98	66	110
92	15565109	GSE1898	GPL1528	Liver cancer		OS	7.38e−04	2.87	1.52−5.44	66	76
47	19914252	GSE17536	HGU133Plus2 EntrezCDF	Colon cancer		OS	7.40e−01	1.08	0.68−1.73	67	177
34	19204204	GSE10886	GPL1390	Breast cancer		OS	7.77e−01	0.91	0.47−1.75	60	178
45	21619627	GSE24550	GPL5175	Colon cancer		DSS	8.15e−03	0.1	0.01−0.81	67	77
137	22960745	TCGA LUSC	HTHGU133A EntrezCDF	Lung cancer	SCC	OS	8.18e−01	1.07	0.61−1.88	62	132
35	18347175	GSE9893	GPL5049	Breast cancer		OS	8.20e−07	4.12	2.24−7.58	32	155
54	20458058	Thurlow HNSCC	HGU133Plus2 EntrezCDF	Head and neck cancer		DSS	8.36e−01	1.08	0.52−2.24	67	74
85	17409404	GSE6477	HGU133A EntrezCDF	Hematopoietic cancer	Multiple myeloma	OS	8.47e−04	2.19	1.37−3.52	62	145
43	19914252	GSE17537	HGU133Plus2 EntrezCDF	Colon cancer		OS	8.73e−01	0.93	0.39−2.25	67	55
24	20676065	E−MTAB−179	NBCustomAgilent4x44K	Brain cancer	Neuroblastoma	OS	8.84e−18	6.46	3.96−10.54	67	478
16	18772890	TCGA Glioblastoma	HTHGU133A EntrezCDF	Brain cancer	Glioblastoma	OS	9.01e−01	1.01	0.8−1.29	62	348
151	19047114	GSE8842	GPL5689	Ovarian cancer		OS	9.12e−01	0.94	0.34−2.65	42	83
63	16597596	ca00119	HGU95Av2 EntrezCDF	Hematopoietic cancer	AML	OS	9.14e−02	1.33	0.95−1.85	46	169
77	11786909	Shipp DLBCL	Hu6800 EntrezCDF	Hematopoietic cancer	DLBCL	OS	9.34e−01	1.03	0.47−2.26	29	58
112	19414676	GSE13213	GPL6480	Lung cancer	ADENO	OS	9.46e−04	2.61	1.45−4.7	67	117
23	18851746	E−TABM−38	A−MEXP−255	Brain cancer	Neuroblastoma	OS	9.60e−10	21.92	5.27−91.23	55	251
138	22960745	TCGA LUSC	AgilentG4502A Gene	Lung cancer	SCC	OS	9.67e−01	0.99	0.58−1.68	57	151
18	19244127	ca00037	HGU133Plus2 EntrezCDF	Brain cancer	Glioma	OS	9.87e−01	1.01	0.52−1.94	67	147
